# Transcriptomic identification of starfish neuropeptide precursors yields new insights into neuropeptide evolution

**DOI:** 10.1098/rsob.150224

**Published:** 2016-02-10

**Authors:** Dean C. Semmens, Olivier Mirabeau, Ismail Moghul, Mahesh R. Pancholi, Yannick Wurm, Maurice R. Elphick

**Affiliations:** 1 School of Biological and Chemical Sciences, Queen Mary University of London, Mile End Road, London E1 4NS, UK; 2 Institut Curie, Genetics and Biology of Cancers Unit, INSERM U830, PSL Research University, Paris 75005, France

**Keywords:** neuropeptide, evolution, deuterostome, echinoderm, starfish

## Abstract

Neuropeptides are evolutionarily ancient mediators of neuronal signalling in nervous systems. With recent advances in genomics/transcriptomics, an increasingly wide range of species has become accessible for molecular analysis. The deuterostomian invertebrates are of particular interest in this regard because they occupy an ‘intermediate' position in animal phylogeny, bridging the gap between the well-studied model protostomian invertebrates (e.g. *Drosophila melanogaster*, *Caenorhabditis elegans*) and the vertebrates. Here we have identified 40 neuropeptide precursors in the starfish *Asterias rubens*, a deuterostomian invertebrate from the phylum Echinodermata*.* Importantly, these include kisspeptin-type and melanin-concentrating hormone-type precursors, which are the first to be discovered in a non-chordate species. Starfish tachykinin-type, somatostatin-type, pigment-dispersing factor-type and corticotropin-releasing hormone-type precursors are the first to be discovered in the echinoderm/ambulacrarian clade of the animal kingdom. Other precursors identified include vasopressin/oxytocin-type, gonadotropin-releasing hormone-type, thyrotropin-releasing hormone-type, calcitonin-type, cholecystokinin/gastrin-type, orexin-type, luqin-type, pedal peptide/orcokinin-type, glycoprotein hormone-type, bursicon-type, relaxin-type and insulin-like growth factor-type precursors. This is the most comprehensive identification of neuropeptide precursor proteins in an echinoderm to date, yielding new insights into the evolution of neuropeptide signalling systems. Furthermore, these data provide a basis for experimental analysis of neuropeptide function in the unique context of the decentralized, pentaradial echinoderm bauplan.

## 1. Background

Neuropeptides are intercellular signalling molecules that are secreted by neurons to act as neurotransmitters, modulators of synaptic transmission or hormones. They range in size from just three amino acids, such as thyrotropin-releasing hormone (TRH), to much longer polypeptides (e.g. neuropeptide Y, which comprises 36 residues). However, all neuropeptides share the common characteristic of being derived from larger precursor proteins, which have an N-terminal signal peptide that targets the precursor protein to the regulated secretory pathway. Neuropeptides are key players in neural mechanisms controlling physiological and behavioural processes; for example, neuropeptides control feeding behaviour and reproductive behaviour in vertebrates and invertebrates [1,2]. Furthermore, the evolutionary origins of neuropeptides as regulators of physiology and behaviour are ancient; for example, neuropeptide signalling pathways are key components of the nervous systems of basal animal phyla such as the cnidarians [3], and the origins of some peptide signalling pathways may pre-date the emergence of animals with nervous systems [4].

A huge variety of neuropeptides have been identified in vertebrates and invertebrates, but establishing evolutionary relationships between neuropeptides identified in different phyla has proved to be quite difficult because they comprise relatively short stretches of amino acids, typically with only a few conserved residues. However, recent advances in comparative genomics/transcriptomics are transforming our understanding of the evolutionary and functional significance of neuropeptide diversity in animals. Thus, a core set of neuropeptide-receptor signalling pathways have been traced back to the common ancestor of the Bilateria, with families of orthologous neuropeptides being identified in an increasingly wide range of animal phyla [5,6].

**Figure 1.**
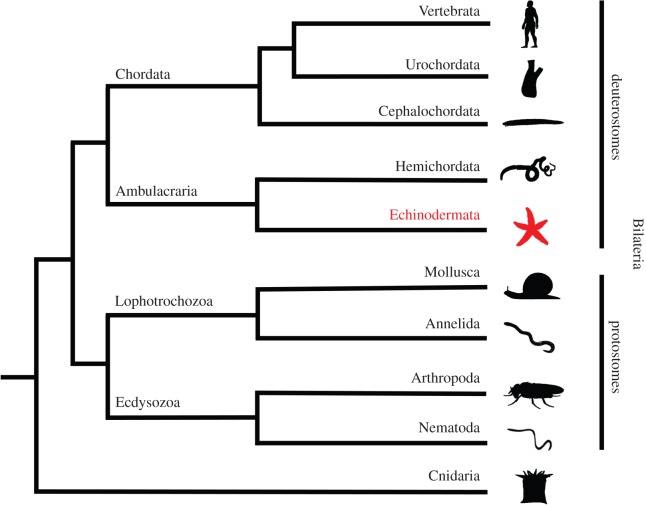
Animal phylogeny. Phylogenetic diagram showing the position of the phylum Echinodermata (shown in red; e.g. starfish) in the deuterostomian branch of the animal kingdom. The Bilateria comprise two super-phyla—the deuterostomes and the protostomes. The deuterostomes comprise the chordates (vertebrates, urochordates and cephalochordates) and the ambulacrarians (hemichordates and echinoderms). The protostomes comprise the lophotrochozoans (e.g. molluscs and annelids) and the ecdysozoans (e.g. arthropods and nematodes). The Cnidaria (e.g. sea anemones) are basal to the Bilateria. Images of representative animals from each phylum were obtained from http://phylopic.org or were created by the authors or by M. Zandawala (Stockholm University).

The classical invertebrate model systems *Drosophila melanogaster* and *Caenorhabditis elegans* have been and continue to be important for neuropeptide research [1,2]. However, both species belong to phyla in the ecdysozoan clade of the animal kingdom and therefore they are not representative of invertebrates as a whole (figure 1). Critical to recent breakthroughs in our knowledge and understanding of neuropeptide evolution has been the analysis of genome/transcriptome data from other invertebrates, and in particular lophotrochozoans (annelids and molluscs) and ambulacrarians (echinoderms and hemichordates) [5–10]. Thus, we are entering a new era where we have a molecular phylogenetic framework that enables investigation of how evolutionarily ancient orthologous neuropeptide systems are used to regulate physiological and behavioural processes in animals from a range of phyla.

The echinoderms (e.g. starfish, sea urchins, sea cucumbers) are particularly interesting for comparative and evolutionary studies on neuropeptide signalling systems for a variety of reasons. They are deuterostomian invertebrates and therefore by virtue of their close relationship with chordates (figure 1), echinoderms can provide key insights into the evolution of neuropeptide systems in the animal kingdom. For example, the recent discovery of a neuropeptide precursor in the sea urchin *Strongylocentrotus purpuratus* comprising multiple copies of TRH-type peptides revealed for the first time that the evolutionary origin of TRH-type neuropeptides extends beyond the vertebrates to invertebrates [9]. Furthermore, echinoderms have the unique characteristic in the animal kingdom of exhibiting pentaradial symmetry as adult animals, which is derived from a bilateral body plan both evolutionarily and developmentally. Consequently, echinoderms do not have a ‘brain’; the nervous system is decentralized, with the control of whole-animal behaviour co-ordinated by five radial nerve cords that are linked by a circumoral nerve ring [11,12]. Thus, it is of interest to determine how different neuropeptide signalling systems are organized and used to regulate physiological and behavioural processes in the context of the highly derived (pentaradial) and decentralized nervous systems of echinoderms. Relevant to this issue, there is evidence that neuropeptides may be involved in mediating neural control of several unusual biological phenomena in echinoderms. The ability to autotomize and then regenerate body parts is one of the most remarkable characteristics of echinoderms and it has been reported that arm autotomy in starfish is triggered by a peptide(s), but its molecular identity is unknown [13]. Another unusual feature of echinoderms is the mutability of their collagenous tissue, which can rapidly change between stiff and soft mechanical states under the control of the nervous system [14]. Neuropeptides that affect the stiffness of the body wall in sea cucumbers have been identified [15], but the mechanisms by which they exert effects are unknown [16].

The first extensive analysis of neuropeptide diversity in an echinoderm species was enabled by sequencing of the genome and transcriptome of *S. purpuratus*, and 28 candidate neuropeptide/peptide hormone precursors have been identified in this species to date [9]. These include, for example, homologues of vasopressin (VP)/oxytocin (OT), gonadotropin-releasing hormone (GnRH) and calcitonin (CT). At present, little is known about the physiological roles of these peptides in sea urchins; however, efforts to address this issue have commenced. For example, *in vitro* pharmacological studies have revealed that echinotocin, a VP/OT-type neuropeptide, causes contraction of the oesophagus and tube feet in sea urchins [17].

More recently, analysis of transcriptome sequence data has identified neuropeptide/peptide hormone precursors in a second echinoderm species, the sea cucumber *Apostichopus japonicus* [10]. Thus, we now have data from species representative of two of the five classes of extant echinoderms: Echinoidea (*S. purpuratus*) and Holothuroidea (*A. japonicus*). Analysis of phylogenetic relationships of the extant echinoderm classes indicates that echinoids and holothurians are sister groups in a clade known as the Echinozoa, while asteroids (starfish) and ophiuroids (brittle stars) are sister groups in a clade known as the Asterozoa, with crinoids (feather stars and sea lilies) occupying a basal position with respect to the echinozoa and Asterozoa [18,19]. Thus, our current knowledge of neuropeptide diversity in echinoderms based upon analysis of transcriptome/genome sequence data is restricted to the echinozoan clade. Deeper insights into the evolution and diversity of neuropeptide systems in echinoderms could be obtained by analysis of transcriptome/genome sequence data from asterozoans (starfish and brittle stars) and crinoids. To begin address this issue, here we have generated and analysed neural transcriptome data from a species belonging the class Asteroidea—the common European starfish *Asterias rubens*.

We have selected *A. rubens* as a model echinoderm for transcriptomic and experimental analysis of neuropeptide signalling systems for several reasons. First, *A. rubens* has been used as an experimental system for neuropeptide research for many years. Thus, the detection of FMRFamide-like immunoreactivity in the nervous system of *A. rubens* led to the discovery of the first neuropeptides to be identified in an echinoderm—the SALMFamides S1 and S2 [20–22]. Subsequently, detailed investigations of the expression [23–26] and pharmacological actions [26–28] of S1 and S2 in *A. rubens* have provided insights into the physiological roles of SALMFamides in echinoderms [29]. Second, *A. rubens* is a common and therefore easily obtained species of starfish in the UK and throughout much of coastal Europe—the range of *A. rubens* extends from the White Sea in Russia to the coast of Senegal. *Asterias rubens* also occurs in deeper waters off the northern coast of North America. Furthermore, closely related species of the genus *Asterias* occur globally—*Asterias forbesi* along the Atlantic coast of the USA from Maine to the Gulf of Mexico and *Asterias amurensis*, a Northern Pacific starfish native to the coasts of Japan, China, Korea and Russia (http://www.marinespecies.org/aphia.php?p=taxdetails&id=123776). Third, analysis of neuropeptide systems in *A. rubens* and other starfish species is also of potential interest from an applied perspective because of the economic/environmental impact of these animals as predators on shellfish (e.g. mussels; *A. rubens*) [30,31] and coral (*Acanthaster planci*) [32–34].

Here, we report the identification of 40 transcripts encoding neuropeptide precursors in *A. rubens* based on our analysis of neural transcriptome sequence data. Combined with our recent analysis of the neuropeptide transcriptome of the sea urchin *S. purpuratus* [9] and the sea cucumber *A. japonicus* [10], these data provide important new insights into the evolution and diversity of neuropeptide signalling systems. Furthermore, the data provide a basis for comprehensive analysis of the physiological roles of neuropeptides in starfish, employing *A. rubens* as a model experimental system.

## 2. Material and methods

### 2.1. Sequencing of *Asterias rubens* radial nerve transcriptome

Radial nerve cords (approx. 30 mg) dissected from a male adult specimen of *A. rubens* were used for RNA isolation (Total RNA Isolation System, Promega). Library preparation (TruSeqv2 kit, Illumina) was performed at the QMUL Genome Centre and sequencing was performed on an Illumina HiSeq platform at NIMR (Mill Hill), with cBot used to generate clusters. A total of 168 776 495 × 100 bp reads were obtained and raw sequence data (SRP068147; http://www.ncbi.nlm.nih.gov/sra/SRP068147) were assembled using SOAPdenovo-Trans v. 1.0 (http://soap.genomics.org.cn/SOAPdenovo-Trans.html), a short-read assembly method developed by the Beijing Genomics Institute [35]. Contigs were assembled from reads with an overlap greater than 31 bp, which were then mapped back to the raw reads. The 326 816 contigs generated (with 16 316 over 1000 bp) were then set up for BLAST analysis using SequenceServer, which is freely available to academic users (http://www.sequenceserver.com) [36].

### 2.2. BLAST-based identification of neuropeptide precursors in *Asterias rubens*

To search for transcripts encoding putative neuropeptide or peptide hormone precursor proteins in *A. rubens*, the sequences of neuropeptide or peptide hormone precursors previously identified in the sea urchin *S. purpuratus* [5,6,11,16,17,37,38], the sea cucumber *A. japonicus* [10] and the starfish species *Asterina pectinifera* [39] were submitted individually as queries in tBLASTn searches of the contig database with the BLAST parameter *e*-value set to 1000. Contigs identified as encoding putative precursors were analysed after translation of their full-length DNA sequence into protein sequence using the ExPASy Translate tool (http://web.expasy.org/translate/). Proteins were assessed as potential precursors of secreted bioactive peptides by investigating: (i) the presence of a putative N-terminal signal peptide sequence, using the SignalP v. 3.0 online server [40], (ii) the presence of putative monobasic or dibasic cleavage sites N-terminal and C-terminal to the putative bioactive peptide(s), with reference to known consensus cleavage motifs [41–43], and (iii) the presence, in some cases, of a C-terminal glycine residue that is a potential substrate for amidation.

### 2.3. De novo-based identification of candidate neuropeptide precursors in *Asterias rubens*

A list of potential ORFs that were generated from the *A. rubens* transcriptome sequence data were analysed using a hidden Markov model described in [44,45]. The top 500 candidate sequences were then screened for the presence of a signal peptide and short sequences flanked by canonical Gly-Lys-Arg motifs characteristic of prohormone convertase cleavage sites. The transcriptome sequence data were also analysed using a novel neuropeptide-prediction tool NpSearch, which uses characteristics of neuropeptide precursors (signal peptide, dibasic cleavage sites) to identify novel neuropeptides and their precursors (https://rubygems.org/gems/NpSearch) [46].

### 2.4. Analysis of the sequences of neuropeptide precursor transcripts identified in *Asterias rubens*

The protein sequences of candidate neuropeptide precursors and polypeptide hormone precursors were annotated in colour as follows. The N-terminal signal peptide, identified using SignalP v.3.0, was coloured blue; putative dibasic or monobasic cleavage sites were coloured green; and the putative neuropeptide(s) or peptide hormone(s) derived from the precursor was coloured red, with C-terminal glycine residues (when present) shown in orange. Figures compiling the colour-coded precursor sequences were prepared (figures 2, 9, 18 and 21). The DNA sequences of transcripts encoding precursor proteins were also compiled, together with the underlying encoded protein sequence (see electronic supplementary material, figures S1–S40).

**Figure 2.**
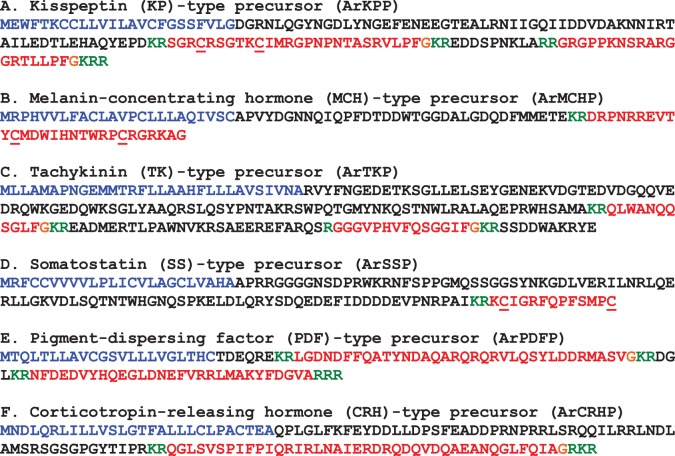
Precursors of neuropeptides in *A. rubens* that provide novel insights into neuropeptide evolution at the superphylum/phylum level. Predicted signal peptides are shown in blue, putative neuropeptides are shown in red (with cysteine (C) residues underlined), C-terminal glycine (G) residues that are putative substrates for amidation are shown in orange and putative dibasic/tribasic cleavage sites are shown in green.

The sequences of *A. rubens* precursor proteins or the putative neuropeptides/polypeptide hormones derived from them were aligned with homologous proteins/peptides in other bilaterian species, some of which were identified here for the first time. Alignments were generated and edited using Jalview [47] and MAFFT [48] with JABAWS web service [49], employing default settings (gap opening penalty at local pairwise alignment = −2, similarity matrix = Blosum62, gap open penalty = 1.53, group size = 20, group-to-group gap extension penalty = 0.123). GeneDoc (http://genedoc.software.informer.com) was used to annotate the alignments and prepare alignment figures.

## 3. Results and discussion

By analysing *A. rubens* nerve cord transcriptome sequence data, we have identified 40 candidate neuropeptide precursors, which for the purposes of discussion we have divided into four groups. First, and most interestingly, precursors of neuropeptides that are the first members of neuropeptide families to be identified in a non-chordate species. Second, precursors of neuropeptides that are the first echinoderm/ambulacrarian representatives of bilaterian neuropeptide families to be identified. Third, precursors of neuropeptides that are homologues of neuropeptides that have been identified previously in other echinoderm species and that are members of bilaterian neuropeptide families. Lastly, precursors of putative neuropeptides that have, as yet, not been identified as homologues of neuropeptides in non-echinoderm animals.

### 3.1. Discovery of starfish neuropeptide precursors that provide new insights into neuropeptide evolution at the superphylum level

#### 3.1.1. Precursor of two kisspeptin-type peptides (ArKPP)

**Figure 3.**
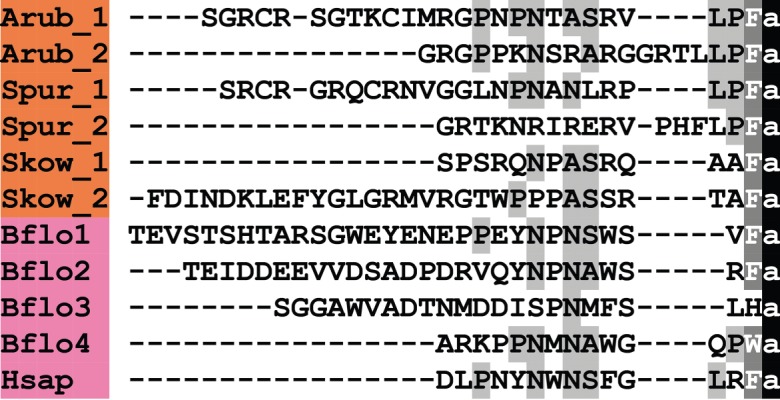
Alignment of ArKP1 and ArKP2 with other kisspeptin (KP)-type peptides. Accession numbers for the corresponding precursor proteins are: Arub, *A. rubens* KP-type precursor [GenBank: KT601705]; Spur, *S. purpuratus* KP-type precursor [GI:374768013]; Skow, *S. kowalevskii* KP-type precursor [GI:187123982]; Bflo1, *B. floridae* KP-type precursor 1 [GI:260826607]; Bflo2, *B. floridae* KP-type precursor 2 [GI:260827077]; Bflo3, *B. floridae* KP-type precursor 3 [GI:260826605]; Bflo4, *B. floridae* KP-type precursor 4 [GI:260793233]; Hsap, *Homo sapiens* KiSS-1 metastasis-suppressor precursor [GI:21950713].

A kisspeptin (KP)-type neuropeptide precursor in *A. rubens* (ArKPP) was identified as a 149-residue protein comprising a predicted 24-residue N-terminal signal peptide and two putative KP-type peptides—ArKP1 and ArKP2 (figure 2*a*; GenBank: KT601705). In common with human KP, ArKP1 has a C-terminal NxxSxxLxF-NH_2_ motif, but unlike human KP, ArKP1 has two cysteine residues in its N-terminal region, which may form a disulfide bridge. ArKP2 is similar to ArKP1 but it lacks the N-terminal pair of cysteine residues present in ArKP1 and it has additional residues in its C-terminal region. Discovery of ArKPP is important because it is the first KP-type precursor to be identified in a non-chordate species, consistent with the occurrence of KP-type receptors in non-chordates [5,6]. Furthermore, our discovery of ArKPP facilitated identification of KP-type precursors in other non-chordate deuterostomes, including the sea urchin *S. purpuratus* (phylum Echinodermata) and the acorn worm *Saccoglossus kowalevskii* (phylum Hemichordata). In figure 3, putative KP-type peptides in these two species are aligned with ArKP1 and ArKP2, human KP and four KP-type peptides that have been identified previously in the cephalochordate *Branchiostoma floridae* [5,50]. As in *A. rubens*, one of the KP-type peptides in *S. purpuratus* has two cysteine residues, but this feature is not present in KP-type peptides in non-echinoderm species. Therefore, the presence of a putative N-terminal disulfide bridge may be a unique characteristic of KP-type peptides in echinoderms.

KP or kiss1 was originally discovered in humans as a metastasis-suppressor gene [51,52], but subsequently it was found to have an important role in neuroendocrine control of reproductive maturation in humans and other vertebrates [53]. The key evidence for this was provided by the discovery that mutations in the KP receptor (GPR54) cause delayed puberty in humans [54,55], and the same phenotype was observed in GPR54-knockout mice [54,56] and KP-knockout mice [57,58]. KPs trigger hypothalamic secretion of GnRH, which then stimulates release of gonadotropins from the pituitary [59]. KP regulates the activity of GnRH neurons both directly [60] and indirectly [61,62], and also acts directly on gonadotropes [63]. Similarly, non-mammalian vertebrate KP-type peptides have been implicated in the regulation of reproductive function in several fish species [53,64,65].

At present nothing is known about the physiological roles of KP-type peptides in invertebrates. Our discovery of a KP-type precursor in starfish and other ambulacrarians, as reported here, provides a basis to address this issue for the first time.

#### 3.1.2. Precursor of a melanin-concentrating hormone-type peptide (ArMCHP)

**Figure 4.**
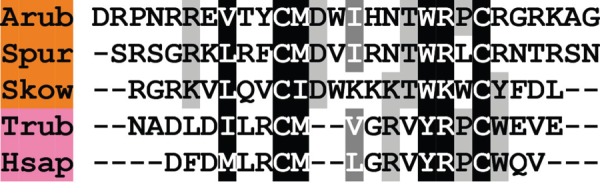
Alignment of ArMCH with other melanin-concentration hormone (MCH)-type peptides. Accession numbers for the corresponding precursor proteins are: Arub, *A. rubens* MCH-type precursor [GenBank: KT601706]; Spur, *S. purpuratus* MCH-type precursor [GI:109402760]; Skow, *S. kowalevskii* MCH-type precursor [GI:187231810]; Trub, *Takifugu rubripes* MCH precursor [GI:410918650]; Hsap, *H. sapiens* MCH precursor [GI:187445].

A melanin-concentrating hormone (MCH)-type neuropeptide precursor in *A. rubens* (ArMCHP) was identified as an 88-residue protein comprising a predicted 24-residue N-terminal signal peptide and a C-terminal 28-residue MCH-type peptide with two cysteine residues, which is preceded by a putative dibasic cleavage site (figure 2*b*; GenBank: KT601706). ArMCHP was identified on account of its sequence similarity with Spnp14, a putative neuropeptide precursor in the sea urchin *S. purpuratus* [9]. However, comparison of ArMCHP with vertebrate neuropeptides revealed sequence similarity with MCH-type peptides, as illustrated in figure 4. Furthermore, the location of the putative neuropeptide ArMCH in the C-terminal region of ArMCHP is likewise a characteristic of MCH-type precursors in vertebrates, providing further evidence of orthology [66]. Interestingly, identification of ArMCHP also facilitated identification of a MCH-type precursor in a hemichordate species, the acorn worm *S. kowalevskii* (figure 4).

Our discovery of MCH-type peptides in echinoderms and hemichordates is important because these are the first MCH-type neuropeptides to be discovered in invertebrates. Alignment of the invertebrate and vertebrate MCH-type peptides reveals a conserved pair of cysteine residues. These residues form a disulfide bridge in vertebrate MCH-type peptides [67] and therefore it is likely that invertebrate MCH-type peptides also have a disulfide bridge. Other conserved features include a methionine (or isoleucine) residue following the first cysteine residue and a basic amino acid (lysine or arginine) penultimate to the second cysteine residue. Interestingly, the number of residues that separate the two cysteine residues is greater in the invertebrate MCH-type peptides than in vertebrate MCH-type peptides, with two additional residues (DW or DV) located after the conserved methionine/isoleucine residue.

MCH was first identified in teleost fish on account of its effect in triggering a change in body colour [68,69]. Subsequently, MCH-type peptides were identified throughout the vertebrates [70–72], and experimental studies have revealed a wide range of physiological roles, including regulation of feeding, sleep and reproduction [73,74]. Our discovery of MCH-type peptides in ambulacrarians provides a unique opportunity to investigate for the first time the actions of these peptides in invertebrates and the evolution of the physiological roles of this family of neuropeptides.

### 3.2. Discovery of the first ambulacrarian/echinoderm representatives of bilaterian neuropeptide families

#### 3.2.1. Precursor of two tachykinin-type peptides (ArTKP)

**Figure 5.**
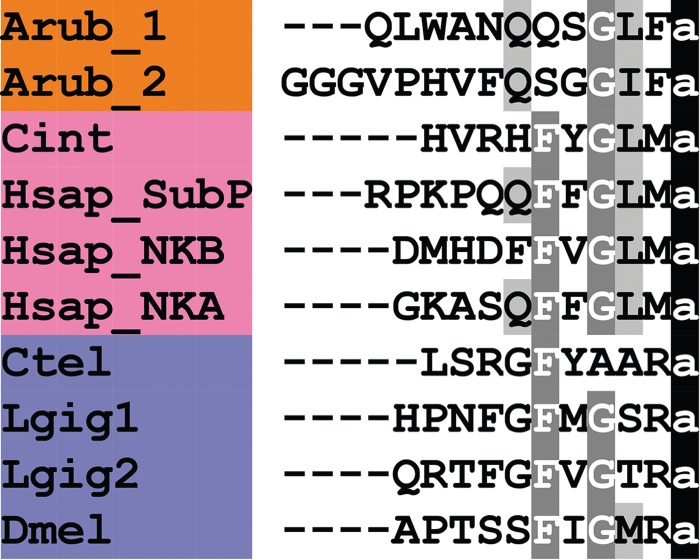
Alignment of ArTK1 and ArTK2 with other tachykinin (TK)-type peptides. Accession numbers for the corresponding precursor proteins are: Arub, *A. rubens* TK-type precursor [GenBank: KT601707]; Spur, *S. purpuratus* TK-type precursor [GI:109402899]; Cint, *C. intestinalis* TK-type precursor [GI:74136064]; Hsap_SubP, *H. sapiens* *β*-prepro TK precursor [GI:29482]; Hsap_NKB, *H. sapiens* neurokinin-*β* precursor [GI:48146502]; Hsap_NKA, *H. sapiens* TK4 precursor [GI:117938255]; Ctel, *Capitella teleta* [GI:161289578]; Lgig1, *Lottia gigantea* TK-type precursor 1 [GI:676441944]; Lgig2, *L. gigantea* TK-type precursor 2 [GI:163525452]; Dmel, *D. melanogaster* TK precursor [GI:442618676].

A tachykinin (TK)-type neuropeptide precursor in *A. rubens* (ArTKP) was identified as a 199-residue protein comprising a predicted 31-residue N-terminal signal peptide and two putative TK-type neuropeptides, ArTK1 and ArTK2, which are bounded by putative monobasic or dibasic cleavage sites (figure 2*c*; GenBank: KT601707). The presence of C-terminal glycine residues is indicative of post-translational conversion to amide groups in the mature peptides, and the presence of an N-terminal glutamine residue in ArTK1 is indicative of potential post-translational conversion to a pyroglutamate residue. ArTKP was identified because it has the characteristics of a neuropeptide precursor, and comparison of its sequence with bilaterian neuropeptide precursors revealed similarity with TK-type precursors. In particular, alignment of ArTK1 and ArTK2 with TK-type peptides in chordates reveals a conserved C-terminal GLXamide motif (figure 5).

TK-type peptides are a family of neuropeptides with a widespread phylogenetic distribution indicative of an ancestral bilaterian origin [5,6]. ArTK1 and ArTK2 are the first members of the TK-type neuropeptide family to be identified in an echinoderm and, more broadly, an ambulacrarian. Vertebrate TK-type peptides share the conserved C-terminal pentapeptide motif FxGLM-NH_2_, whereas TK-type peptides in protostomian invertebrates typically share the conserved C-terminal pentapeptide motif FxGxR-NH_2_ (figure 5). ArTK1 and ArTK2 have the C-terminal pentapeptide motifs QSGLF-NH_2_ and QSGGIF-NH_2_, respectively, which share the common motif GxF-NH_2_, with x representing a hydrophobic leucine or isoleucine residue, and in this respect ArTK1 and ArTK2 are similar to vertebrate TK-type peptides (figure 5). Conversely, a conserved feature of TK-type peptides that is not present in the starfish peptides is a phenylalanine residue at the fifth position from the C-terminal amide.

The first TK-type peptide to be discovered was the mammalian neuropeptide substance P (SP) [75–77]. Subsequently, two other TKs were discovered in mammals—neurokinin A (NKA) and neurokinin B (NKB) [78–80]. TK-type peptides act as neurotransmitters, neuromodulators and neurohormones in both the central and peripheral nervous system of mammals, with roles in regulation of, for example, intestinal motility [81], smooth muscle contraction [82] and cardiovascular function [83]. TK-type peptides have also been identified in non-mammalian vertebrates [84], in urochordates [85] and in protostomian invertebrates, including molluscs [86,87], annelids [88,89], arthropods [90–93] and nematodes [94]. Investigation of the physiological roles of TK-type peptides in protostomes has revealed, for example, effects on gut/oviduct motility and lipid synthesis in insects and rhythmic motor output of the somatogastric system in crustaceans [90,95–97]. Now with the discovery of ArTK1 and ArTK2 in *A. rubens*, as reported here, an opportunity to investigate for the first time the physiological roles of TK-type neuropeptides in an echinoderm has been provided.

#### 3.2.2. Precursor of a somatostatin-type peptide (ArSSP)

**Figure 6.**
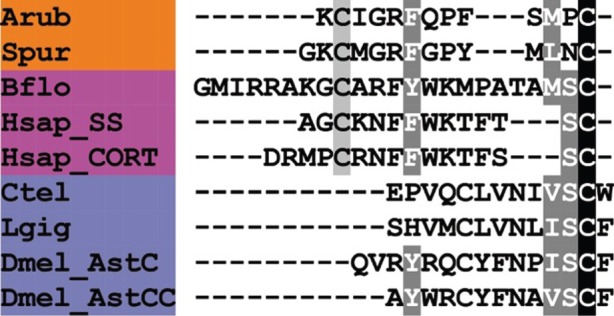
Alignment of ArSS with other somatostatin (SS)-type peptides and AST-C-type peptides. Accession numbers for the corresponding precursor proteins are: Arub, *A. rubens* SS-type precursor [GenBank: KT601708]; Spur, *S. purpuratus* SS-type precursor [GI:390344260]; Bflo, *B. floridae* SS-type precursor [JGI:72051]; Hsap_SS, *H. sapiens* SS precursor [GI:21619155]; Hsap_CORT, *H. sapiens* cortistatin (CORT) precursor [GI:110645815]; Ctel, *C. teleta* AST-C-type precursor [GI:161295377]; Lgig, *L. gigantea* AST-C-type precursor [GI:163505903]; Dmel_AstC, *D. melanogaster* AST-C-type precursor [GI:665407583]; Dmel_AstCC, *D. melanogaster* AST-CC-type precursor [GI:665407585].

A somatostatin (SS)-type neuropeptide precursor in *A. rubens* (ArSSP) was identified as a 132-residue protein comprising a predicted 24-residue N-terminal signal peptide and a 13-residue SS-type peptide that is preceded N-terminally by a putative dibasic cleavage site (figure 2*d*; GenBank: KT601708). ArSSP was identified based on its sequence similarity with Spnp19, a putative neuropeptide precursor previously identified in the sea urchin *S. purpuratus* [9]. However, comparison of ArSSP and Spnp19 (SpSSP) with known neuropeptide precursors revealed similarity with vertebrate SS/cortistatin-type precursors. For example, both ArSS and SpSS share a CxxxFxxxxxxC motif with human SS and cortistatin (figure 6). In vertebrate SS/cortistatin-type peptides, the two cysteine residues form an intramolecular disulfide bridge [98], and therefore it is likely that the same post-translational modification occurs in the starfish and sea urchin SS-type peptides. Furthermore, as with ArMCHP, another feature of ArSSP that suggests homology with vertebrate SS-type precursors is the conserved location of an SS-type neuropeptide at the C-terminus of the precursor [99]. The discovery of SS-type neuropeptides in starfish and sea urchins is important because these are the first to be identified in echinoderms and they join a bilaterian family of neuropeptides that include allatostatins in arthropods [5,6,100].

SS was first isolated from sheep hypothalamus [101], and was initially characterized as a neuroendocrine peptide that inhibits release of pituitary hormones such as growth hormone and prolactin [102,103]. Subsequently, an SS-type peptide termed cortistatin was discovered in humans [104] and has since been found to occur throughout the tetrapod vertebrates [99]. Additional SS-type peptides are present in teleost fish [105], and a candidate SS-type peptide was recently identified in the cephalochordate *B. floridae* (figure 6) [5]*.* In addition to its effects on pituitary hormone release, SS also has central actions that influence motor activity, sensory processing and cognition [106].

Allatostatins inhibit juvenile hormone (JH) biosynthesis in the *corpora allata* of insects and three structurally unrelated types of allatostatins have been identified. Allatostatins were first isolated from the cockroach *Diploptera punctata* and these are now referred to as allatostatin-A [107–109], while neuropeptides with allatostatic activity that were originally identified from the cricket *Gryllus bimaculatus* are referred to as allatostatin-B [110]. The allatostatin-C (AST-C)-type peptides that are related to vertebrate SSs were first identified in the tobacco hornworm *Manduca sexta* [111], but have subsequently been identified in a number of arthropods, including numerous insect species [100,112–115].

Our discovery of precursors of SS-type neuropeptides in echinoderms is important because it provides a basis for investigation of their physiological roles in non-chordate deuterostomes. A common theme that emerges from comparison of the actions of SS-type and AST-C-type neuropeptides in vertebrates and insects, respectively, is their roles in inhibitory regulation of the biosynthesis/release of hormones that regulate development and growth. Against this background, it will be of great interest to discover the physiological roles of SS-type neuropeptides in echinoderms.

#### 3.2.3. Precursor of two pigment-dispersing factor-type peptides (ArPDFP)

**Figure 7.**
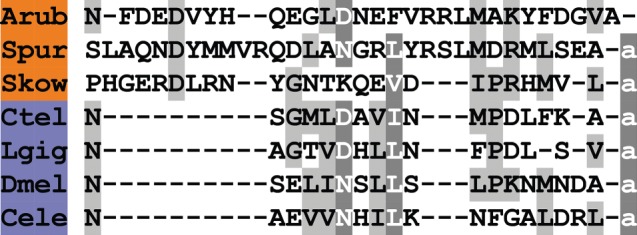
Alignment of ArPDF1 and ArPDF2 with other pigment-dispersing factor (PDF)-type peptides. Accession numbers for the corresponding precursor proteins are: Arub, *A. rubens* PDF-type precursor [GenBank: KT601709]; Spur, *S. purpuratus* PDF-type precursor [GI:115899431]; Skow, *S. kowalevskii* PDF-type precursor [GI:187067819]; Ctel, *C. teleta* PDF-type precursor [JGI:204689]; Lgig, *L. gigantea* cerebrin precursor [GI:676458325]; Dmel, *D. melanogaster* PDF precursor [GI:281362639]; Cele, *C. elegans* PDF precursor [GI:25149644].

A pigment-dispersing factor (PDF)-type neuropeptide precursor in *A. rubens* (ArPDFP) was identified as a 104-residue protein comprising a predicted 22-residue N-terminal signal peptide and two putative PDF-type neuropeptides bounded by dibasic/tribasic cleavage sites: ArPDF1, a 35-residue polypeptide with a C-terminal glycine residue that may be a substrate for amidation, and ArPDF2, a 29-residue polypeptide (figure 2*e*; GenBank: KT601709). ArPDFP was identified on account of its sequence similarity with a protein in the sea urchin *S. purpuratus*, which was reported previously as a corticotropin-releasing hormone (CRH)-type neuropeptide precursor [6]. However, three observations lead us to conclude that ArPDFP is, as its name implies, a PDF-type precursor. First, the *A. rubens* and *S. purpuratus* PDF-type peptides share sequence similarity with a PDF-type peptide that was identified recently in the hemichordate *S. kowalevskii* [5] and with PDF/cerebrin-type peptides in protostomian invertebrates, as illustrated in figure 7. Second, the occurrence of two putative neuropeptides in ArPDFP is a feature that is also seen other PDF-type precursors [116] but not in CRH-type precursors. Third, we have identified other neuropeptide precursors in *A. rubens* and the sea urchin *S. purpuratus* that exhibit a higher level of similarity with CRH-type precursors (see below).

PDF or pigment-dispersing hormone was first identified in crustacean species on account of its effect in causing pigment migration in retinal pigment cells of the eyes [117]. Subsequently, PDF-type peptides were identified in other arthropods [118], and experimental studies on *Drosophila* revealed that PDF released by sub-populations of neurons in the brain is required for normal circadian patterns of locomotor activity [119–121]. PDF-type peptides have also been identified in nematodes [122] and lophotrochozoans, including molluscs [7] and annelids [8]. PDF-type neuropeptide signalling in the nematode *C. elegans* regulates locomotor activity and egg-laying [123], while a PDF-type neuropeptide in the mollusc *Aplysia californica* (‘cerebrin’) affects the feeding motor pattern, mimicking the motor-pattern alterations observed in food-induced arousal states [124].

Phylogenetic studies indicate that PDF-type peptides are a bilaterian neuropeptide family that has been lost in the chordate lineage [5,6]. Therefore, the discovery of PDF-type neuropeptides in echinoderms, as reported here, and in hemichordates [5] is of particular interest because it provides a unique opportunity for the first investigations of the physiological roles of this family of neuropeptides in deuterostomian invertebrates.

#### 3.2.4. Precursor of a corticotropin-releasing hormone-type peptide (ArCRHP)

**Figure 8.**
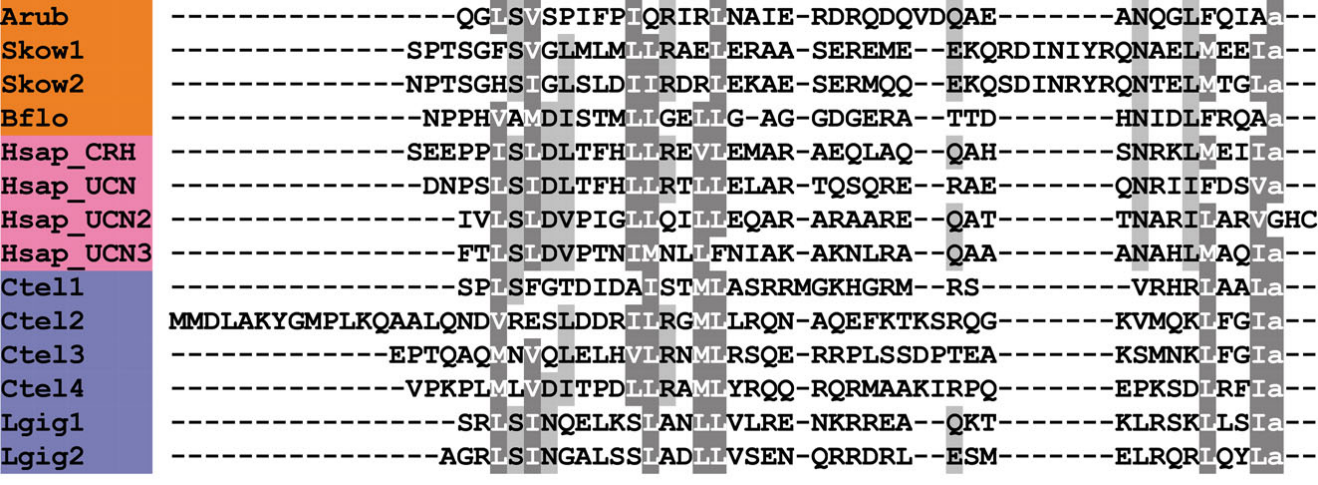
Alignment of ArCRH with other corticotropin-releasing hormone (CRH)-type peptides. Accession numbers for the corresponding precursor proteins are: Arub, *A. rubens* CRH-type precursor [GenBank: KT601710]; Skow1, *S. kowalevskii* CRH-type precursor 1 [GI:281433636]; Skow2, *S. kowalevskii* CRH-type precursor 2 [GI:281433636]; Bflo, *B. floridae* CRH-type precursor [GI:260786674]; Hsap_CRH, *H. sapiens* CRH precursor [GI:30583744]; Hsap_UCN, *H. sapiens* urocortin (UCN) precursor [GI:49457481]; Hsap_UCN2, *H. sapiens* stresscopin-related protein precursor [GI:14029393]; Hsap_UCN3, *H. sapiens* stresscopin precursor [GI:15026913]; Ctel1, *C. teleta* CRH-type precursor 1 [GI:161303031]; Ctel2, *C. teleta* CRH-type precursor 2 [JGI:190906]; Ctel3, *C. teleta* CRH-type precursor 3 [JGI:190906]; Ctel4, *C. teleta* CRH-type precursor 4 [JGI:194553]; Lgig1, *L. gigantea* CRH-type precursor 1 [GI:676493124]; Lgig2, *L. gigantea* CRH-type precursor 2 [GI:163524672].

A CRH-type neuropeptide precursor in *A. rubens* (ArCRHP) was identified as a 130-residue protein comprising a predicted 28-residue N-terminal signal peptide and a 41-residue CRH-type peptide sequence bounded by dibasic/tribasic cleavage sites (figure 2*f*; GenBank: KT601710). An N-terminal glutamine residue and a C-terminal glycine residue may be substrates for post-translational modifications that give rise to an N-terminal pyroglutamate residue and a C-terminal amide group in the mature peptide. As highlighted above, neuropeptides in echinoderms purported to be CRH-type peptides have been reported previously [5,6,10], but further analysis here has revealed that these are in fact PDF-type peptides. Therefore, ArCRHP is the first *bone fide* CRH-type precursor to be identified in an echinoderm. Previous studies have identified CRH-type precursors in other deuterostomian invertebrates, including the hemichordate *S. kowalevskii* [5,6] and the cephalochordate *B. floridae* [5], and in figure 8 we show an alignment of the *A. rubens* CRH-type peptide (ArCRH) with homologues from these two species, human CRH/urocortin-type peptides and CRH-type peptides in lophotrochozoan protostomes. The alignment highlights several residues that are conserved at the interphyletic level.

CRH was first identified as a hypothalamic neurohormone that stimulates release of adrenocorticotropic hormone in response to stress in mammals [125,126]. CRH-type peptides have also been identified in non-mammalian vertebrates, and in addition to its corticotropic effect, CRH acts as a thyrotropic hormone [127]. A CRH-type peptide in insects, DH44, acts as a diuretic hormone, stimulating fluid secretion by Malpighian tubules by elevating cAMP levels [128]. In the mollusc *Aplysia*, egg-laying hormone (ELH) is a CRH-type peptide [129,130] and ELH/CRH-type peptides have subsequently been identified in other molluscan species [131]. It has also been reported that ELH-type peptides trigger gamete release in annelids [132]. Against this backdrop of diverse physiological roles, the discovery of CRH-type peptides in starfish and other deuterostomian invertebrates provides a unique opportunity to obtain new insights into the evolution of the physiological roles of CRH-type neuropeptides in the animal kingdom.

### 3.3. Discovery of novel starfish representatives of bilaterian neuropeptide and peptide hormone families

#### 3.3.1. Precursor of a vasopressin/oxytocin-type neuropeptide (asterotocin)

**Figure 9.**
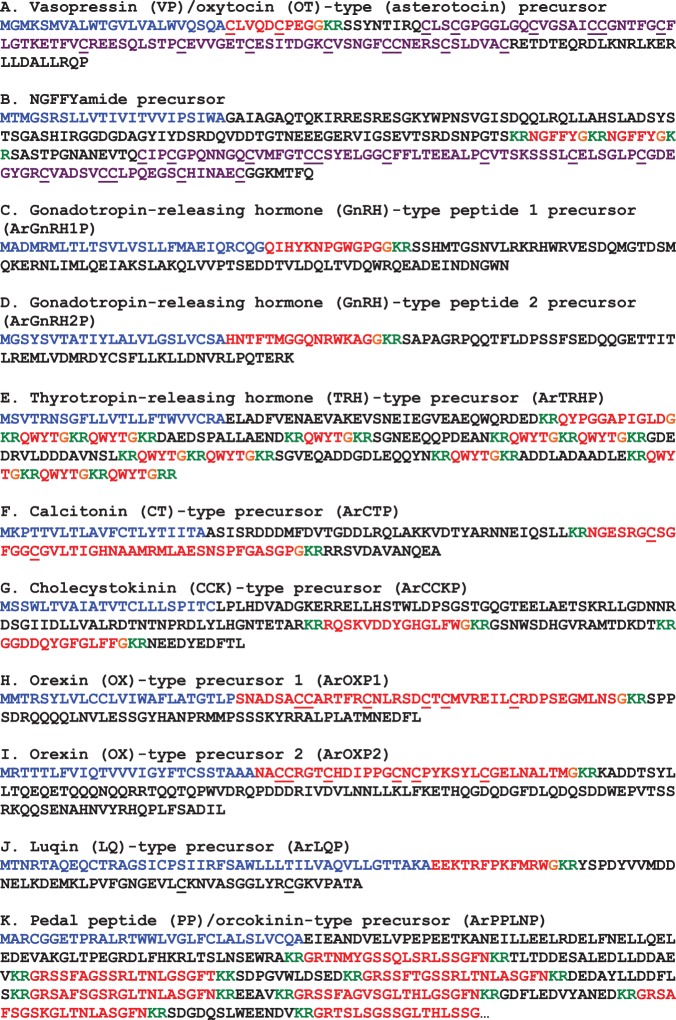
Precursors of neuropeptides in *A. rubens* that are novel echinoderm representatives of bilaterian neuropeptide families. Predicted signal peptides are shown in blue, putative neuropeptides are shown in red (with cysteine (C) residues underlined), C-terminal glycine (G) residues that are putative substrates for amidation are shown in orange and putative dibasic cleavage sites are shown in green. For the (*a*) asterotocin and (*b*) NGFFYamide precursors, the C-terminal neurophysin domain (with the characteristic 14 cysteine (*c*) residues underlined) is shown in purple.

A VP/OT-type neuropeptide precursor in *A. rubens* was identified as a 147-residue protein comprising a predicted 23-residue N-terminal signal peptide, a VP/OT-type neuropeptide sequence (CLVQDCPEGG) followed by a dibasic cleavage site and a neurophysin domain (figure 9*a*; GenBank: KT601711). This structure of the precursor conforms to the evolutionarily conserved organization of VP/OT-type neuropeptide precursors throughout the Bilateria [5,133]. Mature VP/OT-type neuropeptides are typically C-terminally amidated and have a disulfide bridge between two highly conserved cysteine residues, which are crucial for biological activity [134–136]. Therefore, the predicted neuropeptide product of the VP/OT-type precursor in *A. rubens* is CLVQDCPEG-NH_2_, with a disulfide bridge between the two cysteine residues. We refer to this putative starfish VP/OT-type neuropeptide as ‘asterotocin’, in keeping with the naming of a VP/OT-type peptide, ‘echinotocin’, which was identified previously in the echinoid (sea urchin) *S. purpuratus* [17].

**Figure 10.**
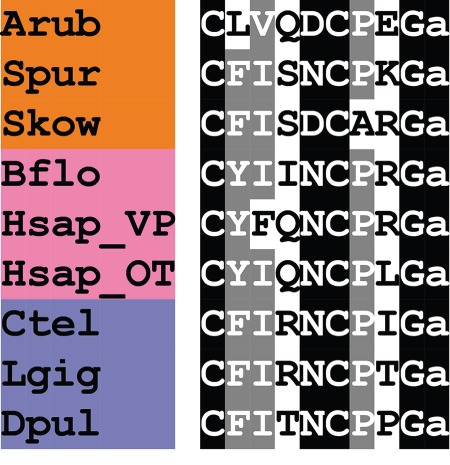
Alignment of asterotocin with other vasopressin/oxytocin (VP/OT)-type peptides. Accession numbers for the corresponding precursor proteins are: Arub, *A. rubens* asterotocin precursor [GenBank: KT601711], Spur, *S. purpuratus* echinotocin precursor [GI:390337108]; Skow, *S. kowalevskii* VP/OT-type precursor [GI:187155721]; Bflo, *B. floridae* VP/OT-type precursor [GI:260828088]; Hsap_VP, *H. sapiens* VP precursor [GI:340298]; Hsap_OT, *H. sapiens* OT precursor [GI:189410]; Ctel, *C. teleta* VP/OT-type precursor [JGI:173251]; Lgig, *L. gigantea* VP/OT-type precursor [JGI:53893]; Dpul, *Daphnia pulex* VP/OT-type precursor [JGI:59567].

In figure 10, we have compared the sequence of asterotocin with VP/OT-type neuropeptides that have been identified in other animals. The presence in asterotocin of leucine and valine residues at positions 2 and 3, respectively, is atypical for VP/OT-type peptides but consistent with the occurrence of hydrophobic residues at these positions in other VP/OT-type peptides. The aspartic acid at position 5 in asterotocin is also atypical (more commonly it is an asparagine), but this feature is also seen in several other VP/OT-type peptides, including peptides identified in the hemichordate *S. kowalevskii*, the urochordates *Ciona intestinalis* and *Styela plicata* [137,138], and the nematode *C. elegans* [139]. The most unusual and interesting structural characteristic of asterotocin is the presence of a glutamate residue at position 8 because, to the best of our knowledge, this is the first of example of a VP/OT-type neuropeptide with an acidic residue in this position. Typically, the residue in this position is a basic residue (e.g. lysine or arginine in mammalian VPs) or a hydrophobic residue (e.g. leucine in OT). Furthermore, this feature of asterotocin may be unique to starfish or a sub-set of echinoderms because the VP/OT-type neuropeptide previously identified in the sea urchin *S. purpuratus* (echinotocin; CFISNCPKG-NH_2_) has a lysine residue at position 8 [17]. Therefore, it may be of interest to investigate the importance of the chemical characteristics of the amino acid at position 8 for the biological activity of asterotocin.

VP was first discovered in mammals on account of its effects as a regulator of blood pressure and diuresis [140,141], while OT was discovered on account of its effects as a stimulator of uterine contraction and lactation [142]. However, in addition to these peripheral hormonal functions, VP and OT also have central neuromodulatory roles in social cognition and behaviour, including mother–infant bonding [143], and pair bonding and attachment [144,145]. Currently, there is great interest in both VP and OT with respect to understanding human social behaviour and neuropathology, with implications of involvement in disorders such as autism, social anxiety disorder, borderline personality disorder and schizophrenia [146,147].

As illustrated in figure 10, VP/OT-type neuropeptides have a widespread phylogenetic distribution indicative of an evolutionary origin dating back to the common ancestor of bilaterians [5,6]. VP/OT-type peptides have been identified in many vertebrate species [148,149] and in deuterostomian invertebrates, including the urochordates [137,138], the cephalochordate *B. floridae* [150], the hemichordate *S. kowalevskii* [151] and the echinoderm *S. purpuratus* [17]. VP/OT-type peptides have also been identified in protostomes [152–154]. Furthermore, recent studies on the nematode *C. elegans* have shown that the VP/OT-type peptide nematocin [139,155] is involved in gustatory associative learning and control of mating behaviour in this species [1,139]. It has therefore been hypothesized that VP/OT-type peptides may be ancient modulators of reproductive behaviour and associative learning [1].

Currently, little is known about the physiological roles of VP/OT-type neuropeptides in echinoderms. *In vitro* pharmacological tests have, however, revealed that echinotocin causes contraction of tube foot and oesophagus preparations in the sea urchin species *Echinus esculentus* [17]. These effects of echinotocin are consistent with contractile actions of VP and OT on blood vessels and uterus, respectively, in mammals [140–142]. Likewise, a VP/OT-type neuropeptide causes contraction of the inhalant and exhalant siphons in the urochordate *S. plicata* [138]. With the discovery of the asterotocin precursor in *A. rubens*, as reported here, an opportunity to investigate the physiological roles of a VP/OT-type neuropeptide in starfish has been provided.

#### 3.3.2. Precursor of NGFFYamide, a neuropeptide-S/NG peptide/crustacean cardioactive peptide-type neuropeptide

Discovery of the *A. rubens* NGFFYamide precursor was reported recently [156] and was accomplished by analysis of the same neural transcriptome dataset analysed here; therefore, it is included here for sake of completeness. The NGFFYamide precursor is a 239-residue protein comprising a predicted 23-residue N-terminal signal peptide, two copies of the sequence NGFFYG bounded by dibasic cleavage sites and a C-terminal neurophysin domain containing 14 cysteine residues (figure 9*b*; GenBank: KC977457), which is a conserved feature of neurophysins. Post-translational conversion of the C-terminal glycine residue of the NGFFYG peptide to an amide group has been confirmed by mass spectrometry [156].

NGFFYamide belongs to a bilaterian family of neuropeptides that include the vertebrate peptide neuropeptide-S (NPS), protostomian crustacean cardioactive peptide (CCAP)-type neuropeptides and NG peptides, neurophysin-associated peptides in deuterostomian invertebrates that are characterized by an Asn-Gly (NG) motif, which include NGFFYamide. This relationship of NG peptides with NPS/CCAP-type peptides was proved recently with the discovery that the NG peptide NGFFFamide is the ligand for an NPS/CCAP-type receptor in the sea urchin *S. purpuratus* [157]. Furthermore, the presence of a neurophysin domain in NG peptide precursors reflects a common ancestry with VP/OT-type precursors, with gene duplication in a common ancestor of the Bilateria having given rise to the VP/OT-type and the NPS/NG peptide/CCAP-type neuropeptide signalling systems [5,157].

NPS acts as an anxiolytic in mammals and induces wakefulness and hyperactivity [158], whereas CCAP activates the ecdysis motor programme in arthropods that results in shedding of the exoskeleton [159,160]. Thus, a common theme for these neuropeptides appears to be roles in behavioural states associated with a heightened state of arousal [157]. We have investigated the physiological roles of NGFFYamide in *A. rubens* and have discovered that it potently stimulates contraction of the cardiac stomach *in vitro* [156]. Starfish feed by everting their cardiac stomach over the digestible parts of prey such as mussels and *in vivo* pharmacological tests have revealed that NGFFYamide triggers retraction of the everted cardiac stomach [156]. Therefore, it is likely that NGFFY amide acts physiologically to mediate neural control of cardiac stomach retraction in starfish.

#### 3.3.3. Precursor of gonadotropin-releasing hormone-type peptide 1 (ArGnRH1P)

A GnRH-type neuropeptide precursor in *A. rubens* (ArGnRH1P) was identified as a 121-residue protein comprising a predicted 27-residue N-terminal signal peptide and a GnRH-type peptide sequence (QIHYKNPGWGPG) followed by a dibasic cleavage site (figure 9*c*; GenBank: KT601712). The presence of an N-terminal glutamine residue and a C-terminal glycine residue are indicative of post-translational modifications giving rise to an N-terminal pyroglutamate residue and a C-terminal amide group in the putative mature peptide (pQIHYKNPGWGPG-NH_2_; ArGnRH1), which would be consistent with GnRH-type neuropeptides that have been identified in other species [161].

**Figure 11.**
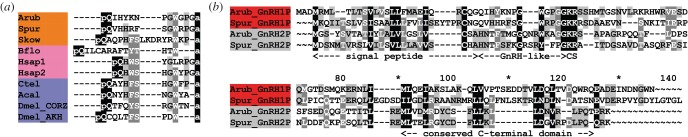
Alignment of *A. rubens* GnRH-type peptides/precursors with other gonadotropin-releasing hormone (GnRH)-type peptides/precursors. (*a*) Alignment of GnRH-type peptides. Accession numbers for the corresponding precursor proteins are: Arub, *A. rubens* GnRH-type precursor 1 [GenBank: KT601712]; Spur, *S. purpuratus* GnRH-type precursor 1 [GI:390361802]; Skow, *S. kowalevskii* GnRH-type precursor [GI:585702722]; Bflo, *B. floridae* GnRH-type precursor [GI:568818760]; Hsap1, *H. sapiens* GnRH precursor 1 [GI:133908609]; Hsap2, *H. sapiens* GnRH precursor 2 [GI:109731929]; Ctel, *C. teleta* GnRH-type precursor [GI:161294493]; Acal, *A. californica* GnRH-type precursor [GI:325296898]; Dmel_CORZ, *D. melanogaster* corazonin (CORZ) precursor [GI:386765761]; Dmel_AKH, *D. melanogaster* adipokinetic hormone (AKH) precursor [GI:281365621]. (*b*) Alignment of GnRH-type precursors. Accession numbers for the corresponding precursor proteins are: Arub_GnRH1P, *A. rubens* GnRH-type precursor 1 [GenBank: KT601712]; Spur_GnRH1P, *S. purpuratus* GnRH-type precursor 1 [GI:390361802]; Arub_GnRH2P, *A. rubens* GnRH-type precursor 2 [GenBank: KT601713]; Spur_GnRH2P, *S. purpuratus* GnRH-type precursor 2 [GI:109403263].

GnRH-type peptides have a widespread phylogenetic distribution indicative of an evolutionary origin dating back to the common ancestor of bilaterians [5,6]. The structural organization of ArGnRH1P conforms to other GnRH-type precursor proteins, with a single GnRH-type peptide located directly after the N-terminal signal peptide (figure 9*c*). Furthermore, comparison of the sequence of ArGnRH1 with other GnRH-type peptides reveals several conserved features, including the aforementioned predicted N-terminal pyroglutamate and C-terminal amide as well as a GWxP motif at positions 8–11 in ArGnRH1 (figure 11*a*). The C-terminal PG motif in ArGnRH1 is a feature that it shares with human GnRHs.

GnRH was first discovered in mammals as a reproductive regulator through its stimulatory effect on the release of the gonadotropins luteinizing hormone (LH) and follicle-stimulating hormone (FSH) from the anterior pituitary gland [162]. GnRH-type peptides have subsequently been identified in other vertebrates [163,164] and deuterostomian invertebrates, including urochordates [165,166], the cephalochordate *B. floridae* [167], the hemichordate *S. kowalevskii* [5] and the echinoderm *S. purpuratus* [9]. GnRH-type peptides have also been identified in lophotrochozoans, including several molluscan species [168–170] and annelids [8,161,169]. Interestingly, it has been discovered that adipokinetic hormone (AKH) in the ecdysozoans (arthropods and nematodes) and corazonin (CORZ)-type peptides and AKH/CORZ-related peptides (ACP) in the arthropods are homologues of GnRH [171–173]. In insects, AKH is synthesized in the corpora cardiaca, which are functionally equivalent to the pituitary gland in vertebrates, and acts to mobilize energy from the fat body during flight and locomotion [174,175]. AKH-type peptides in the nematode *C. elegans* regulate fertility, indicating that GnRH/AKH-type peptides have an evolutionarily ancient role in neural control of reproductive processes [171]. The physiological roles of GnRH-type peptides in echinoderms are currently unknown. Therefore, the discovery of ArGnRH1 in *A. rubens*, as reported here, provides an opportunity to address this issue.

#### 3.3.4. Precursor of gonadotropin-releasing hormone-type peptide 2 (ArGnRH2P)

A second GnRH-type neuropeptide precursor in *A. rubens* (ArGnRHP2) was identified as a 99-residue protein comprising a predicted 23-residue N-terminal signal peptide and a putative GnRH-type peptide sequence (HNTFTMGGQNRWKAGG) followed by a dibasic cleavage site (figure 9*d*; GenBank: KT601713). The presence of a C-terminal glycine residue is indicative of a post-translational modification that gives rise to a C-terminal amide group on the mature peptide (HNTFTMGGQNRWKAG-NH_2_). However, the absence of an N-terminal pyroglutamate residue is atypical for GnRH-type neuropeptides [161].

ArGnRHP2 was identified on account of its sequence similarity with Spnp12, a putative neuropeptide precursor previously identified in the sea urchin *S. purpuratus* [9]. However, here we have discovered that the structural organization and sequence of Spnp12 and its homologue in *A. rubens* are similar to GnRH-type precursors in *S. purpuratus*, *A. rubens* (figure 11*b*) and other species. Thus, the GnRH-type peptide is located directly following the signal peptide, and ArGnRH1P and ArGnRH2P have a conserved C-terminal domain. Furthermore, ArGnRH2 shares a C-terminal WxxG-NH_2_ motif with ArGnRH1 (figure 11*b*).

Investigation into the evolution of GnRH-type neuropeptide signalling systems in the Bilateria has revealed a complex picture [167]. A variety of neuropeptide types, including chordate GnRH-type peptides, arthropod AKH-type, CORZ and ACP-type peptides, appear to have evolved from a common ancestral peptide that occurred in the common ancestor of the Bilateria. However, the timing of the gene duplications that gave rise to this heterogeneous family of neuropeptides that occur in extant bilaterians remains unclear. Discovery of a second GnRH-type neuropeptide precursor in echinoderms (starfish and sea urchins) adds further complexity. However, our findings provide a basis for functional characterization of ArGnRH1 and ArGnRH2, which may provide new insights that facilitate a deeper understanding of the evolution of GnRH-type signalling systems in the Bilateria.

#### 3.3.5. Precursor of thyrotropin-releasing hormone (TRH)-type peptides (ArTRHP)

A TRH-type neuropeptide precursor in *A. rubens* (ArTRHP) was identified as a 225-residue protein comprising a predicted 23-residue N-terminal signal peptide and 12 putative TRH-type peptides bounded by dibasic cleavage sites (figure 9*e*; GenBank: KT601714). These include a single copy of the peptide sequence QYPGGAPIGLDG and 11 copies of the peptide sequence QWYTG. The presence of an N-terminal glutamine residue and a C-terminal glycine residue in these peptide sequences are indicative of potential post-translational modification to an N-terminal pyroglutamate and a C-terminal amide group in the mature peptides, which would be consistent with the structure of TRH in mammals (pQHP-NH_2_). Hence the predicted structure of the multi-copy TRH-type peptide in *A. rubens* is pQWYT-NH_2_. Furthermore, the occurrence of multiple copies of this peptide is consistent with the organization of TRH-type precursors in vertebrates, which comprise multiple copies of TRH [176,177].

**Figure 12.**
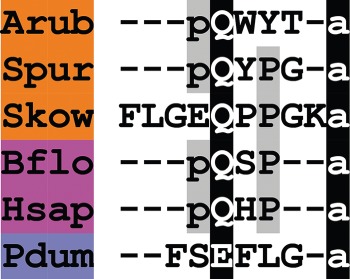
Alignment of ArTRH with other thyrotropin-releasing hormone (TRH)-type peptides. Accession numbers for the corresponding precursor proteins are: Arub, *A. rubens* TRH-type precursor [GenBank: KT601714]; Spur, *S. purpuratus* TRH-type precursor [GI:109402869]; Skow, *S. kowalevskii* TRH-type precursor [GI:187216047]; Bflo, *B. floridae* TRH-type precursor [GI:260784028]; Hsap, *H. sapiens* TRH precursor [GI:485464565]; Pdum, *P. dumerilii* EFLGamide precursor [GI:332167915].

Comparison of ArTRHP with TRH-type precursors that have been identified in other echinoderm species reveals similarities. TRH-type precursors in the sea urchin *S. purpuratus* [9] and in the sea cucumber *A. japonicus* [10] comprise 19 putative neuropeptides. The most abundant predicted neuropeptide product of the *S. purpuratus* precursor is pQYPG-NH_2_ and the most abundant predicted neuropeptide product of the *A. japonicus* precursor is pQYFA-NH_2_. Thus, with our discovery of the putative pQWYT-NH_2_ peptide in *A. rubens*, it appears that a tetrapeptide with an N-terminal pyroglutamate and a C-terminal amide are conserved features of TRH-type peptides in echinoderms, which contrasts with the tripeptidic TRH-type peptides that occur in chordates, namely pQHP-NH_2_ in vertebrates and pQSP-NH_2_ in the cephalochordate *B. floridae* (figure 12). Comparison of the sequences of the most abundant of the TRH-type peptides in the three echinoderm species reveals similarities, with the amino acids in positions 2 and 3 having side chains that are aromatic (Y, F or W) or cyclic (P) (figure 12). In this respect, there is similarity with TRH in vertebrates, which has an aromatic histidine residue in position 2 and a cyclic proline residue in position 3 (figure 12).

TRH was first identified in mammals as a hypothalamic peptide that stimulates the release of the hormones thyroid-stimulating hormone (TSH) and prolactin from the anterior pituitary gland. Release of TSH then triggers release of thyroid hormones (T_3_ and T_4_) that stimulate metabolism in cells throughout the body and promote growth and development [125]. TRH also acts as neurotransmitter or neuromodulator in other regions of the brain [178,179]. In amphibians and fish, TRH stimulates the release of pituitary growth hormone and prolactin but has little or no effect on the secretion of TSH [180]. Thus, the role of TRH as a regulator of TSH release in mammals does not apply to all vertebrate species.

The occurrence of TRH-type receptors in deuterostomes and protostomes indicates that the evolutionary origin of this neuropeptide signalling system dates back to the common ancestor of the Bilateria [5,6]. In support of this, FSEFLGamide has recently been discovered as the ligand for a TRH-type receptor in the annelid *Platynereis dumerilii* [181]. It has therefore been proposed that the ‘EFLGamides’ identified in the lophotrochozoans [182] are orthologous to deuterostomian TRH-type neuropeptides [181]. The characterization of the *P. dumerilii* TRH-type receptor highlights the importance of receptor orthology in determining relationships between neuropeptides in distantly related phyla that share modest sequence similarity. The discovery of TRH-type peptides in echinoderms [9,10], including the starfish *A. rubens* (this paper), and in the cephalochordate *B. floridae* [6] has provided opportunities to investigate for the first time the physiological roles of TRH-type peptides in deuterostomian invertebrates.

#### 3.3.6. Precursor of a calcitonin-type peptide (ArCTP)

A CT-type neuropeptide precursor in *A. rubens* (ArCTP) was identified as a 114-residue protein comprising a predicted 21-residue N-terminal signal peptide and a 40-residue CT-type peptide sequence bounded by dibasic cleavage sites (figure 9*f*; Gen Bank: KT601715). The presence of a C-terminal glycine residue is indicative of a post-translational modification that gives rise to a C-terminal amide group on the mature peptide, which would be consistent with CT-type neuropeptides that have been identified in other species. The putative CT-type peptide (ArCT) contains two cysteine residues in the N-terminal region, which may form an intramolecular disulfide bridge in accordance with other CT-type peptides [182,183].

**Figure 13.**
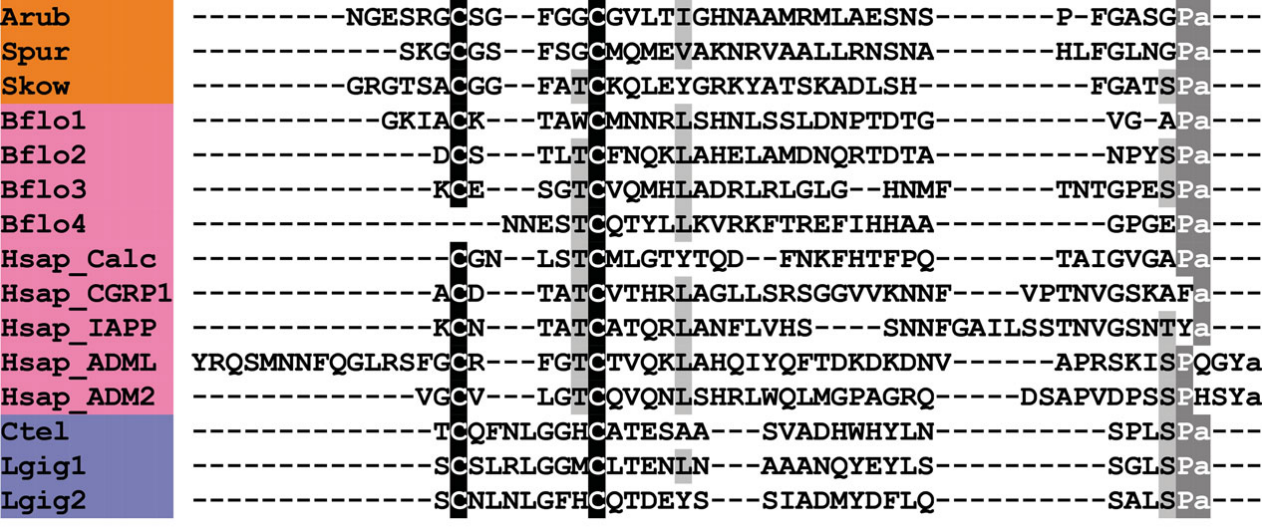
Alignment of ArCT with other calcitonin (CT)-type peptides. Accession numbers for the corresponding precursor proteins are: Arub, *A. rubens* CT-type precursor [GenBank: KT601715]; Spur, *S. purpuratus* CT-type precursor [GI:115767208]; Skow, *S. kowalevskii* CT-type precursor [GI:187217193]; Bflo1, *B. floridae* CT-type precursor 1 [GI:260826569]; Bflo2, *B. floridae* CT-type precursor 2 [GI:260826567]; Bflo3, *B. floridae* CT-type precursor 3 [GI:260826573]; Bflo4, *B. floridae* CT-type precursor 4 [GI:260812099]; Hsap_Calc, *H. sapiens* CT precursor [GI:179819]; Hsap_CGRP1, *H. sapiens* CT gene-related peptide (CGRP) 1 precursor [GI:269784661]; Hsap_IAPP, *H. sapiens* islet amyloid polypeptide (IAPP) precursor [GI:109255169]; Hsap_ADML, *H. sapiens* adrenomedullin precursor [GI:675022745]; Hsap_ADM2, *H. sapiens* adrenomedullin 2 precursor [GI:41016725]; Ctel, *C. teleta* CT-type precursor [GI: 161220966]; Lgig1, *L. gigantea* CT-type precursor 1 [GI:163526287]; Lgig2, *L. gigantea* CT-type precursor 2 [GI:676481265].

ArCT is the third CT-type neuropeptide to be identified in an echinoderm, following the identification of CT-type peptides in the sea urchin *S. purpuratus* [9] and the sea cucumber *A. japonicus* [10]. In figure 13, we show an alignment of ArCT with CT-type peptides that have been identified in other deuterostomes and in lophotrochozoans, which also illustrates the antiquity of this bilaterian neuropeptide family. A conserved feature of these neuropeptides is the presence of the two cysteine residues in the N-terminal region, which—as highlighted above—form a disulfide bridge. Another conserved feature is a C-terminal amidated proline, although this character has been lost in some CT-type peptides that occur in vertebrates, such as CT-gene-related peptide (CGRP), islet amyloid polypeptide (IAPP) and adrenomedullins (figure 13).

CT-type peptides have also been identified in insects and other arthropods, and were discovered on account of their effects as diuretic hormones (DH31) [184]. However, the DH31-type peptides do not have the pair of cysteine residues that are characteristic of deuterostomian CT-type peptides. Interestingly, in annelids (e.g. *Capitella*), two genes encoding CT-type peptides have been identified: one encoding a CT-type peptide with two N-terminal cysteine residues and one encoding a DH31-type peptide without two N-terminal cysteine residues [5,8]. More recently, a second gene encoding one or multiple CT-type peptides with two N-terminal cysteine residues has also been identified in several arthropod species [185]. It appears, therefore, that there was a duplication of a CT-type gene in a common ancestor of the protostomes, with the neuropeptide product of one copy retaining the N-terminal cysteine residues (CT-type) and the neuropeptide product of the other copy losing the N-terminal cysteine residues (DH31-type).

CT was first discovered in mammals as a peptide that is released from parafollicular cells of the thyroid gland, inhibits intestinal calcium ion (Ca^2+^) absorption and inhibits osteoclast activity in bones [186]. CT is encoded by a gene that also encodes CGRP, with alternative splicing giving rise to either prepro-CT (exons 1, 2, 3 and 4) or prepro-CGRP (exons 1, 2, 3, 5 and 6) [187]. CGRP is released by sensory nerves and is a potent vasodilator in mammals [188]. Aside from the diuretic actions of DH31-type peptides in insects [184], very little is known about the physiological roles of CT-type neuropeptides in invertebrates. With the discovery of ArCT in *A. rubens* and related peptides in other echinoderms [9,10], there are opportunities to address this issue using echinoderms as model systems.

#### 3.3.7. Precursor of two cholecystokinin-type peptides (ArCCKP)

A cholecystokinin (CCK)-type neuropeptide precursor in *A. rubens* **(**ArCCKP) was identified as a 163-residue protein comprising a predicted 22-residue N-terminal signal peptide and two putative CCK-type neuropeptides bounded by dibasic cleavage sites: RQSKVDDYGHGLFWG (ArCCK1) and GGDDQYGFGLFFG (ArCCK2) (figure 9*g*; GenBank: KT601716). The presence of C-terminal glycine residues in both of these sequences is indicative of post-translational modifications giving rise to a C-terminal amide group on the mature peptides. Furthermore, an additional potential post-translational modification for these peptides is sulfation of the tyrosine residues (underlined above), as this is a common characteristic of CCK-type neuropeptides in other species [189].

CCK-type peptides have a widespread phylogenetic distribution in bilaterian animals [5,6,190–192] and the antiquity of CCK-type peptides was first revealed with the discovery of CCK-type sulfakinin (SK) peptides in insects [193,194]. CCK-type peptides have been identified throughout the vertebrates [195] and in deuterostomian invertebrates, including the urochordate *C. intestinalis* [196], the hemichordate *S. kowalevskii* [5] and in the echinoderm *S. purpuratus* [5]. Interestingly, however, CCK-type peptides and receptors appear to have been lost in the cephalochordate *B. floridae* [5].

**Figure 14.**
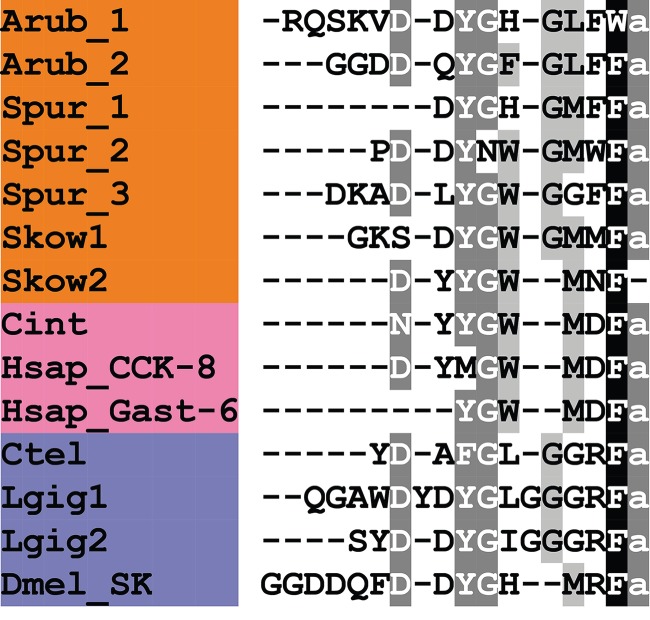
Alignment of ArCCK1 and ArCCK2 with other cholecystokinin (CCK)-type peptides. Accession numbers for the corresponding precursor proteins are: Arub, *A. rubens* CCK-type precursor [GenBank: KT601716]; Spur, *S. purpuratus* CCK-type precursor [GI:390355380]; Skow1, *S. kowalevskii* CCK-type precursor 1 [GI:585688033]; Skow2, *S. kowalevskii* CCK-type precursor 2 [GI:187061456]; Cint, *C. intestinalis* cionin precursor [GI:10799472]; Hsap_CCK-8, *H. sapiens* CCK precursor [GI:30582820]; Hsap_Gast-6, *H. sapiens* gastrin precursor [GI:47481291]; Ctel, *C. teleta* CCK-type precursor [GI:161296032]; Lgig1, *L. gigantea* CCK-type precursor 1 [GI:161296032]; Lgig2, *L. gigantea* CCK-type precursor 2 [GI:52414496]; Dmel_SK, *D. melanogaster* sulfakinin (SK) precursor [GI:386765036].

In figure 14, we compare ArCCK1 and ArCCK2 with CCK-type peptides identified in other species. Most vertebrate CCK-type peptides typically share the conserved C-terminal octapeptide motif DYMGWMDF-NH_2_, whereas most SK-type peptides, for example in *D. melanogaster*, typically share the conserved C-terminal heptapeptide motif DYGHMRF-NH_2_ (figure 14). ArCCKP comprises two putative CCK-type peptides with the C-terminal octapeptide motifs DYGHGLFW-NH_2_ (ArCCK1) and QYGFGLFF-NH_2_ (ArCCK2), which share the common motif x_1_YGx_2_GLFx_3_-NH_2_, with x_3_ representing a shared hydrophobic residue. This motif shares sequence similarity with both the vertebrate CCK-type and protostomian SK-type motifs, including the likely presence of a conserved O-sulfated tyrosine residue and an amidated phenylalanine residue (with the exception of ArCCK1, which has a C-terminal tryptophan residue; figure 14).

CCK and the related peptide hormone gastrin have a common C-terminal tetrapeptide sequence (WMDF-NH_2_) that is required for biological activity and indicative of a common evolutionary origin [195,197,198]. CCK/gastrin-type peptides have numerous roles in the gastrointestinal system and central nervous system of mammals. In the gastrointestinal system, roles in regulation of gallbladder contraction, gastrointestinal motility and pancreatic secretion of digestive enzymes have been identified [199], and in the CNS these peptides are implicated in learning and memory, angiogenesis, nociception and regulation of food intake [195,200–203].

SK-type peptides in insects are myotropic on the gut [193,194,204], heart [205] and body wall muscles [206]. It has also been discovered that SKs regulate food intake in multiple insect species including the desert locust *Schistocerca gregaria* [207], regulate digestive enzyme release in the beetle *Rhynchophorus ferrugineus* [208] and the moth *Opisina arenosella* [209], and affect digestive enzyme release and fat storage in the nematode *C. elegans* [191]. It appears, therefore, that the CCK/SK-type neuropeptide system has ancient roles in regulating physiological processes associated with feeding and digestion. The discovery of ArCCKP in the starfish *A. rubens* provides an opportunity to investigate the physiological roles of CCK-type peptides in an echinoderm species, in particular with respect to processes associated with feeding and digestion.

#### 3.3.8. Precursors of orexin-type peptides (ArOXP1 and ArOXP2)

An orexin (OX)-type neuropeptide precursor in *A. rubens* (ArOXP1) was identified as a 112-residue protein comprising a predicted 24-residue N-terminal signal peptide and an OX-type peptide sequence followed by a dibasic cleavage site (figure 9*h*; GenBank: KT601717). The presence of a C-terminal glycine residue is indicative of a post-translational modification that gives rise to a C-terminal amide group on the mature peptide. It is noteworthy that the putative OX-type peptide contains six cysteine residues, which may form up to three disulfide bridges. This contrasts with OX-type peptides in vertebrates that have two intra-chain disulfide bridges formed by four cysteine residues [210]. A second OX-type neuropeptide precursor in *A. rubens* (ArOXP2) was identified as a 161-residue protein comprising a predicted 26-residue N-terminal signal peptide and an OX-type peptide sequence, followed by a dibasic cleavage site (figure 9*i*; GenBank: KT601718). As with ArOXP1, the presence of a C-terminal glycine residue is indicative of a post-translational modification that gives rise to a C-terminal amide group and the presence of six cysteine residues is indicative of three disulfide bridges in the mature peptide.

OX-type peptides are a family of neuropeptides with a widespread phylogenetic distribution indicative of an ancestral bilaterian origin [5,6]. Thus, OX-type peptides, despite sharing little sequence similarity, have recently been found to be homologous to insect allatotropin (AT)-type peptides based on receptor orthology and precursor structure [5,6]. AT-type peptides have been identified in arthropods [211,212] and in lophotrochozoans, including molluscs [7,213,214] and annelids [8,215]. Interestingly, however, AT-type peptides and receptors appear to have been lost in nematodes and *Drosophila* [5,6].

**Figure 15.**
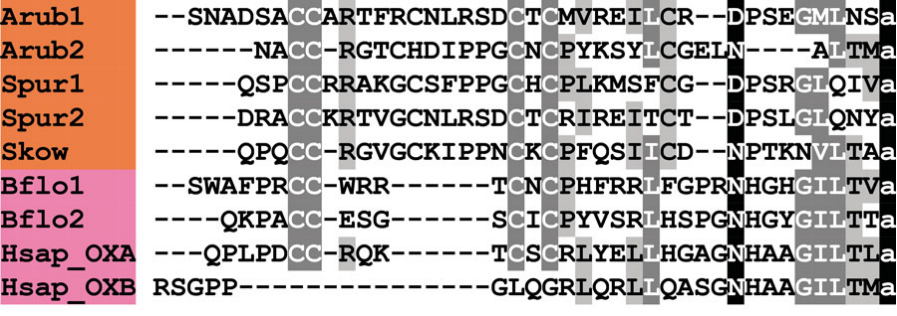
Alignment of ArOX1 and ArOX2 with other orexin (OX)-type peptides. Accession numbers for the corresponding precursor proteins are: Arub1, *A. rubens* OX-type precursor 1 [GenBank: KT601717]; Arub2, *A. rubens* OX-type precursor 2 [GenBank: KT601718]; Spur1, *S. purpuratus* OX-type precursor 1 [GI:346420309]; Spur2, *S. purpuratus* OX-type precursor 2 [GI:346419879]; Skow, *S. kowalevskii* OX-type precursor [GI:585662697]; Bflo1, *B. floridae* OX-type precursor 1 [GI:260807454]; Bflo2, *B. floridae* OX-type precursor 2 [GI:260780718]; Hsap_OX, *H. sapiens* OX precursor [GI:4557634].

In figure 15, we compare the sequences of ArOX1 and ArOX2 with OX-type peptides that have been identified in other deuterostomes. ArOX1 and ArOX2 are the second members of the OX neuropeptide family to be identified in echinoderms, following on from the identification of two OX-type precursors in the sea urchin *S. purpuratus* (SpOXP1 and SpOXP2) [5,6]. As in *A. rubens*, both of the OX-type peptides in *S. purpuratus* have six cysteine residues, suggesting the presence of three disulfide bridges in the mature peptides. Interestingly, an OX-type peptide in the hemichordate *S. kowalevskii* also has six cysteine residues [5,6] and therefore it appears that this feature may be a characteristic of ambulacrarian OX-type peptides. OX precursors in vertebrates comprise two OXs termed OX-A and OX-B [216]. OX-A contains four cysteine residues that form two intramolecular disulfide bridges, whereas OX-B does not contain cysteine residues. By way of comparison, two OX-type precursors in the cephalochordate *B. floridae* comprise peptides that are similar to vertebrate OX-A-type peptides, with four cysteine residues, and thus this appears to be a chordate characteristic.

OXs were originally discovered as hypothalamic neuropeptides that stimulate food intake in mammals [210,217]. However, it has subsequently been discovered that OXs also stimulate wakefulness and energy expenditure [218]. Similarly, OXs have also been shown to regulate feeding behaviour and the processes of sleep and wakefulness in teleost fish [219]. The ATs, the protostomian homologues of OXs [5,6], were first identified as peptides that stimulate the synthesis and secretion of JH from the corpora allata in insects [220,221]. Subsequently, other effects of ATs have been discovered, including cardioacceleration and the inhibition of active ion transport in the midgut of the tobacco hornworm *M. sexta* [220–222]. Currently, nothing is known about the physiological roles of OX-type peptides in echinoderms and other deuterostomian invertebrates, and therefore the discovery of ArOX1 and ArOX2, as reported here, provides an exciting opportunity to now address this issue.

#### 3.3.9. Precursor of a luqin-type neuropeptide (ArLQP)

A luqin (LQ)-type neuropeptide precursor in *A. rubens* (ArLQP) was identified as a 106-residue protein comprising a predicted 44-residue N-terminal signal peptide and a putative LQ-type peptide sequence (EEKTRFPKFMRWG) followed by a dibasic cleavage site (figure 9*j*; GenBank: KT601719). The presence of a C-terminal glycine residue is indicative of a post-translational modification giving rise to a C-terminal amide group on the putative mature peptide (EEKTRFPKFMRW-NH_2_).

**Figure 16.**
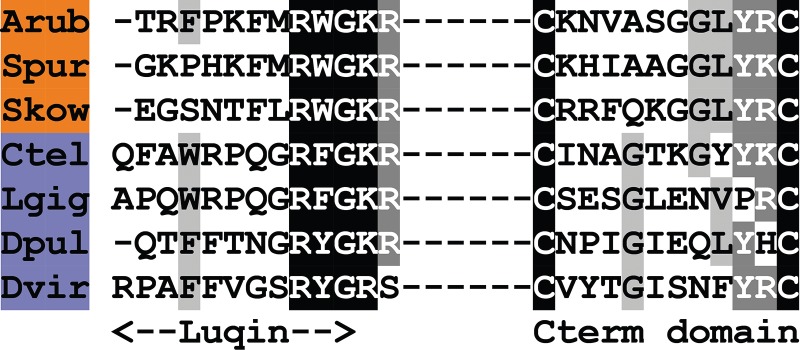
Alignment of ArLQ with other luqin (LQ)-type peptides and alignment of a conserved C-terminal domain of LQ-type precursor proteins. Accession numbers for the corresponding precursor proteins are: Arub, *A. rubens* LQ-type precursor [GenBank: KT601719]; Spur, *S. purpuratus* LQ-type precursor [GI:390331828]; Skow, *S. kowalevskii* LQ-type precursor [GI:585716464]; Ctel, *C. teleta* LQ-type precursor [GI:161280144]; Lgig, *L. gigantea* LQ-type precursor [GI:163510328]; Dpul, *D. pulex* LQ-type precursor [JGI:251691]; Dvir, *Drosophila virilis* LQ-type precursor [GI: 968114152].

Comparison of the LQ-type neuropeptide precursor in *A. rubens* with LQ-type precursor proteins that have been identified in other species reveals similarities (figure 16). Thus, in *A. rubens*, the precursor comprises a single putative neuropeptide (EEKTRFPKFMRW-NH_2_), which is also a feature of LQ-type precursors in other echinoderms (e.g. sea urchin *S. purpuratus*), in the hemichordate *S. kowalevskii* and in lophotrochozoans. This contrasts with precursor proteins in the ecdysozoans comprising multiple copies of LQ-type RYamides [6], which is probably a derived characteristic. Another feature of the *A. rubens* LQ precursor is two cysteine residues separated by a 10-residue peptide sequence in its C-terminal region, which are also a characteristic of LQ-type precursors in other invertebrates (figure 16). The LQ-type neuropeptide in *A. rubens* has a putative C-terminal RWamide motif, a feature that is shared by LQs in other echinoderms and in the hemichordate *S. kowalevskii*. Thus, this appears to be a characteristic of ambulacrarian LQs, which contrasts with the RFamide motif of lophotrochozoan LQs and the RYamide motif of ecdysozoan LQs (or ‘RYamides’). Comparison of echinoderm LQs reveals a high level of sequence identity, with the C-terminal sequence KFMRW-NH_2_ a shared feature of LQs in *A. rubens*, *S. purpuratus* (figure 16) and *A. japonicus* [10].

LQ was first identified in the mollusc *A. californica* and named on account of the expression of its precursor protein in the dorsal left upper quadrant cells of the abdominal ganglion [223]. Prior to the discovery of LQ in *A. californica*, a closely related neuropeptide termed *Achatina* cardio-excitatory peptide-1 or ACEP-1 was isolated from the African giant snail *Achatina fulica* on account of its excitatory effect on the heart and other muscles [224]. Subsequently, a closely related peptide termed *Lymnaea* cardio-excitatory peptide or LyCEP, which also has a cardio-excitatory effect, was isolated from the pond snail *Lymnaea stagnalis* [225]. More recently LQ-type peptides have been identified in a number of lophotrochozoans, including other molluscs [7] and annelids [8]. Very little is currently known about the physiological roles of the RYamides that are recognized as ecdysozoan (arthropods and nematodes) homologues of LQ [5,6]. However, evidence of a possible role in regulation of feeding behaviour in *Drosophila* has been reported [226].

The discovery of LQ-type peptides in several deuterostomian invertebrates [5,6,10] has revealed that the evolutionary origin of this neuropeptide family dates back to the common ancestor of the Bilateria. However, LQ-type peptides and receptors have not been found in the chordates, indicating that this neuropeptide signalling system may have been lost in the chordate lineage. At present nothing is known about the physiological roles of LQ-type peptides in deuterostomian invertebrates and therefore the discovery of ArLQP has provided a new opportunity to address this issue using starfish as an experimental system.

#### 3.3.10. Precursor of pedal peptide-type neuropeptides (ArPPLNP)

**Figure 17.**
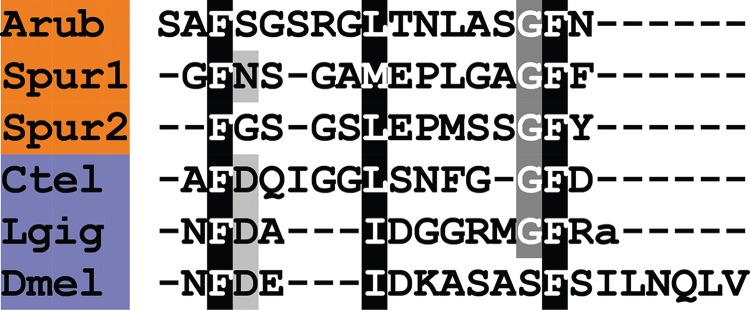
Alignment of representative *A. rubens* PP/orcokinin-type peptide with other PP/orcokinin-type peptides. Accession numbers for the corresponding precursor proteins are: Arub, *A. rubens* PP/orcokinin-type precursor [GenBank: KT601720]; Spur1, *S. purpuratus* PP/orcokinin-type precursor 1 [GI:390335272]; Spur2, *S. purpuratus* PP/orcokinin-type precursor 2 [GI:390352581]; Ctel, *C. teleta* PP-type precursor [GI:161190484]; Lgig, *L. gigantea* PP-type precursor [GI:163513756]; Dmel, *D. melanogaster* orcokinin precursor [GI:442624594].

A partial 325-residue sequence of a pedal peptide (PP)-type precursor (ArPPLNP) was identified in *A. rubens*, comprising a 31-residue N-terminal signal peptide and seven putative neuropeptides bounded by dibasic cleavage sites (figure 9*k*; GenBank: KT601720). The putative peptides derived from ArPPLNP share sequence similarity with peptides derived from two PP-type precursors that were recently identified in the sea urchin *S. purpuratus* (SpPPLNP1 and SpPPLNP2) [9]. Furthermore, as illustrated in figure 17, representative neuropeptides derived from ArPPLNP, SpPPLNP1 and SpPPLNP2 share sequence similarity with PP-type peptides in lophotrochozoans (e.g. *Capitella*, *Lottia*) and orcokinin-type peptides in arthropods (e.g. *Drosophila*). Indeed, it was the discovery of SpPPLNP1 and SpPPLNP2 in *S. purpuratus* that first revealed the widespread phylogenetic distribution and urbilaterian origin of PP/orcokinin-type peptides [9], a finding that has been supported by subsequent studies [6].

PP was first discovered in the mollusc *A. californica* as a peptide that causes contraction of pedal muscles [227,228]; it also stimulates beating of cilia associated with the foot [229]. Orcokinin was first isolated from abdominal nerve cord extracts of the crayfish *Orconectus limosus* on account of its effect in stimulating hindgut myoactivity [230]. Orcokinin-type peptides have subsequently been identified in a number of arthropods and attributed a range of functions, including stimulation of the prothoracic gland and regulation of ecdysteroidogenesis in the silk moth *Bombyx mori* [231], and regulation of circadian and seasonal physiology in the cockroach *Leucophaea maderae* [232–234].

Currently, nothing is known about the physiological functions of PP/orcokinin-type peptides in echinoderms. With the discovery of ArPPLNP in *A. rubens*, as reported here, an opportunity to address this issue in starfish has been provided.

#### 3.3.11. Precursors of GPA2-type and GPB5-type glycoprotein hormones

**Figure 18.**
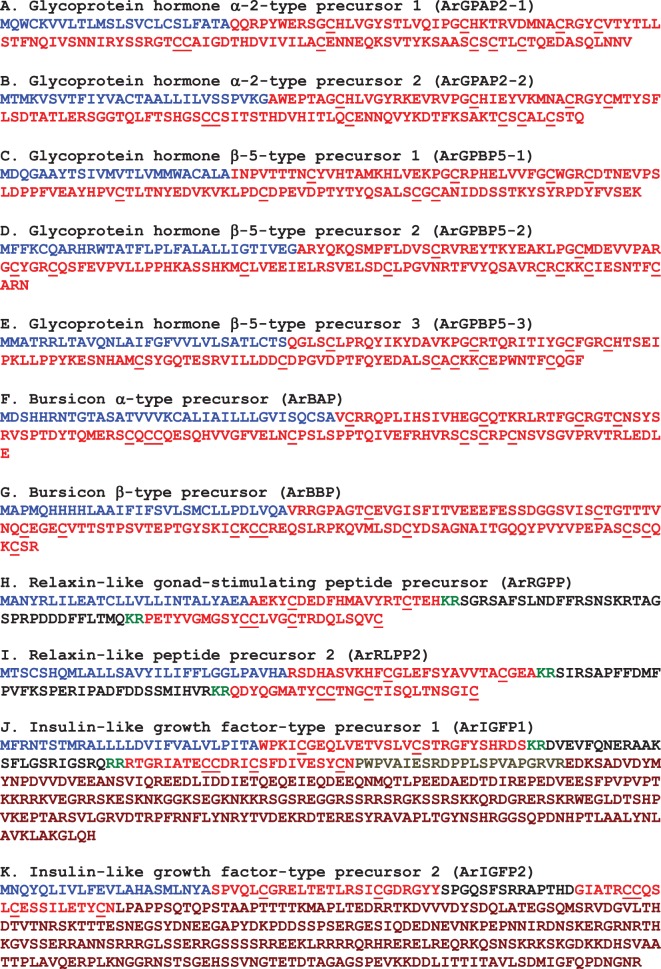
Precursors of cysteine-rich peptide hormones and growth factors in *A. rubens*. Predicted signal peptides are shown in blue, putative peptide hormones/growth-factors are shown in red (with cysteine (C) residues underlined), C-terminal glycine (G) residues that are putative substrates for amidation are shown in orange and putative dibasic cleavage sites are shown in green. For ArIGFP1 and ArIGFP2, the C-peptide is shown in black, the D-domain (for ArIGFP1) is shown in olive and the E-domain is shown in maroon.

A glycoprotein hormone *α*-2 (GPA2)-type precursor in *A. rubens* (ArGPAP2-1) was identified as a 135-residue protein comprising a predicted 24-residue N-terminal signal peptide followed by a 111-residue polypeptide that shares sequence similarity with GPA2-type subunits (figure 18*a*; GenBank: KT601721). A second GPA2-type precursor (ArGPAP2-2) was identified as a 130-residue protein comprising a predicted 28-residue N-terminal signal peptide followed by a 102-residue polypeptide that shares sequence similarity with GPA2-type subunits (figure 18*b*; GenBank: KT601722). It is noteworthy that both ArGPA2-1 and ArGPA2-2 contain 10 cysteine residues (figure 18*a*,*b*), which, in accordance with glycoprotein hormone subunits in other phyla, are likely to form five disulfide bridges [125].

A glycoprotein hormone *β*-5 (GPB5)-type precursor in *A. rubens* (ArGPBP5-1) was identified as a 136-residue protein comprising a predicted 24-residue N-terminal signal peptide followed by a 112-residue polypeptide that shares sequence similarity with GPB5-type subunits (figure 18*c*; GenBank: KT601723). A second GPB5-type precursor (ArGPBP5-2) was identified as a 141-residue protein comprising a predicted 31-residue N-terminal signal peptide followed by a 110-residue polypeptide that shares sequence similarity with GPB5-type subunits (figure 18*d*; GenBank: KT601724). A third GPB5-type precursor (ArGPBP5-3) was identified as a 130-residue protein comprising a predicted 30-residue N-terminal signal peptide followed by a 100-residue polypeptide sequence sharing sequence similarity with GPB5-type subunits (figure 18*e*; GenBank: KT601725). It is noteworthy that ArGPB5-1 contains eight cysteine residues while both ArGPB5-2 and ArGPB5-3 contain 10 cysteine residues (figure 18*c*–*e*), which are likely to form four or five disulfide bridges, respectively, in accordance with glycoprotein hormone subunits in other phyla [125].

**Figure 19.**
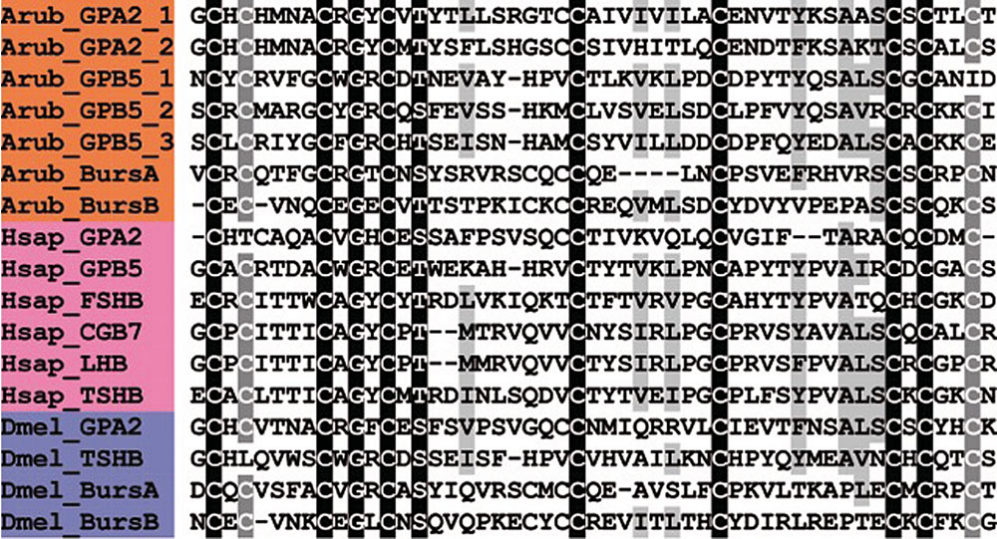
Alignment of glycoprotein/bursicon hormone-type precursors. Accession numbers for the corresponding precursor proteins are: Arub_GPA2_1, *A. rubens* glycoprotein hormone *α*-2 (GPA2)-type precursor 1 [GenBank: KT601721]; Arub_GPA2_2, *A. rubens* GPA2-type precursor 2 [GenBank: KT601722]; Arub_GPB5_1, *A. rubens* glycoprotein hormone *β*-5 (GPB5)-type precursor 1 [GenBank: KT601723], Arub_GPB5_2, *A. rubens* GPB5-type precursor 2 [GenBank: KT601724]; Arub_GPB5_3, *A. rubens* GPB5-type precursor 3 [GenBank: KT601725]; Arub_BursA, *A. rubens* bursicon-*α*-type precursor [GenBank: KT601726]; Arub_BursB, *A. rubens* bursicon-*β*-type precursor [GenBank: KT601727]; Hsap_GPA2, *H. sapiens* GPA2 precursor [GI:189491650]; Hsap_GPB5, *H. sapiens* GPB5 precursor [GI:21427593]; Hsap_FSHB, *H. sapiens* follicle-stimulating hormone (FSH) *β*-precursor [GI:124014246]; Hsap_CGB7, *H. sapiens* chorionic gonadotropin (CG) *β*-polypeptide 7 precursor [GI:15451749]; Hsap_LSHB, *H. sapiens* luteinizing hormone (LH) *β*-polypeptide precursor [GI:15431286]; Hsap_TSHB, *H. sapiens* the putative IGF-type receptor (TSH) *β*-precursor [GI:47479817]; Dmel_GPA2, *D. melanogaster* GPA2 precursor [GI:320546230]; Dmel_TSHB, *D. melanogaster* glycoprotein hormone *β*-subunit-related protein precursor [GI:21427595]; Dmel_BursA, *D. melanogaster* bursicon-*α* precursor [GI:665394724]; Dmel_BursB, *D. melanogaster* bursicon-*β* precursor [GI:62392020].

Alignment of ArGPA2-1, ArGPA2-2, ArGPB5-1, ArGPB5-2 and ArGPB5-3 with glycoprotein hormones in humans and *Drosophila* and with the related bursicon-type hormones (see below) reveals seven conserved cysteine residues and a conserved glycine residue. A serine or threonine residue that is located one residue after the fourth cysteine residue in all five of the *A. rubens* glycoprotein hormones is another conserved feature (figure 19).

The glycoprotein hormones are a family of cysteine-rich polypeptide hormones with a phylogenetic distribution indicative of an evolutionary ancestry that dates back to the common ancestor of the Bilateria [235]. The prototypical members of the glycoprotein hormone family are the mammalian gonadotropins LH, FSH choriogonadotropin (CG) and TSH [236]. These are heterodimeric glycoproteins comprising a common *α*-subunit and a unique *β*-subunit, which is crucial for receptor specificity. The *α*-subunit has 10 cysteine residues forming five disulfide bridges, while the *β*-subunit has 12 cysteine residues forming six disulfide bridges, with the formation of *α*/*β* dimers necessary for biological activity [125].

Sequencing of the human genome revealed a novel member of the glycoprotein hormone family, which is now known as thyrostimulin [237]. Thyrostimulin is a heterodimeric glycoprotein comprising an *α*-subunit termed GPA2 and a *β*-subunit termed GPB5, and it acts as a ligand for TSH receptors [238]. GPA2- and GPB5-type subunits have subsequently been identified in other vertebrates [237] and in deuterostomian invertebrates, including urochordates [239,240], the cephalochordate *B. floridae* [241], the hemichordate *S. kowalevskii* [239], and the echinoderm species *S. purpuratus* [11] and *A. japonicus* [10]. GPA2- and GPB5-type subunits have also been identified in ecdysozoans, including arthropods [242] and nematodes [235], and in lophotrochozoans, including molluscs [7] and annelids [8]. Thus, the phylogenetic distribution of GPA2- and GPB5-type subunits indicates that these subunits have an ancestral bilaterian origin, with the *α*- and *β*-subunits of vertebrate LH, FSH, CG and TSH probably having evolved from GPA2- and GPB5-type subunits, respectively, in the vertebrate lineage [243].

The physiological roles of GPA2/GPB5-type hormones in invertebrates have yet to be characterized extensively, but it has been shown that GPA2/GPB5-type hormones modulate ionic and osmotic balance in insects [244,245]. To date, nothing is known about the physiological roles of GPA2/GPB5-type hormones in echinoderms. Therefore, the discovery of glycoprotein-type hormones in *A. rubens*, as reported here, provides a new opportunity to address this issue.

#### 3.3.12. Precursors of bursicon-type hormones (ArBAP and ArBBP)

A bursicon-*α*-type precursor in *A. rubens* (ArBAP) was identified as a 139-residue protein comprising a predicted 35-residue N-terminal signal peptide followed by a 104-residue polypeptide sequence sharing sequence similarity with bursicon-*α*-type subunits (figure 18*f*; GenBank: KT601726). A bursicon-*β*-type precursor in *A. rubens* (ArBBP) was identified as a 142-residue protein comprising a predicted 30-residue N-terminal signal peptide followed by a 112-residue polypeptide sequence sharing sequence similarity with bursicon-*β*-type subunits (figure 18*g*; GenBank: KT601727). Both ArBAP and ArBBP contain 11 cysteine residues, in common with the prototypical bursicon-type subunits in insects (figure 19).

Bursicon was originally discovered in insects on account of its effect in causing cuticular tanning [246]. It is heterodimeric protein formed by two subunits, bursicon-*α* and bursicon-*β*, which are derived from separate precursor proteins [247]. Analysis of the sequences of these subunits reveals that bursicon is a member of the glycoprotein hormone family (see above). Bursicon-type subunits have been identified in a variety of insects and in other arthropods [248], and a role in regulation of cuticle hardening and ecdysis has been demonstrated in crustaceans [249–251]. Bursicon-type subunits have also been identified in lophotrochozoans, including molluscs [7] and annelids [8], and in deuterostomian invertebrates, including the echinoderm species *S. purpuratus* [11], *A. japonicus* [10] and now *A. rubens*. Thus, based on the phylogenetic distribution of bursicons, the evolutionary origin of this peptide hormone family dates back to the common ancestor of the Bilateria.

The physiological roles of bursicon-type hormones outside of the arthropods are unknown. The discovery of bursicon-type hormones in *A. rubens*, as reported here, provides a new opportunity to address this issue in an echinoderm species.

#### 3.3.13. Precursor of relaxin-like gonad-stimulating peptide (ArRGPP)

**Figure 20.**
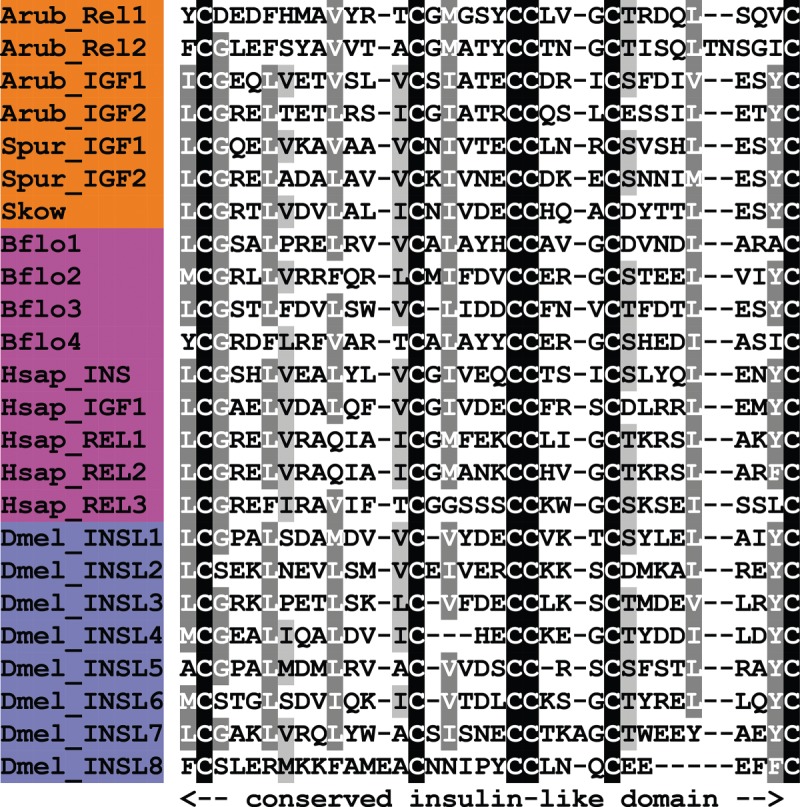
Alignment of insulin/relaxin/insulin-like growth factor (IGF)-type precursors. Accession numbers for the corresponding precursor proteins are: Arub_Rel1, *A. rubens* relaxin-like gonad-stimulating peptide precursor [GenBank: KT601728]; Arub_Rel2, *A. rubens* relaxin-like peptide precursor 2 [GenBank: KT601729]; Arub_IGF1, *A. rubens* IGF-type precursor 1 [GenBank: KT601730]; Arub_IGF2, *A. rubens* IGF-type precursor 2 [GenBank: KT601731]; Spur_IGF1, *S. purpuratus* IGF-type precursor 1 [GI:390333650]; Spur_IGF2, *S. purpuratus* IGF-type precursor 2 [GI:390333648]; Skow, *S. kowalevskii* IGF-type precursor [GI:187064073]; Bflo1, *B. floridae* IGF-type precursor 1 [JGI:72897]; Bflo2, *B. floridae* IGF-type precursor 2 [JGI:74371]; Bflo3, *B. floridae* IGF-type precursor 3 [JGI:77763]; Bflo4, *B. floridae* IGF-type precursor 4 [JGI:100967]; Hsap_INS, *H. sapiens* insulin (INS) precursor [GI:186429]; Hsap_IGF1, *H. sapiens* IGF precursor [GI:49456676]; Hsap_REL1, *H. sapiens* relaxin-1 precursor [GI:35932]; Hsap_REL2, *H. sapiens* relaxin-2 precursor [GI:35926]; Hsap_REL3, *H. sapiens* relaxin-3 precursor [GI:17484095]; Dmel_INSL1, *D. melanogaster* insulin-like (INSL) peptide 1 precursor [GI:317423340]; Dmel_INSL2, *D. melanogaster* INSL peptide 2 precursor [GI:442631434]; Dmel_INSL3, *D. melanogaster* INSL peptide 3 precursor [GI:221331056]; Dmel_INSL4, *D. melanogaster* INSL peptide 4 precursor [GI:442631435]; Dmel_INSL5, *D. melanogaster* INSL peptide 5 precursor [GI:320545737]; Dmel_INSL6, *D. melanogaster* INSL peptide 6 precursor [GI:442614930]; Dmel_INSL7, *D. melanogaster* INSL peptide 7 precursor [GI:386763756]; Dmel_INSL8, *D. melanogaster* INSL peptide 8 precursor [GI:386771312].

A relaxin-like gonad-stimulating peptide precursor in *A. rubens* (ArRGPP) was identified as a 109-residue protein comprising sequentially (i) a predicted 26-residue N-terminal signal peptide, (ii) a 20-residue polypeptide comprising two cysteine residues (B-chain), (iii) a dibasic cleavage site, (iv) a connecting peptide (C-peptide) domain (residues 49–82), (v) a dibasic cleavage site and (vi) a 25-residue polypeptide comprising four cysteine residues (A-chain) (figure 18*h*; GenBank: KT601728). The A-chain has the cysteine motif CCxxxCxxxxxxxxC, which is characteristic of the insulin/insulin-like growth factor (IGF)/relaxin superfamily (figure 20). More specifically, the final residue of the A-chain is a cysteine, which is characteristic of the relaxin/insulin-like (INSL) sub-class as opposed to the insulin/IGF sub-class [252]. The B-chain has the cysteine motif CxxxxxxxxxxxC, which is characteristic of the insulin/IGF/relaxin superfamily (figure 20). However, the B-chain does not contain the relaxin-specific receptor-binding motif RxxxRxxI/V characteristic of vertebrate relaxin-like peptides [253]. Informed by the biochemical characterization of RGP in *A. pectinifera* [39], the predicted mature product of ArRGPP is a heterodimeric protein comprising A/B-chains, with two inter-chain disulfide bridges and an intra-chain disulfide bridge in the A-chain.

RGP was first identified in the starfish species *A. pectinifera* [39] as the long-sought-after ‘gonad-stimulating substance’ or GSS. The existence of GSS was first reported in 1959 as a component of starfish radial nerves that triggers gamete release in starfish [254]. Subsequently, it was characterized as a peptide hormone [255–257], but 50 years elapsed before its molecular identity was finally determined, revealing that it is a member of the insulin/IGF/relaxin superfamily [39]. The existence of GSS in many other starfish species has been reported [258]. Thus, GSS activity was first detected in extracts of *A. forbesi* and early efforts to determine the chemical identity of GSS focused on *A. amurensis* [258]. Recently, an RGP-like precursor sharing a high level of sequence similarity with ArRGPP was identified in *A. amurensis*, and experimental tests revealed that synthetic *A. amurensis* RGP triggers gamete spawning and oocyte maturation in this species [259]. Therefore, it is very likely that ArRGP also triggers gamete spawning and oocyte maturation in *A. rubens*.

#### 3.3.14. Precursor of relaxin-like peptide 2 (ArRLPP2)

A second relaxin-like peptide precursor in *A. rubens* (ArRLPP2) was identified as a 119-residue protein comprising sequentially (i) a predicted 30-residue N-terminal signal peptide, (ii) a 26-residue polypeptide comprising two cysteine residues (B chain), (iii) a dibasic cleavage site, (iv) a C-peptide domain (residues 59–91), (v) a dibasic cleavage site and (vi) a 26-residue polypeptide comprising four cysteine residues (A-chain) (figure 18*i*; GenBank: KT601729). It is noteworthy that the N-terminal glutamine residue in the B-chain could be subject to post-translational conversion to a pyroglutamate. Similarly to ArRGPP (or ‘ArRLPP1’), the A-chain has the cysteine motif CCxxxCxxxxxxxxC, while the B-chain has the cysteine motif CxxxxxxxxxxxC characteristic of the insulin/IGF/relaxin superfamily (figure 20). However, the B-chain does not contain the relaxin-specific receptor-binding motif RxxxRxxI/V characteristic of vertebrate relaxin-like peptides [253]. Thus, as with ArRGPP, the predicted bioactive product of ArRLPP2 is a relaxin-like heterodimeric protein comprising A/B-chains, with two inter-chain disulfide bridges and an intra-chain disulfide bridge in the A-chain.

The hormone relaxin was first identified in 1926 on account of its softening effect on the pubic ligament [260] and the sequence of a cDNA encoding the relaxin precursor was reported in 1981 [261]. Subsequently, other relaxin/INSL precursors have been identified in mammals and other vertebrates [262,263], and the diversity of relaxin/INSL genes in vertebrates is in part attributable to whole-genome duplication during early vertebrate evolution [264,265]. More specifically, it has been suggested that the relaxin/INSL genes are products of an ancestral system that originally consisted of three genes, two of which trace their origins back to the invertebrates [265].

The likely physiological role of ArRGP as a regulator of gamete maturation and spawning in *A. rubens* has been discussed above. The discovery of ArRLPP2 in *A. rubens* provides an opportunity to investigate the physiological roles of a second relaxin-like peptide in an echinoderm species.

#### 3.3.15. Precursors of insulin-like growth factors (ArIGFP1 and ArIGFP2)

ArIGFP1 is a 355-residue precursor protein comprising a predicted 27-residue N-terminal signal peptide and A–E domains that are characteristic of IGF-type precursors (figure 18*j*; GenBank: KT601730). The B-domain contains two cysteine residues (residues 32 and 44) and the A-domain contains four cysteine residues (residues 91, 92, 96 and 105), which are likely to form disulfide bridges and a peptide heterodimer between both chains based on the presence of this feature in the insulin/IGF/relaxin superfamily. ArIGFP1 has dibasic cleavage sites at the C-terminal and N-terminal of the B-domain (residues 28–55) and A-domain (residues 83–106), respectively, which would allow for the removal of the intervening C-peptide (residues 58–80) as in vertebrate insulin and relaxin. However, the ArIGFP1 A-domain also extends to a D-domain (residues 107–133) and E-domain (residues 134–355), a feature found in IGF-type precursors [266].

ArIGFP2 is a 343-residue precursor protein comprising a predicted 22-residue N-terminal signal peptide and characteristic IGF-type A-, B-, C- and E-domains (figure 18*k*; GenBank: KT601731). The B-domain contains two cysteine residues (residues 28 and 40) and the A-domain contains four cysteine residues (residues 66, 67, 71 and 80), which are likely to form disulfide bridges, and a peptide heterodimer between both chains based on the presence of this feature in the insulin/IGF/relaxin superfamily. However, unlike vertebrate insulin and relaxin, ArIGFP2 does not have dibasic cleavage sites at the C-terminal and N-terminal of the B-domain (residues 23–46) and A-domain (residues 61–81), respectively, indicating that the intervening C-peptide (residues 47–80) is not processed for removal. Furthermore, the ArIGFP2 A-domain extends to an E-domain (residues 82–343), a feature found in IGF-type precursors [266].

Comparison of ArIGFP1/2 with IGF1/2 precursors identified and subsequently characterized in *S. purpuratus* (SpIGF1/2) [11,267] reveals that both ArIGFP1/2 and SpIGF1/2 have an A–E domain organization, as found in IGF-type precursors. However, ArIGFP1 and SpIGF1 are more insulin/relaxin-like in relation to potential removal of the C-peptide, while ArIGFP2 and SpIGF2 are more IGF-like in relation to the C-peptide. Both the ArIGFP1/2 A-domains have the cysteine motif CCxxxCxxxxxxxxC, characteristic of the insulin/IGF/relaxin superfamily (figure 20). Similarly, the B-domains both have the cysteine motif CxxxxxxxxxxxC, characteristic of the insulin/IGF/relaxin superfamily (figure 20). Importantly, two disulfide bridges connecting the B- and A-domains and a single intra-disulfide bridge associated with the A-domain have been identified in SpIGF1/2 [267]. Interestingly, it has also been shown that SpIGF1 and SpIGF2 share more sequence similarity with each other compared with other insulin/IGF/relaxin superfamily members in other phyla, indicating that they may have arisen by gene duplication in the sea urchin or echinoderm lineage [267].

To date, the physiological function of IGF-type peptides in non-chordate deuterostomes, and in particular echinoderms, has not been extensively studied. It has previously been shown that mammalian insulin stimulates growth of the sea urchin embryo and that IGFs may be expressed in the sea urchin larval gut [268] and adult starfish gut [269], suggesting a role in digestive processes. Moreover, microarray data confirm expression in the sea urchin embryo [270]. The identification of SpIGF1 and SpIGF2 in the sea urchin *S. purpuratus* [11] has made it possible to investigate the roles of IGFs in echinoderms. It has recently been shown that SpIGF1 is expressed in both the stomach and intestine of feeding larvae, with differential expression dependent on nutrient availability, suggesting a role for SpIGF1 in digestive processes [267]. Furthermore, it has been shown that SpIGF2 is expressed in the gastrula foregut, while the putative IGF-type receptor (SpInsr) is expressed in the mesodermal cells at the tip of the archenteron [267]. Taken together, this suggests a role for SpIGF2 as a growth signal to stimulate coelomic pouch development [267].

### 3.4. Discovery of SALMFamide precursors and precursors of candidate neuropeptides in *A. rubens* that do share apparent sequence similarity with known neuropeptide families

#### 3.4.1. Precursors of SALMFamide neuropeptides

The SALMFamide neuropeptides S1 (GFNSALMF-NH_2_) and S2 (SGPYSFNSGLTF-NH_2_) were originally isolated from extracts of nerves dissected from *A. rubens* and the closely related starfish species *A. forbesi*; they were the first neuropeptides to be identified in an echinoderm species [21,22]. Here, we have identified transcripts that encode the S1 and S2 precursor proteins.

**Figure 21.**
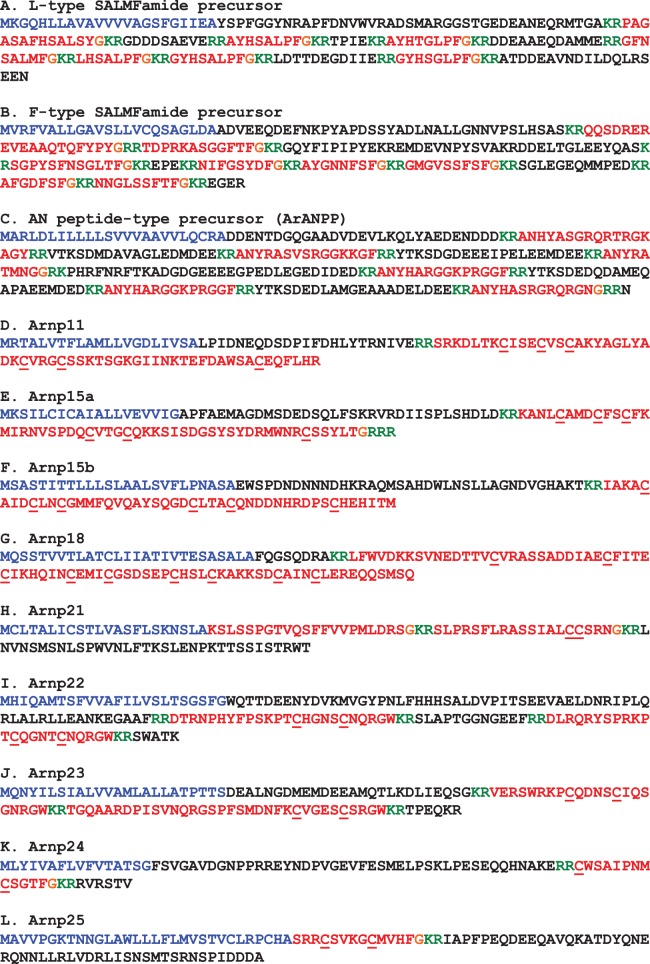
Precursors of putative neuropeptides in *A. rubens* that do not share apparent sequence similarity with known neuropeptide families. Predicted signal peptides are shown in blue, putative peptides are shown in red (with cysteine (C) residues underlined), C-terminal glycine (G) residues that are putative substrates for amidation are shown in orange and putative dibasic cleavage sites are shown in green.

The S1 precursor is a 210-residue protein comprising a predicted 23-residue N-terminal signal peptide and, bounded by dibasic cleavage sites, seven putative neuropeptide sequences that have a C-terminal glycine residue, which is a potential substrate for C-terminal amidation (figure 21*a*; GenBank: KT601732). The predicted neuropeptide products of the S1 precursor are S1 and six other putative L-type SALMFamides, which like S1 have the C-terminal motif S/TxLxF/Y-NH_2_ (where x is variable). Four of the novel SALMFamides are octapeptides, like S1, but one peptide is one residue shorter than S1 (LHSALPF-NH_2_) and another is longer than S1 (PAGASAFHSALSY-NH_2_).

The S2 precursor is a 233-residue protein comprising a predicted 23-residue N-terminal signal peptide and, bounded by dibasic cleavage sites, eight putative neuropeptide sequences that have a C-terminal glycine residue, which is a potential substrate for C-terminal amidation (figure 21*b*; GenBank: KP330476). The predicted neuropeptide products of the S2 precursor are S2, which like S1 is an L-type SALMFamide, and seven putative F-type SALMFamides, which have the C-terminal motif F/YxF/Y-NH_2_ (where x is variable). The predicted neuropeptide products of the S2 precursor range in size from just seven residues (AFGDFSF-NH_2_) to as many as 19 residues (QQSDREREVEAAQTQFYPY-NH_2_). Furthermore, the N-terminal glutamine residue of the latter peptide may be a substrate for conversion to pyroglutamate, which is a common post-translational modification of neuropeptides.

The sequences of the S1 precursor and S2 precursor in *A. rubens* are similar to those of orthologous proteins that were recently identified in the starfish species *Patiria miniata* [271]. Thus, in *P. miniata*, there is an L-type SALMFamide precursor, which comprises S1 and six other L-type SALMFamides, while an orthologue of the *A. rubens* S2 precursor in *P. miniata* comprises an S2-type peptide and eight F-type or F-type-like SALMFamides. SALMFamide precursors have also been identified in other echinoderms. As in starfish, sea urchins (class Echinoidea; e.g. *S. purpuratus*), sea cucumbers (class Holothuroidea; e.g. *A. japonicus*) and brittle stars (class Ophiuroidea; e.g. *Ophionotus victoriae*) have two SALMFamide precursor genes—an L-type precursor and an F-type precursor. In contrast, only a single SALMFamide gene has been identified in feather stars (class Crinoidea; e.g. *Antedon mediterranea*). As crinoids are basal to the other echinoderm classes phylogenetically, it has been proposed that the L-type and F-type SALMFamide precursors that occur in Asterozoa (Asteroidea and Ophiuroidea) and Echinozoa (Echinoidea and Holothuroidea) may have arisen by duplication of a gene encoding a protein similar to the SALMFamide precursor found in extant crinoids [272].

The physiological roles of SALMFamide neuropeptides in echinoderms have been investigated using *in vitro* and *in vivo* pharmacological methods. This has revealed that both L-type and F-type SALMFamides act as muscle relaxants [26–28,273]. More specifically, injection of S1 or S2 in *A. rubens* triggers cardiac stomach eversion, a process that occurs naturally when starfish feed extra-orally on prey such as mussels [26,27]. Consistent with this effect of S1 and S2 *in vivo*, both peptides cause dose-dependent relaxation of cardiac stomach preparations *in vitro* [27]. Furthermore, S1-immunoreactive and S2-immunoreactive nerve fibres are present in the innervation of the cardiac stomach, and therefore it is thought that endogenous release of S1 and/or S2 may be responsible, at least in part, for mediating cardiac stomach eversion when starfish feed [24–26].

With the discovery of the sequences of the S1 and S2 precursor proteins, as reported here, we now know that both S1 and S2 are derived from precursor proteins that contain other SALMFamides. Therefore, testing and comparing the effects of S1 and S2 *in vitro* is in fact not representative of physiological conditions. In the future it would be interesting to investigate the *in vitro* actions of the ‘cocktail’ of SALMFamides that are derived from the S1 precursor, as this will reveal pharmacological actions that are representative of physiological processes *in vivo*. Similarly, it will be interesting to compare the effects of S2 with the effects of F-type and F-type-like SALMFamides that are derived from the S2 precursor, and to compare the effects of peptides tested individually with the effects of the entire ‘cocktail’ of SALMFamides that are derived from the S2 precursor. Experimental studies such as these, using the starfish SALMFamides as a model system, may provide new insights into the functional significance of the general phenomenon of precursor proteins that give rise to ‘cocktails’ of structurally related neuropeptides.

One important issue that remains to be resolved is the relationship of echinoderm SALMFamide neuropeptides with neuropeptides that have been identified in other phyla. Insights into this issue would be gained if the receptor(s) that mediate the effects of SALMFamides were identified. Based on C-terminal sequence similarity shared with vertebrate gonadotropin-inhibitory hormone (GnIH)/NPFF-type neuropeptides (LxFamide) and QRFP-type neuropeptides (FxFamide), candidate receptors have been proposed [274]. However, definitive identification of SALMFamide receptors will require functional expression studies and the neural transcriptome sequence data that we have obtained for *A. rubens* provides a basis for this.

#### 3.4.2. Precursor of AN peptides (ArANPP)

ArANPP is a 274-residue precursor protein comprising a predicted 24-residue N-terminal signal peptide and six putative neuropeptides with an N-terminal alanine (A)/asparagine (N) (AN) motif, which are bounded by putative dibasic cleavage sites (figure 21*c*; GenBank: KT601733). Three of the predicted neuropeptides have a C-terminal glycine residue, which may be a substrate for post-translational conversion to an amide group. ArANPP was identified on account of its sequence similarity with the sea urchin AN peptide precursor SpANPP [9].

The AN peptides are a family of peptides that have to date only been identified in the echinoderms [9,10], and a relationship with neuropeptides identified in other phyla has yet to be determined. Some similarities with TK-type peptides have been noted [10], but with the discovery of other TK-type peptides in *A. rubens* (see above), it would appear that these similarities may be due to convergence. The physiological roles of AN peptides in echinoderms are unknown so the discovery of ArANPP has provided an opportunity to address this issue using the starfish *A. rubens* as a model experimental system.

#### 3.4.3. Arnp11

Arnp11 was identified and named on account of its similarity with Spnp11, a putative neuropeptide precursor in the sea urchin *S. purpuratus* [9]. It is a 103-residue precursor protein comprising a 21-residue N-terminal signal peptide followed by an 82-residue polypeptide sequence (residues 22–103) that contains a putative dibasic cleavage site at residues 45/46 (figure 21*d*; GenBank: KT601734). The N-terminal region of the protein (residues 22–44) contains six acidic residues (D or E), which indicates that this part of the protein may be an acidic spacer peptide. We propose that it is the 57-residue polypeptide formed by residues 47–103 that may be a secreted bioactive neuropeptide. It is noteworthy that the 57-residue polypeptide includes six cysteine residues, which may form up to three intramolecular disulfide bridges. Alternatively, a homodimeric protein could be formed by up to six intermolecular disulfide bridges.

The putative 57-residue polypeptide (Arn11) derived from Arnp11 shares sequence similarity with a putative 54-residue polypeptide (Spn11) derived from Spnp11, including the presence of six cysteine residues. However, these polypeptides do not share any apparent sequence similarity with neuropeptides or peptide hormones that have been identified in any other phyla. Nevertheless, neuropeptides of a similar size and with six cysteine residues have been identified in other animals. For example, eclosion hormone in insects [275] and a family of peptide hormones in crustaceans comprising molt-inhibiting hormone, vitellogenesis-inhibiting hormone and crustacean hyperglycaemic hormone [276–278] each comprise six cysteine residues that form three intramolecular disulfide bridges.

#### 3.4.4. Arnp15a and Arnp15b

Arnp15a and Arnp15b were identified and named on account of their similarity to Spnp15, a putative neuropeptide precursor in the sea urchin *S. purpuratus* [9].

Arnp15a is a 111-residue protein comprising a predicted 19-residue N-terminal signal peptide followed by a 92-residue polypeptide sequence (residues 20–111) that contains a dibasic cleavage site at residues 54/55 (figure 21*e*; GenBank: KT601735). The N-terminal region of the protein (residues 20–53) contains eight acidic residues (D or E), indicating that this part of the protein is an acidic spacer peptide. We propose that it is the 53-residue polypeptide formed by residues 56–108 that may be a secreted bioactive neuropeptide. The presence of six cysteine residues in the 53-residue polypeptide suggests that there may be up to three intramolecular disulfide bridges. Alternatively, a homodimeric protein could be formed by up to six intermolecular disulfide bridges.

Arnp15b is a 111-residue protein comprising a predicted 25-residue N-terminal signal peptide followed by an 86-residue polypeptide sequence (residues 26–111) that contains a dibasic cleavage site at residues 63/64 (figure 21*f*; GenBank: KT601736). The N-terminal region of the protein (residues 26–62) contains six acidic residues (D or E), indicating that this part of the protein may be an acidic spacer peptide. We propose that it is the 47-residue polypeptide formed by residues 65–111 that may be a secreted bioactive neuropeptide. The presence of six cysteine residues in the 47-residue polypeptide suggests that there may be up to three intramolecular disulfide bridges. Alternatively, a homodimeric protein could be formed by up to six intermolecular disulfide bridges.

#### 3.4.5. Arnp18

Arnp18 was identified and named on account of its similarity to Spnp18, a putative neuropeptide precursor in the sea urchin *S. purpuratus* [9]. Arnp18 is a 113-residue protein comprising a predicted 27-residue N-terminal signal peptide followed by an 86-residue polypeptide sequence (residues 28–113) that contains a putative dibasic cleavage site at residues 36/37 (figure 21*g*; GenBank: KT601737). We propose that it is the 76-residue polypeptide formed by residues 38–113 that may be a secreted bioactive neuropeptide (Arn18). It is noteworthy that Arn18 contains nine cysteine residues, which may form up to four intramolecular disulfide bridges. Alternatively, a homodimeric protein could be formed by up to nine intermolecular disulfide bridges. Arn18 and Spn18 do not share any apparent sequence similarity with neuropeptides or peptide hormones identified in any other phyla. However, neuropeptides similar in size comprising eight cysteine residues have been identified in other animals; for example, the anti-gonadotropic peptide schistosomin in the pond snail *L. stagnalis*, which is thought to have four intramolecular disulfide bridges [279].

#### 3.4.6. Arnp21

Arnp21 is a 102-residue protein comprising a predicted 22-residue N-terminal signal peptide followed by an 80-residue polypeptide sequence (residues 23–102) that contains putative dibasic cleavage sites at residues 45/46 and 67/68 (figure 21*h*; GenBank: KT601738). We propose that it is the 22-residue peptide formed by residues 23–44 (Arn21a) and the 20-residue peptide formed by residues 47–66 (Arn21b) that may be secreted bioactive neuropeptides. The presence of C-terminal glycine residues on both of these peptides is indicative of post-translational modifications giving rise to a C-terminal amide group on the mature peptides.

#### 3.4.7. Arnp22

Arnp22 is a 157-residue protein comprising a predicted 24-residue N-terminal signal peptide followed by a 133-residue polypeptide sequence (residues 25–157) that contains putative dibasic cleavage sites at residues 86/87, 112/113, 126/127, 136/137 and 151/152 (figure 21*i*; GenBank: KT601739). We propose that it is the 24-residue polypeptide formed by residues 88–111 (Arn22a) and the 23-residue polypeptide formed between residues 128–150 (Arn22b) that may form secreted bioactive neuropeptides. It is noteworthy that both Arn22a and Arn22b contain two cysteine residues that are separated by four amino acid residues and that may form intramolecular disulfide bridges. Alternatively, heterodimeric polypeptides could be formed by up to two intermolecular disulfide bridges. It is also noteworthy that both Arn22a and Arn22b have the same C-terminal pentapeptide sequence—NQRGW. Therefore, this conserved feature may be critical for the bioactivity of the candidate neuropeptides derived from Arnp22.

#### 3.4.8. Arnp23

Arnp23 is a 118-residue protein comprising a predicted 24-residue N-terminal signal peptide followed by a 94-residue polypeptide sequence (residues 25–118) that contains putative dibasic cleavage sites at residues 51/52, 75/76 and 111/112 (figure 21*j*; GenBank: KT601740). We propose that it is the 22-residue polypeptide formed by residues 53–74 (Arn23a) and the 34-residue polypeptide formed by residues 77–110 (Arn23b) that may form secreted bioactive neuropeptides. It is noteworthy that both Arn23a and Arn23b contain two cysteine residues that are separated by four amino acid residues and that may form intramolecular disulfide bridges. Alternatively, a heterodimeric protein could be formed by up to two intermolecular disulfide bridges. Interestingly, both Arn23a and Arn23b have the same C-terminal tripeptide sequence (RGW), which, as highlighted above, is a feature of Arn22a and Arn22b. This suggests that Arnp22 and Arnp23 may be related and, as with Arnp22, the conserved RGW motif may be critical for the bioactivity of the candidate neuropeptides derived from Arnp23.

#### 3.4.9. Arnp24

Arnp24 is an 83-residue protein comprising a predicted 16-residue N-terminal signal peptide followed by a 67-residue polypeptide sequence (residues 17–83) that contains putative dibasic cleavage sites at residues 60/61 and 76/77 (figure 21*k*; GenBank: KT601741). We propose that it is the 14-residue peptide formed by residues 62–75 that may form a secreted bioactive neuropeptide. The presence of a C-terminal glycine residue on the peptide is indicative of post-translational modification giving rise to a C-terminal amide group on the mature peptide. The putative neuropeptide (Arn24) contains two cysteine residues, which may form an intramolecular disulfide bridge. Alternatively, a homodimeric protein could be formed by up to two intermolecular disulfide bridges. Finally, it is noteworthy that the position of Arn24 in the C-terminal region of the precursor and the presence of two cysteine residues is reminiscent of SS/MCH-type neuropeptides (see above), which may provide clues towards functional characterization of Arn24 as a putative neuropeptide.

#### 3.4.10. Arnp25

Arnp25 is a 97-residue protein comprising a predicted 31-residue N-terminal signal peptide followed by a 66-residue polypeptide sequence (residues 32–97) that contains a putative dibasic cleavage site at residues 46/47 (figure 21*l*; GenBank: KT601742). We propose that it is the 14-residue peptide formed by residues 32–45 that may form a secreted bioactive neuropeptide. The presence of a C-terminal glycine residue on the peptide is indicative of post-translational modification giving rise to a C-terminal amide group on the mature peptide. It is also noteworthy that the putative neuropeptide (Arn25) contains two cysteine residues, which may form an intramolecular disulfide bridge. Alternatively, a homodimeric protein could be formed by up to two intermolecular disulfide bridges. It should also be noted that the two arginine residues preceding the first cysteine residue represent a potential dibasic cleavage site. If this were indeed a cleavage site then the putative neuropeptide Arn25 would be an 11-residue peptide and not a 14-residue peptide. Interestingly, Arnp25 shares some similarities with VP/OT-type neuropeptide precursors—the putative neuropeptide Arn25 is located proximal to the N-terminal signal peptide and, in common with VP/OT-type peptides, Arn25 is a putative C-terminally amidated peptide with two cysteine residues separated by four amino acid residues. These shared characteristics may, of course, have arisen by convergent evolution. Nevertheless, Arn25 represents an interesting candidate neuropeptide for further investigation.

## 4. Conclusions

The identification of 40 neuropeptide precursors in the starfish *A. rubens* has provided important new insights into the evolution and diversity of neuropeptide signalling systems. Most noteworthy are the discovery of the first kisspeptin (KP)-type and MCH-type precursor proteins to be identified in a non-chordate species. Other neuropeptide families that have been identified previously in protostomes and deuterostomes have been identified here for the first time in an ambulacrarian/echinoderm species including tachykinin (TK)-, somatostatin (ss)-, PDF- and CRH-type precursor proteins. However, it should be noted that assignment of neuropeptides as members of bilaterian neuropeptide families based solely on sequence data can be difficult because of sequence convergence or divergence. More definitive proof of relationships can be obtained by identification of the cognate receptors for neuropeptides [5], and this will be an important objective for future research on the candidate neuropeptides identified here. Furthermore, mass spectroscopic identification of the mature neuropeptides derived from the starfish neuropeptide precursors identified here will be an important prelude to their functional characterization.

This study provides the most comprehensive identification of neuropeptide precursors in an echinoderm, in comparison with our previous analyses of transcriptome sequence data from the sea urchin *S. purpuratus* and the sea cucumber *A. japonicus* [9,10]. Discovery of 40 neuropeptide precursors in *A. rubens* provides a rich resource that establishes this echinoderm species as a model system for neuropeptide research, building upon pioneering research that enabled the discovery and functional characterization of the first neuropeptides to be identified in echinoderms—the SALMFamide neuropeptides S1 and S2 [29]. Furthermore, functional characterization of neuropeptides facilitated by analysis *A. rubens* neural transcriptome sequence data has already commenced. Thus, identification of the precursor of the neuropeptide NGFFYamide in *A. rubens* enabled functional characterization of this neuropeptide as a neural regulator of cardiac stomach contraction and retraction in starfish [156]. Likewise, experimental studies directed towards functional characterization of other candidate neuropeptides identified here are ongoing. We anticipate that discovery of the physiological roles of starfish representatives of ancient bilaterian neuropeptide families will provide important new insights into the evolution of neuropeptide function in the animal kingdom, particularly in the context of a pentaradial bauplan, which is such a unique and fascinating characteristic of starfish and other echinoderms.

## Supplementary Material

Supplementary Figures S1–S40

## Data accessibility

Raw sequence data generated using Illumina HiSeq has been deposited in the Sequence Read Archive (SRA) (SRP068147; http://www.ncbi.nlm.nih.gov/sra/SRP068147). Novel nucleotide sequence data for neuropeptide precursors have been deposited in the GenBank database (accession nos. KT601705–KT601742). The datasets supporting this article have been uploaded as part of the electronic supplementary material.
