# Supplementary material for: Transcriptomic identification of starfish neuropeptide precursors yields new insights into neuropeptide evolution
Source: Open Biol. 2016 Feb 10;6(2):150224. doi: 10.1098/rsob.150224 (PMC4772807; doi:10.1098/rsob.150224)
Supplement: Supplementary Figures S1–S40 [file rsob150224supp1.pdf]

```
1                                                               t
2    atacctaagggccacgcgccgcaaaatgcacttatataaaaaaggcgggaaatctgtgag
63   tcaaacaaacgtttgggacaggagcagtcgagcctacgccacaacttacatcatcttata
123  gaatagaagaaagacaacagcaacatcttcccgtagttttgaccactccgctttttttcag
183  aactttctccatcgaggtggctggaaaaagcgacgcaagagggctttcgagtttgtcaga
243  accctgagtacgatcattgacgaaatggagtggtttacgaagtgttgtctgctggtgatt
                                   M  E  W  F  T  K  C  C  L  L  V  I    12
303  ttggccgtgtgcttcggcagtagtttcgtgcttggagacggtaggaacctacaagggtac
      L  A  V  C  F  G  S  S  F  V  L  G  D  G  R  N  L  Q  G  Y    32
363  aacggcgatctttacaatggcgaattcgagaacgaagagggtacggaagctttacggaat
      N  G  D  L  Y  N  G  E  F  E  N  E  E  G  T  E  A  L  R  N    52
423  atcatcggtcagatcatcgatgacgtcgacgctaaaaataacatccggacggccatcttg
      I  I  G  Q  I  I  D  D  V  D  A  K  N  N  I  R  T  A  I  L    72
483  gaggacacccttgagcatgcgcagtacgagccagacaagaggtccggccggtgccgctcg
      E  D  T  L  E  H  A  Q  Y  E  P  D  K  R  S  G  R  C  R  S    92
543  ggaacaaagtgtatcatgagaggtccgaacccaaacacagccagccgggttctaccattc
      G  T  K  C  I  M  R  G  P  N  P  N  T  A  S  R  V  L  P  F   112
603  ggaaagagggaggacgactcgcccaacaagctagcacggaggggacgcggaccacccaag
      G  K  R  E  D  D  S  P  N  K  L  A  R  R  G  R  G  P  P  K   132
663  aactccagagctcggggcggcaggaccctactgccttttgggaaaagacgctaactcagt
      N  S  R  A  R  G  G  R  T  L  L  P  F  G  K  R  R  *         149
723  cgattgtgaagtattttatttcccataattattttgacattatgtttttccttgaaaaca
783  acaaccaaaaactgtcctttttaaaaattaatcgcaaatattgattatgccaatcataaa
843  agttggtaagcagaaacaattctaaccatcaaggagccaattcaaaataatgttttgacc
903  aaaaccaacacagatgcttagcaaaaactgtaagcaccaagtataaaaccttacagggct
963  atggaactagtacctcctagcagatatatttacttgtaaataatatccttattggaaaac
1023 accgccctctcgtgttt
```

Fig. S1. ***A. rubens* kisspeptin (KP)-type precursor (ArKPP).** The nucleotide sequence (lowercase, 1038 bases) encoding the precursor protein (uppercase, 149 amino acid residues) is shown. The predicted signal peptide is represented in blue, two putative kisspeptin (KP)-type peptides (with cysteine (C) residues underlined) are represented in red, C-terminal glycine (G) residues that are putative substrates for amidation are represented in orange and putative dibasic/tribasic cleavage sites are represented in green. The asterisk shows the position of the stop codon. This sequence was determined from contig 1095844 and has been deposited in GenBank under accession number KT601705.

```
1                                                                   ac
3     aacagttgtcgcggttgagtgttgcaaacgaattgcgtgtaaacgctgcactgctgaagt
63    attgagcctggttcattttgctgctgcccgaagacagttgtaattgctcgaaaaacacat
123   ccgcccgttgttttaacaaagaggggcagagttccggccagaaccgttgtgagaatgagg
                                                             M  R     2
183   ccacatgtagttctattcgcttgtctagccgtgccttgcctattgctggcccagatagtc
       P  H  V  V  L  F  A  C  L  A  V  P  C  L  L  L  A  Q  I  V    22
243   agctgtgcccccgtttacgatggaaacaaccagattcagccatttgacacggacgactgg
       S  C  A  P  V  Y  D  G  N  N  Q  I  Q  P  F  D  T  D  D  W    42
303   acgggggcgacgccctcggcgaccaagactttatgatggagaccgaaaagagagacaga
       T  G  G  D  A  L  G  D  Q  D  F  M  M  E  T  E  K  R  D  R    62
363   ccgaataggcgcgaagttacatactgtatggactggatccacaacacctggagaccatgc
       P  N  R  R  E  V  T  Y  C  M  D  W  I  H  N  T  W  R  P  C    82
423   aggggaagaaaagctggctaaatgattccatcaaaaatgaatttccgaaatgatgtcacc
       R  G  R  K  A  G  *                                           88
483   tgcttttcacgactgcaagacggcgagccagttcccgaatagtctatttctaatcgacaa
543   tctcgatctaagttcattcacaaatctaattttagaaaaggggatctaataaaaaaaa
```

Fig. S2. ***A. rubens* melanin-concentrating hormone (MCH)-type precursor (ArMCHP).** The nucleotide sequence (lowercase, 600 bases) encoding the precursor protein (uppercase, 88 amino acid residues) is shown. The predicted signal peptide is represented in blue, a putative melanin-concentrating hormone (MCH)-type peptide (with cysteine (C) residues underlined) is represented in red and a putative dibasic cleavage site is represented in green. The asterisk shows the position of the stop codon. This sequence was determined from contig 1059279 and has been deposited in GenBank under accession number KT601706.

```
1                                                                  at
3    gcaaaagagtgaaaaatcactatcttattcgagtggtcggtcttcacgtttttaggttga
63   agggaggggggtgttttcaaaaagataccgcgtgctgaagtgtataaatacagcgtctct
123  gtgaagtttggttttaaattttacaccactccagtaagagaaaagtaactgcaacccacg
183  acttgaaagataatgcttcttgctatggcgccaaatggagaaatgatgaccagatttctg
                 M  L  L  A  M  A  P  N  G  E  M  M  T  R  F  L    16
243  ctagcggctcatttcttattactagcggtgtcaattgtaaatgctcgcgtgtatttcaat
      L  A  A  H  F  L  L  L  A  V  S  I  V  N  A  R  V  Y  F  N   36
303  ggcgaggacgagacaaaatcagggttgttggagctatcagagtacggggaaaatgagaaa
      G  E  D  E  T  K  S  G  L  L  E  L  S  E  Y  G  E  N  E  K   56
363  gtagacggcacggaagatgttgacggacagcaagttgaggaccgacagtggaagggagaa
      V  D  G  T  E  D  V  D  G  Q  Q  V  E  D  R  Q  W  K  G  E   76
423  gaccagtggaagtctggtttgtatgccgctcagcgtagcttgcaatcataccctaacaca
      D  Q  W  K  S  G  L  Y  A  A  Q  R  S  L  Q  S  Y  P  N  T   96
483  gccaagagatcatggccacaaactggaatgtacaacaagcagagcaccaactggcttcgt
      A  K  R  S  W  P  Q  T  G  M  Y  N  K  Q  S  T  N  W  L  R  116
543  gcactggctcaggaaccacgctggcatagtgcaatggctaagaggcagctgtgggccaat
      A  L  A  Q  E  P  R  W  H  S  A  M  A  K  R  Q  L  W  A  N  136
603  cagcagtccgggttgttcggaaaacgcgaggccgatatggaacgaactctccccgcgtgg
      Q  Q  S  G  L  F  G  K  R  E  A  D  M  E  R  T  L  P  A  W  156
663  aatgtgaagagatcggcggaggagcgagaattcgcgagacagagtcgaggtggaggggta
      N  V  K  R  S  A  E  E  R  E  F  A  R  Q  S  R  G  G  G  V  176
723  ccccatgtgtttcagagtggcggcatctttgggaaacgctcgagtgatgactgggccaag
      P  H  V  F  Q  S  G  G  I  F  G  K  R  S  S  D  D  W  A  K  196
783  agatacgaatagaatctttgaatagaatgtgagatttgttcaagctgtgacgtaacagtg
      R  Y  E  *                                                  199
843  acgtggttcgtgctgatttgggcaactcaccaggcatcgaaaggacacagtttgtaccga
903  acacaaccccagaacaccaacgcaccatgacggcaaacacaatcttaattcaatcacacg
963  ctgtcaattccttcagtcaaccttcattgatgtcatttcatcaatgtcatttcatcacta
1023 gaaattatttttgataagttattgtctaaaagtttttttaattaattatagcaattttg
1083 tcatccgttacagtggcccacataaaatagtcataaaaacatcttcttctttttttgagga
1143 gtaaattaatttacctcctccattgtactttccaacattacacgcagaaattgctacaag
1203 tatatcaattttggtaacaatgggagatttttt
```

Fig. S3. *A. rubens* tachykinin (TK)-type precursor (ArTKP). The nucleotide sequence (lowercase, 1234 bases) encoding the precursor protein (uppercase, 199 amino acid residues) is shown. The predicted signal peptide is represented in blue, two putative tachykinin (TK)-type peptides are represented in red and putative monobasic/dibasic cleavage sites are represented in green. The asterisk shows the position of the stop codon. This sequence was determined from contig 1103126 and has been deposited in GenBank under accession number KT601707.

```
1    cttaacatcagcttcatggaattgggctctagtgatcgtttgatatgtttaccctaagtg
61   aatagcctattaatatcgttttcttcttttaaatgttgtttgaagatttaaccgtgacaa
121  attgatatgcaattgaaaaaatcatcacccaatctcttggtctgtggctctctattttga
181  gatgcaagtgagcgtgcaattgtctattgatgaggagcagagtttcgttccgctccctga
241  tacagaccactgtagcaagccaacccccagctctcatcactgaaaccaaaccactgagat
301  cacccaatccattttaaaacgtgcacttcgtgccagcgtggtatttaaaggttcactcaa
361  cagttggtgccatttccgaacccagccacaggcctttgagaagagaagaggcggtcttaa
421  ctgcgcgcccatgcccacaatccagtgatcacatcttgtcaccctcttacatcaactact
481  cgatacgcgttataggaaacatctcgcgatcgctcatgtctctctccctcccccatacgg
541  cactcactcaaccacgccgtttgaccattgaaatgacgcactgacgacatcgtgggctac
601  aacagtttcttaagcagcgcgctccggaggttcacacatgggcggttacttcttctccaa
661  gcaggtccactgtacatcgactcgactggcgcggcgctaaaccatagactccattgctag
721  gaagaaacccgtggatgcagagttatttgtgagttatacttgcgctccaactcaaaaact
781  cgacaaaataataagtgaagacaagaaaaaagggggttacgtggagcgcccattcctcc
841  tcctggagtcttcttaaagggggccttggtttttagtttcgtgaaactctacttcttta
901  aacaccatgaggttctgttgtgttgtagtggttctaccgttgatctgtgtgctagctggg
              M  R  F  C  C  V  V  V  V  L  P  L  I  C  V  L  A  G    18
961  tgcctagtggcccatgcagcaccgaggagggcggaggaggcaacagcgacccgcggtgg
      C  L  V  A  H  A  A  P  R  R  G  G  G  G  N  S  D  P  R  W    38
1021 aaaaggaatttctcgccaccgggaatgcagagctcgggcggcagctacaataaaggagat
      K  R  N  F  S  P  P  G  M  Q  S  S  G  G  S  Y  N  K  G  D    58
1081 cttgttgaaaggatattgaacagattacaagaacgtctcctcgggaaagttgacctctct
      L  V  E  R  I  L  N  R  L  Q  E  R  L  L  G  K  V  D  L  S    78
1141 caaaccaacacatggcatggaaatcagagtccaaaagaattagaccttcaacgatatagt
      Q  T  N  T  W  H  G  N  Q  S  P  K  E  L  D  L  Q  R  Y  S    98
1201 gatcaagaggatgagtttatagacgatgatgatgaagtgcctaaccgcccggctattaag
      D  Q  E  D  E  F  I  D  D  D  D  E  V  P  N  R  P  A  I  K   118
1261 aggaaatgtatcggtcggtttcaacctttctctatgccctgttagagttcacccagccta
      R  K  C  I  G  R  F  Q  P  F  S  M  P  C  *                  132
1321 tcagggctgtttggtctcataagaacctcccgtacaagattatttttaagggctgtgttt
1381 tgaaattgagcgtctttggctatggctgatgacgtca
```

Fig. S4. ***A. rubens* somatostatin (SS)-type precursor (ArSSP).** The nucleotide sequence (lowercase, 1417 bases) encoding the precursor protein (uppercase, 132 amino acid residues) is shown. The predicted signal peptide is represented in blue, a putative somatostatin (SS)-type peptide (with cysteine (C) residues underlined) is represented in red and a putative dibasic cleavage site is represented in green. The asterisk shows the position of the stop codon. This sequence was determined from contig 1107850 and has been deposited in GenBank under accession number KT601708.

```
1     ctgagtcaatagtagacgtgttaccagcgatgagcgcactagactgattagatcaggggg
61    ccaggggtggcaactttgaagtacccacagtgctttgcgcgagtgggggtttataaggg
121   gtgactcttgcgctgggtccgtcacttagctttgagatcgcaaacggttaacttgacacg
181   tgtgtttatttctgcgcggacacagcacgagttcacagcaaacgagagtgtagtacgcca
241   acacccgagagatacccgttgaaacacacaatactgccgcctagagaaacaggactcgcg
301   cgcctctgcaaccaccaccgccaccacagattcaaaatgacgcagcttacgttgttggcc
                                                   M  T  Q  L  T  L  L  A      8
361   gtatgtggctctgttctcctattagttggtctcacacactgcacagacgaacaacgggag
       V  C  G  S  V  L  L  L  V  G  L  T  H  C  T  D  E  Q  R  E     28
421   aaaagattaggggacaatgacttcttccaagcaacgtacaacgacgctcaagctagacag
       K  R  L  G  D  N  D  F  F  Q  A  T  Y  N  D  A  Q  A  R  Q     48
481   aggcagagagtattgcaaagctatctggacgacaggatggcatctgtgggtaaaagagac
       R  Q  R  V  L  Q  S  Y  L  D  D  R  M  A  S  V  G  K  R  D     68
541   ggacttaaaaggaatttcgacgaagatgtttaccaccaggagggtttagacaatgaattt
       G  L  K  R  N  F  D  E  D  V  Y  H  Q  E  G  L  D  N  E  F     88
601   gtaagaagactaatggccaaatactttgatggtgttgcaagacgtcgttaaaacagagag
       V  R  R  L  M  A  K  Y  F  D  G  V  A  R  R  R  *              108
661   ttaatgcaccgctcatgaaatggatgatgaactttgaccgaacaaaatgaccactgctgt
721   ctccaagtgacctttaacctgctcgcagaacttcccctcctaaaataatcggccgactct
781   tgcggctttcatt
```

Fig. S5. ***A. rubens* pigment-dispersing factor (PDF)-type precursor (ArPDFP).** The nucleotide sequence (lowercase, 793 bases) encoding the precursor protein (uppercase, 108 amino acid residues) is shown. The predicted signal peptide is represented in blue, two putative pigment-dispersing factor (PDF)-type peptides (with cysteine (C) residues underlined) are represented in red, C-terminal glycine (G) residues that are putative substrates for amidation are represented in orange and putative dibasic/tribasic cleavage sites are represented in green. The asterisk shows the position of the stop codon. This sequence was determined from contig 1081093 and has been deposited in GenBank under accession number KT601709.

```
1                                                                  g
2      attgtatataaagtagaatcaaagtgtgcccggcatcagtttggcttaggagacatacac
62     tctgacaacacgtgagatatcatttttatggttttactttcttcggagcatcgggtcag
122    aatattctttgtataggggcaaggcgaattgggtttttaaaccaatcggagagttttgca
182    tattttgttcaggacgacattacattcaccacaagggcctttacggtaggaaaacaacag
242    aaactaaaggttgacgcagtcaccatgaacgacctacagcggcttattctgttagtgtcc
                                M  N  D  L  Q  R  L  I  L  L  V  S    12
302    cttggaacgttcgcccttctcctgtgcctccccgcctgcaccgaggcgcaacctctaggc
        L  G  T  F  A  L  L  L  C  L  P  A  C  T  E  A  Q  P  L  G    32
362    ctgtttaaatttgaatacgacgatttgttggaccccagttttgaagcggacgatcccagg
        L  F  K  F  E  Y  D  D  L  L  D  P  S  F  E  A  D  D  P  R    52
422    aatccaaggagactttcgagacagcaaatcttaaggagactgaatgatttggcaatgtca
        N  P  R  R  L  S  R  Q  Q  I  L  R  R  L  N  D  L  A  M  S    72
482    cgctcaggatcaggaccaggttacacgattccaagaaaaaggcaaggcctgtctgtttcg
        R  S  G  S  G  P  G  Y  T  I  P  R  K  R  Q  G  L  S  V  S    92
542    cccatattcccaattcagaggatccgtttgaacgcaatcgaacgggaccgtcaagaccaa
        P  I  F  P  I  Q  R  I  R  L  N  A  I  E  R  D  R  Q  D  Q   112
602    gtcgatcaggccgaggccaaccagggcctttttcaaatcgccggacgcaagagatagggc
        V  D  Q  A  E  A  N  Q  G  L  F  Q  I  A  G  R  K  R  *      130
662    aaatcataaaactaatgaccaaaataggtgtttgcaatttgtttacagatttcgtcagga
722    tattacacaggctgaattttaaaaagggtaacaacggactttttgttatacacaactcgg
782    tttgcatctttgtgttcagacgcccccataataggaccccaggcgaagcaggctttaaa
842    cagatgtgtgagttcactcaccactccacacggcagcca
```

Fig. S6. ***A. rubens* corticotropin-releasing hormone (CRH)-type precursor (ArCRHP).** The nucleotide sequence (lowercase, 880 bases) encoding the precursor protein (uppercase, 130 amino acid residues) is shown. The predicted signal peptide is represented in blue, a putative corticotropin-releasing hormone (CRH)-type peptide is represented in red, a C-terminal glycine (G) residue that is a putative substrate for amidation is represented in orange and putative dibasic/tribasic cleavage sites are represented in green. The asterisk shows the position of the stop codon. This sequence was determined from contig 1087380 and has been deposited in GenBank under accession number KT601710.

```
1                                                               g
2    catgacgtgtggtgattggtgcatgcccgacacacagtactacgatcagcagacgagatt
62   gagggacgatcgctggtttttttgcgagagtacgcgattgataacgacttcatcatcaat
122  tggctcagatacgtttattgaataacctgttgtgaagagaaaagttgtccaaggatattc
182  cacagaacttccgtccggtggacgatgggcatgaagtcaatggtggcgctgtggactggg
                                 M  G  M  K  S  M  V  A  L  W  T  G   12
242  gtactggtcgcactgtgggttcaaagtcaagcatgtttagttcaagactgtccggaagga
      V  L  V  A  L  W  V  Q  S  Q  A  C  L  V  Q  D  C  P  E  G   32
302  ggaaagaggtccagttataacacaatcagacagtgcctatcctgcggccctggtggtttg
      G  K  R  S  S  Y  N  T  I  R  Q  C  L  S  C  G  P  G  G  L   52
362  ggacaatgtgtaggttcagctatatgctgcggcaatactttcggctgttttctcgggaca
      G  Q  C  V  G  S  A  I  C  C  G  N  T  F  G  C  F  L  G  T   72
422  aaagaaaccttcgtgtgcagagaagaaagtcagctctccacaccttgtgaggttgttgga
      K  E  T  F  V  C  R  E  E  S  Q  L  S  T  P  C  E  V  V  G   92
482  gagacatgtgaatctattactgacgggaaatgtgtttcaaacggcttctgttgcaatgag
      E  T  C  E  S  I  T  D  G  K  C  V  S  N  G  F  C  C  N  E  112
542  agaagctgctctttagacgtagcgtgcagagaaaccgatacagaacagagagacctcaaa
      R  S  C  S  L  D  V  A  C  R  E  T  D  T  E  Q  R  D  L  K  132
602  aacagactcaaagagaggcttctggacgccctcttgcgtcaaccatgaacccagttacca
      N  R  L  K  E  R  L  L  D  A  L  L  R  Q  P  *                147
662  ctccctcaactttgttgataattttcacccacaatgcaatgtgatatggtcacttgtgac
722  accttcacggacacaatgttgcggtcctttatgttgtgacaatcttggtgaatttctgaa
782  tatgctcacgtatagacacaggacaccatacttttataggggcaccagaccattattata
842  gccaggctcggtttggtcacaaaacaaacccctatttttgtggatgataataatctttgg
902  gacacgtaactaactgtatcacgctgcattgtaggttaagagaagctttgaagatatcct
962  cattaattttggtattttattcagattgctttcttagaaatgttaattaaaccgtttggt
1022 attctttgaaattaatcgtaaatttaaaaactatttaaatacacagcggtaccgacattg
1082 ggactaaagagcaatttagtttgtgtacatctttctgatgcaagtaaacggaacttcaaa
1142 tggggaaagatcagataaacaaacaggggacggatgcagttacttttaatgagacaaata
1202 aaatgtgctcttaccaaaaaataaattacaattttcagtttctttaaaaaaaacatggtg
1262 ttgttaatgtcttaatgctttctagcacttacaaataatattacacaattttttgggtgcg
1322 ctaaactttgagcttttgacaagattctttcttgcattgcgtcatattttattatctttc
1382 gaaatgtcaggatcacagttgcaattatagccatatcgatgttcatcttgagcgaaataa
1442 acagcaaaaacaatatttattttctgaatatatagttgacagtcgttcacttttaatata
1502 tatatatttagattcaaattctcagcaattttagccaatattatagtacaataatgtgac
1562 tgctacgacagactttaaaaataaaaaaaatgtaaataaaaatgttattttagatttagt
1622 taaattaacttttgaaaatcagctgcaaaactgcacgcaaatggccccaaaacaaacaat
1682 aaatcaaatatatgttaacaagagcacactcttgtggaattttagaggggacaaaccggc
1742 tgtatgagacaggtgaactttctataagaagtccacactttaaaatttataatttaatct
1802 ttagagtacccagtaaaacaacacattttctgtcgcttttttattgtttaacgtcccatag
1862 aaataattgcacatgtagatttaacttagcagactttgactatacgatacttttcgactt
1922 tactgcgcaactcgtacatttcctgcgtataatagatacaattttgacctattttaaaag
1982 ttcaatgtgaatttcttgttgtttcccaaaaatatgcagtcattctgtgtatcataatat
2042 tgatgtgaagtggtttcattttagttgaattaaaaccccactgaacgaaaattatttcgt
2102 tacgaattgtacattgttaaaagctctttctccatggttaacccgtgtccatagtaggcg
2162 gtcgggatgtgaatgccgatttatgttgaactattggggggggggggg
```

Fig. S7. ***A. rubens*** **vasopressin (VP)/oxytocin (OT)-type (asterotocin) precursor.** The nucleotide sequence (lowercase, 2209 bases) encoding the precursor protein (uppercase, 147 amino acid residues) is shown. The predicted signal peptide is represented in blue, putative asterotocin peptide is represented in red, a C-terminal glycine (G) residue that is a putative substrate for amidation is represented in orange and a putative dibasic cleavage site is represented in green. The C-terminal region of the precursor comprises a neurophysin domain, with fourteen cysteine (C) residues that are a characteristic and conserved feature of neurophysins, which is underlined. The asterisk shows the position of the stop codon. This sequence was determined from contig 1119045 and has been deposited in GenBank under accession number KT601711.

```
1     ctacacgcagtgattgcacggtaatgcagcgtgacgtagccacgaggaggcgtaactttc
61    tcgttgcgaacagactactagcgcaccggggctgtgcgattattgtttccaacacgaggt
121   atttcatagattggcgacaacggacaagcaaagaagaccttataggcttagagaggacca
181   tcgagaagagcttgagttactttacctggcgctcaggtgggaattcattttctatcagca
241   agaacactccttagtttacaatcaattacaagtggaatatcgctcatttggaaacatcaa
301   caagattttgacgaactaggaggggtgtcggtgggacgtggggatctaagctggatatg
                                                             M     1
361   accatgggcagcaggtcgttattagtgacaattgtgatcacagtagtcatacccagcatc
       T  M  G  S  R  S  L  L  V  T  I  V  I  T  V  V  I  P  S  I  21
421   tggcaggtgcaatagctggggctcaaacacaaaagattcgtcgtgaaagtcgagaatct
       W  A  G  A  I  A  G  A  Q  T  Q  K  I  R  R  E  S  R  E  S  41
481   ggcaagtactggccaaactccgtgggtatctcagaccaacagctacggcaactcctagca
       G  K  Y  W  P  N  S  V  G  I  S  D  Q  Q  L  R  Q  L  L  A  61
541   cactctctggcggactcgtacagtacgtcaggggcaagtcacatacggggaggagacggg
       H  S  L  A  D  S  Y  S  T  S  G  A  S  H  I  R  G  G  D  G  81
601   gatgcagggtatatatacgatagtcgagatcaggtcgatgacacggggacgaacgaggag
       D  A  G  Y  I  Y  D  S  R  D  Q  V  D  D  T  G  T  N  E  E 101
661   gaaggggaacgcgtaatcgggagcgaggttacatcgagagactcgaaccccggtacaagc
       E  G  E  R  V  I  G  S  E  V  T  S  R  D  S  N  P  G  T  S 121
721   aagagaaatgggttcttctatggcaaaagaaatgggttcttttatggaaagagatcagcg
       K  R  N  G  F  F  Y  G  K  R  N  G  F  F  Y  G  K  R  S  A 141
781   tcaacccctggcaatgcaaatgaagtaactcaatgcatcccgtgtgggcctcaaaacaac
       S  T  P  G  N  A  N  E  V  T  Q  C  I  P  C  G  P  Q  N  N 161
841   ggccagtgcgtcatgtttggtacatgttgcagctatgaactaggtggctgcttttcctg
       G  Q  C  V  M  F  G  T  C  C  S  Y  E  L  G  G  C  F  F  L 181
901   acagaggaggcccttccctgtgtgacgtcaaaatcgtcatcattatgtgagctgagcgga
       T  E  E  A  L  P  C  V  T  S  K  S  S  S  L  C  E  L  S  G 201
961   ttgccgtgcggtgacgagggatatggaaggtgcgtggcagactctgtctgttgtctgccg
       L  P  C  G  D  E  G  Y  G  R  C  V  A  D  S  V  C  C  L  P 221
1021  caagagggctcttgtcatattaacgcagaatgtggaggcaagatgacatttcaataggac
       Q  E  G  S  C  H  I  N  A  E  C  G  G  K  M  T  F  Q  *    239
1081  ttgcattatgcggactttaaattatttataaagggataggaaaaggtggttaatatctgt
1141  atttgaaaagggtaataaaatttaaggttgtttgagaaaagggacacgaatgttatttt
1201  gacctcaatgtgtaaatttaaacaattttagcgattacttatttttagaccactacgaat
1261  taactgtt
```

Fig. S8. ***A. rubens* NGFFYamide precursor.** The nucleotide sequence (lowercase, 1268 bases) encoding the precursor protein (uppercase, 239 amino acid residues) is shown. The predicted signal peptide is represented in blue, two tandem copies of the putative NGFFYamide peptide are represented in red, C-terminal glycine (G) residues that are putative substrates for amidation are represented in orange and putative dibasic cleavage sites are represented in green. The C-terminal region of the precursor comprises a neurophysin domain, with fourteen cysteine (C) residues that are a characteristic and conserved feature of neurophysins, which is underlined. The asterisk shows the position of the stop codon. This sequence was determined from contig 1104160 and has been deposited in GenBank under accession number KC977457.

```
1    aaaaactgatttgcttaagctggaagtaacaaaatattgatcgattgtcttgaaagatga
61   acctgtcccagttcattcttcggagacattactctgtattaagatcaatccacgtgtgat
121  gaaggttacggatacgctgagacgtcaattgatgtcactggagtcgacaaatgcgtgcac
181  gccgattagttgctagactttttagccaatggcgcactgttgctcagctcacgtgataat
241  accgtcaactgtcccttttcactcatttcggttccttgttttaaagcatcagacgtgac
301  ctcttgagattgatcaaaaagagctttatatgttcagtgtcaactcattatttcagcaga
361  taaagaccgtcagagtgatttttcacacagtatttacgtttatagtcatcgttaatagtt
421  cacgtcacgaccactgggctacattaacacccatctatagatccgcgtaacactcccaag
481  cccagctatctgttataccggtaaccattaggtggagattgcctgccatacatttatgcg
541  cacgcgacacagcgtgcaagtctcagttcgttctcagttagcaaccaagaaatagtgtaa
601  gcgcttcataaggaaaactgtaagagaagaacacaggagagtcactggagttaagaagcc
661  caagtcaccttataaggtaattttgtacagatggccgatatgaggatgttaacactcact
                                     M  A  D  M  R  M  L  T  L  T    10
721  agcgtattagtctctctactcttcatggcagaaattcaaagatgccaagggcagatacat
      S  V  L  V  S  L  L  F  M  A  E  I  Q  R  C  Q  G  Q  I  H    30
781  tacaagaatcctggatggggacctggtggtaaaaggagttcacacatgactggtagcaat
      Y  K  N  P  G  W  G  P  G  G  K  R  S  S  H  M  T  G  S  N    50
841  gtattaaggaaacggcattggcgcgtggaatctgatcagatgggtacagacagcatgcag
      V  L  R  K  R  H  W  R  V  E  S  D  Q  M  G  T  D  S  M  Q    70
901  aaagaacgaaacttgatcatgcttcaagaaattgcaaaatctttggcaaagcaactggta
      K  E  R  N  L  I  M  L  Q  E  I  A  K  S  L  A  K  Q  L  V    90
961  gtaccaacgagtgaggacgacacagtcctggaccaattaacggtcgaccaatggcggcag
      V  P  T  S  E  D  D  T  V  L  D  Q  L  T  V  D  Q  W  R  Q   110
1021 gaagcagacgagataaatgacaacggttggaattaagcgggaaaagctctgaaatttgac
      E  A  D  E  I  N  D  N  G  W  N  *                           121
1081 aacaattatttagaatcaggaagaactgaacaacttgatacaggttcatatgtgtttgta
1141 ttgctttctttttt
```

Fig. S9. ***A. rubens* gonadotropin-releasing hormone (GnRH)-type peptide 1 precursor (ArGnRH1P).** The nucleotide sequence (lowercase, 1154 bases) encoding the precursor protein (uppercase, 121 amino acid residues) is shown. The predicted signal peptide is represented in blue, a putative gonadotropin-releasing hormone (GnRH)-type peptide is represented in red, a C-terminal glycine (G) residue that is a putative substrate for amidation is represented in orange and a putative dibasic cleavage site is represented in green. The asterisk shows the position of the stop codon. This sequence was determined from contig 1100532 and has been deposited in GenBank under accession number KT601712.

```
1                                                                g
2     gactccggacatcatctgtagttgatctatcaatcgcgttcgaagacgatggggagttac
                                                   M  G  S  Y    4
62    tcggttacggccaccatatacctagccctagttttaggttctttagtatgtagcgcccac
       S  V  T  A  T  I  Y  L  A  L  V  L  G  S  L  V  C  S  A  H   24
122   aatacgttcaccatgggtggacagaacaggtggaaagcaggggaaagagatcggctccc
       N  T  F  T  M  G  G  Q  N  R  W  K  A  G  G  K  R  S  A  P   44
182   gcagggcgccctcaacaaactttcttggacccatcaagcttcagtgaagatcagcaagga
       A  G  R  P  Q  Q  T  F  L  D  P  S  S  F  S  E  D  Q  Q  G   64
242   gaaacgacaattacgctacgggagatgctggtcgacatgagagactactgcagtttcctc
       E  T  T  I  T  L  R  E  M  L  V  D  M  R  D  Y  C  S  F  L   84
302   ttgaagctacttgacaacgttcggctgcctcaaaccgaaagaaaatgacacttctaagat
       L  K  L  L  D  N  V  R  L  P  Q  T  E  R  K  *               99
362   gaaatcagacgccagactactataccacgtctatctttgtctatcttgcgaaatggagga
422   ggctgtgcccagacatgaattgcaggaccttattaccagggagaccgtgatgggcagttc
482   cgattctctactttagagtagtgacgtcattccgcaggataaaaaatgatggatgaaaat
542   cgactgagagggaccacccttgtgcttgggggtttactctccctcaacacagcgactat
602   gaaattaaacgagacaactgaaaagtgaaaacgacaaacttgtatattttcagaatagta
662   catcagccccttttcgattgtgttttggggtgtgtacgcgggcgcctttattctccaacg
722   tataaaaacctcctttgaccacacagctactaacaattgacattgctatcattcaattat
782   tcccgccgatattttcgacggtcctttttttctttcttcaaaacacccgtaattttttttt
842   gaacacgtggggattgaagcgtttttgagaaaacagttttctaaaaataaaccgatacga
902   ttaatttcccagccatttgacgtttaaaaacaagaaggccctaccatactgcgtacattt
962   ttgatgttccctgttcagttcatccttttaaaattttacaaattactctatgaaaaccta
1022  caaaccactagttagattctaaaagcttattgcttttggaattctagtcccataagaaag
1082  caaaatattcgtgttaagcatacaaatgtgcttaggcgtaagcgtatttttacaggttaa
1142  caagacaattgagcggccagctattgcagccaaggtttaccatcaataagcaccggaagg
1202  ttagcatgctttaagagatgaaattccccttaataataataataattattacgagtccaa
1262  tataaggtgtaaaccacgttcagcaaaggtcaatgt
```

Fig. S10. ***A. rubens* gonadotropin-releasing hormone (GnRH)-type peptide 2 precursor (ArGnRH2P).** The nucleotide sequence (lowercase, 1297 bases) encoding the precursor protein (uppercase, 99 amino acid residues) is shown. The predicted signal peptide is represented in blue, a putative gonadotropin-releasing hormone (GnRH)-type peptide is represented in red, a C-terminal glycine (G) residue that is a putative substrate for amidation is represented in orange and a putative dibasic cleavage site is represented in green. The asterisk shows the position of the stop codon. This sequence was determined from contig 1104992 and has been deposited in GenBank under accession number KT601713.

```
1    aaaaaacatacacacatagaaaagaacgggcattctcaacttgctgtagatcacagccgt
61   aataagtgactggctataacaaccgatacaatttatagggactagccaatcaaagctcct
121  cactttgcggtggtacgctgtgtgtggtgcaagtgataagacagcgtgctgcgatacggt
181  gcttgacagtaaagcgagaaacacgctagcgaacgggacacgatacttttatttctctc
241  tctcttactctctatttctctctgatgaacaagcagtcaaccatcatcgagatcatgctt
301  atgcgtgtctgattacacattgtgcttgttcttaattttgtcctgcagagttgttactt
361  caagcagtcaaatctcttaagttgtgccggccgacaccaagcaaccgataaagccaagtc
421  actccacggcaagggacgagcaccctgttatttgacccgggccggtaggccgttagcgt
481  ccagtcactataatcatgtcggttacgagaaacagcggttttctacttgttactctactg
                         M  S  V  T  R  N  S  G  F  L  L  V  T  L  L   15
541  ttcacttgggtggtttgtcgcgctgagttggcagatttcgtggagaatgctgaggtggcg
      F  T  W  V  V  C  R  A  E  L  A  D  F  V  E  N  A  E  V  A   35
601  aaagaagtatctaatgaaattgaaggagtagaagcagagcaatggcagagggacgaagac
      K  E  V  S  N  E  I  E  G  V  E  A  E  Q  W  Q  R  D  E  D   55
661  aaaagacaatacccaggagggctcccattggtctagatggtaaaagacaatggtacacc
      K  R  Q  Y  P  G  G  A  P  I  G  L  D  G  K  R  Q  W  Y  T   75
721  ggcaagcggcaatggtatacgggtaaacgggacgctgaagactcgcccgcattattagca
      G  K  R  Q  W  Y  T  G  K  R  D  A  E  D  S  P  A  L  L  A   95
781  gaaaacgataaacgccaatggtacactggcaaacggagcggcaacgaagaacagcagccc
      E  N  D  K  R  Q  W  Y  T  G  K  R  S  G  N  E  E  Q  Q  P  115
841  gacgaggcgaacaagagacagtggtataccggcaaacggcaatggtacaccggcaagcga
      D  E  A  N  K  R  Q  W  Y  T  G  K  R  Q  W  Y  T  G  K  R  135
901  ggggacgaagacagggtgctggacgacgatgccgtgaactctctcaaacgccagtggtac
      G  D  E  D  R  V  L  D  D  D  A  V  N  S  L  K  R  Q  W  Y  155
961  accggaaaacgccagtggtacaccggtaagcgaagcggggtcgagcaagccgacgacgga
      T  G  K  R  Q  W  Y  T  G  K  R  S  G  V  E  Q  A  D  D  G  175
1021 gacttggagcaacaatacaacaaacggcaatggtataccggtaaaagggccgacgacctt
      D  L  E  Q  Q  Y  N  K  R  Q  W  Y  T  G  K  R  A  D  D  L  195
1081 gctgatgctgctgacctagaaaagcggcagtggtacaccgggaaaaggcagtggtacacc
      A  D  A  A  D  L  E  K  R  Q  W  Y  T  G  K  R  Q  W  Y  T  215
1141 ggtaaaagacaatggtataccggtagacgttaatcaaccaccaccactccttaatgaact
      G  K  R  Q  W  Y  T  G  R  R  *                             225
1201 gtcaaaatatttcctggatattgcggggctattaaagactttttgttttcaaacacaaac
1261 ggttgacgaatccctccccgaatccagttgcattattgaatggaagaaacggtgtaagat
1321 aacattaaaataagtttcaagttttgcataaataatttatggatttttttgttttgttttc
1381 tcgaacaaagaccgaagtcacactatagtcaaaatattattttcactacctttttctgaag
1441 gttcaaagcaatgaaaacattttaggcctagcacatttgttttgaaacgataaggtaact
1501 catgatcttcgtaaggtgacactgacaaaacgaaatccaccccatatttttttaaatttaa
1561 taattaacgattaatacggaagccccaaactgccattcaagatatacgtatatcgtatag
1621 taatacagaagtttggtatagtttatctatatgttcttcaatttcttacatgacgaacgt
1681 tttgacgtcactgttacgtcactacggcgagggctgtcccagtgacgatgataaggtgtc
1741 acgaatattgtcggtgaccgtttacaagttcaatgtctgtgtttatggcgagattgtgtc
1801 gtaaagcatattttaaataacatggccgctccgaggaaactcattaaaaaaaatgatgtt
1861 ttcaaatcttgatttcttgtatctaagaacttgttattattcagggctacagtggccaat
1921 cccgatgaaaacaatacaacgaccacatt
```

Fig. S11. ***A. rubens* thyrotropin-releasing hormone (TRH)-type precursor (ArTRHP).** The nucleotide sequence (lowercase, 1949 bases) encoding the precursor protein (uppercase, 225 amino acid residues) is shown. The predicted signal peptide is represented in blue, twelve putative thyrotropin-releasing hormone (TRH)-type peptides are represented in red, C-terminal glycine (G) residues that are putative substrates for amidation are represented in orange and putative dibasic cleavage sites are represented in green. The asterisk shows the position of the stop codon. This sequence was determined from contigs 1062625 [1-42] and 1105818 [43-225] and has been deposited in GenBank under accession number KT601714.

```
1                                                              gt
3    aaaaatagcgcggggaaagtacgaaacagcgattgcctttttacagcccggagaaaaggg
63   atcaagaccgcaagacaaacaaaaggagtttcaggcaaaggcaagggaagagatctatat
123  aatacaatcatgaagcctacaacagttttgacgctagccgtcttctgcaccctttacacc
               M  K  P  T  T  V  L  T  L  A  V  F  C  T  L  Y  T   17
183  atcatcacagccgcctcgatatccagggatgatgacatgtttgacgtgacgggagacgat
      I  I  T  A  A  S  I  S  R  D  D  D  M  F  D  V  T  G  D  D   37
243  ctgaggcaattagcaaagaaagtagacacatacgcaagaaacaacgaaatacagtcacta
      L  R  Q  L  A  K  K  V  D  T  Y  A  R  N  N  E  I  Q  S  L   57
303  ctaaaaagaaatggggaatctcgtggctgttcaggattcggcggctgtggggttctgact
      L  K  R  N  G  E  S  R  G  C  S  G  F  G  G  C  G  V  L  T   77
363  attggtcacaacgccgccatgcgcatgctcgctgaatccaactcgcccttcggtgccagt
      I  G  H  N  A  A  M  R  M  L  A  E  S  N  S  P  F  G  A  S   97
423  ggtccaggcaagagaaggagatcggttgacgctgtagcaaaccaagaggcgtagaaagga
      G  P  G  K  R  R  R  S  V  D  A  V  A  N  Q  E  A  *        114
483  ttgaacaacgggagtagtgaaacaagaaaacatcaaagcgtactttaagcaactgctttg
543  cttctcaactgaacaacgccaaagattctacacagacgacgtatttttttgtcagctga
603  ttatgagtgttccggattttcaaacgtcatt
```

Fig. S12. ***A. rubens* calcitonin (CT)-type precursor (ArCTP).** The nucleotide sequence (lowercase, 633 bases) encoding the precursor protein (uppercase, 114 amino acid residues) is shown. The predicted signal peptide is represented in blue, a putative calcitonin (CT)-type peptide (with cysteine (C) residues underlined) is represented in red, C-terminal glycine (G) residues that are putative substrates for amidation are represented in orange and putative dibasic cleavage sites are represented in green. The asterisk shows the position of the stop codon. This sequence was determined from contig 1064235 and has been deposited in GenBank under accession number KT601715.

```
1                                                                cg
3    ggcttcaaacttcaacatctcttgttatgctcgtcagctatcgtggttctaaaatcgcat
63   actcttacaacgttccggctgcttacatctctccccaattccgtctgcatctaaccaaca
123  aagggacctcctctgttaatttgttcactttttttgatcgaattgatctgattttcagttt
183  tgattcccactaggaacacgaacttgacgtgtttaaaacgaactgtcggtattttggttc
243  cttcttgctgagctgcaaggggccacatcgctactgtttctctcgcattgcttgtatttt
303  gactgattgaacttatcaacacctatccgtatataggaagttctgctcgagatcataatg
                                                              M    1
363  agtagttggcttacagtcgccatagcaactgtgacatgcctttttgctttcgccaatcacg
      S  S  W  L  T  V  A  I  A  T  V  T  C  L  L  L  S  P  I  T   21
423  tgcctgcctcttcatgacgtagccgacggtaaggaaaggcgggaactcctgcacagcacg
      C  L  P  L  H  D  V  A  D  G  K  E  R  R  E  L  L  H  S  T   41
483  tggttagacccgtccggttcaacaggtcaaggcacggaggaattggccgagacgagcaag
      W  L  D  P  S  G  S  T  G  Q  G  T  E  E  L  A  E  T  S  K   61
543  cgactacttggggataacaacagggactcgggcattattgacctccttgtagcactgcga
      R  L  L  G  D  N  N  R  D  S  G  I  I  D  L  L  V  A  L  R   81
603  gacacaaacacaaacccgagagatctttatcttcacggcaacacagagacagctcgtaaa
      D  T  N  T  N  P  R  D  L  Y  L  H  G  N  T  E  T  A  R  K  101
663  cgaagacaatccaaggtggatgactacggccatggtctattctggggcaagagaggatcc
      R  R  Q  S  K  V  D  D  Y  G  H  G  L  F  W  G  K  R  G  S  121
723  aactggtcagaccacggggtacgagcaatgacagataaggacaccaagaggggcggtgat
      N  W  S  D  H  G  V  R  A  M  T  D  K  D  T  K  R  G  G  D  141
783  gatcaatatggctttggcttatttttggcaagcgaaatgaagaagactacgaagacttt
      D  Q  Y  G  F  G  L  F  F  G  K  R  N  E  E  D  Y  E  D  F  161
843  acgttgtagattgtttagcaaataaggatacttaaaagctctaaagaatttggcaaagta
      T  L  *                                                     163
903  atgttttgtaaataggcatagtctttatgataagttaacgcaatatcaaactaatataac
963  tcgacacttcgttggggttaagtctcaatagagtgtccaactatcggccaaatacaatt
1023 gtgcaaaaaacgtctcgaaatcatttacaaataattcacacaaaccatgggtacttggta
1083 attgtgtaatgcgtttgaacgtgcgatgtatcgagttaattagactgataaatacggata
1143 aatcatgtaaagccagtacagctcacactgcaaattacaacattagctttagtttaagtt
1203 taaagattgacttgtcatgttccgagcaaaggttgccatcatttgatttttttttaatttg
1263 ataggaatcattgttcaaatgacgtttaccgcaagtggtattacacctctctttctgaac
1323 tacatgacaggatcaaaatgacagtttagcagaagtattttgtgttatgcggttttataa
1383 atataaaccccatgtttaagaaaccttgtattttagaacgcaagcagttgttgacgcctt
1443 ttggaagtcttgcaatctgtaaatgtatgtgggtaccattgaagctatatgccatctcaa
1503 tcactgcttctctatcataacctttttggaaaaacacacattttataggcaaaatagtaa
1563 agttatgagttaaccacaacgttgcgttgcaaatcggagcgctttcaatccgtcgatgtt
1623 ttattggtttgaactgactgttgtttctcaataataacaaatctgtgcaactactgtgga
1683 tttcgttgcgtcattctgaatattgtttcttttgtttaaactccaatgttgacaaactta
1743 acgttaatccattgtaaattattttatgcataaccgtcttttgagtcaacttcgtaagcc
1803 cgatgttttattttttgttaagtgtatataccttgaacatgaactagcagaaaagagttac
1863 ttagcacgatttgcagatttgaatcatgtaggaagtataacacatttatatagttctcaa
1923 caatgctttactaatctatctaaggaaagttgccgttctgaaaagaagcaggcttgagat
1983 ggaggtatttcgtttcctactcacaacctctccttcttgttgtacagattaatataacat
2043 agttgtcaacctacattattaaaccgatcgtgtaatttgattggcggaacacgcgtcacg
2103 tgtcatactttacagtattttgccaagtaaactgctccaacactttatatttatttagaa
2163 gagttctatattattaaatattgtacgcttttgacgtttcgtaacttcgctttgagattt
2223 acattgagaatcgtttgcattgattgcgctccaggacaggttaggctatggacgccgaac
2283 gtgctttaaatagcatacttgtttatacacagctttatcgatcatttcatacagtaatgt
2343 cgaaataggtcatatagtgttcaaaacagtgaattgaatttataaataatgtcaattgac
2403 aaggatttatccatgggcactcacatatatactgcattttattcataataaagcagatca
2463 caatccctagttgaaatctgaactgttttatttaacttgagctcgtgattctaatatgac
2523 actctttacgcatcacacaatatttgtatcgtagcccctgacgcaaatgaatacaagtgt
2583 cttatgtattatgcaaattatagttgagttgttatccaaagattagttaacattattttg
2643 tcctatatcattagcgctaaaatgtaacatttactttgaagtgataaaacgtgtctctat
2703 tcctttctgactgagcacttgggtagatacagtgcgcttaaatttcaacgtgctgactat
2763 gacattaaacaataacgacttgcttaaattaagttttcaggtgataaatgcatcaccaaa
```

```
2823 tgttggatatcattacaaagtctattaaattccgaaaccctaaatgtcaatttttgtgaa
2883 caaattagttccaaaagttgtaacagaccaggtttgccattgtggcaacagttgtcaact
2943 tttacggatacagtggtcattgtgttgtttactaaaatgtttgaagtctcgaagcaaaag
3003 ctggaataaaaaagtttaaaaaacgattcataaaaaacatttatactgtatatttattca
3063 ttttaactgttttgttgcgttgaagctaagtgtgttgcttatcaattgaaaacagtaaca
3123 gtacaatgaaacatccagcaaaccaaaaatcgacagtaaaccaaaaatcgaaacaaacga
3183 gcaaacaaacaaacaatgaaaaaaacaaaatcaagtctaaggcaaaagttttcaatcaaa
3243 ttaacgaaggtgatatggcctaaattaagtaagcgattttaacgaacattgaaaacgaac
3303 tttactcttcctaacttcaaatcgaaactaaccaataagaacaataaattataacataat
3363 atcccttaaatttactggacacaaccatttactagtaaagacactggacgcttttggtaa
3423 ttgtcaaagacc
```

Fig. S13. ***A. rubens*** **cholecystokinin (CCK)-type precursor (ArCCKP).** The nucleotide sequence (lowercase, 3434 bases) encoding the precursor protein (uppercase, 163 amino acid residues) is shown. The predicted signal peptide is represented in blue, two putative cholecystokinin (CCK)-type peptides are represented in red and putative dibasic cleavage sites are represented in green. The asterisk shows the position of the stop codon. This sequence was determined from contig 1124413 and has been deposited in GenBank under accession number KT601716.

```
1    gcaaggtaaactacgctgagggtctttattagatgctgatcgtaggacgtgacgtacaca
61   acctccactgcctactactactccttctacccctggaccccaccaacccacaaaatcggc
121  ccccgcatcatcagacgcgagcaaagtgagcaagcaatagctttaaagacttgggatcaa
181  ggcaggcggaattagtcagtcgcattttgtacgctgtaaaagtaacctgggatagagaga
241  ttgctgcttaacaacccggaggaggaagaggcgtccaatagcaccaagtgtagagaaaca
301  tcacagccgggagagagtggagggctcaatcggtgggagagatcggatcattttgtggga
361  ctgtctactgcgactacacctggtacatttatacaggtcttacggacagggaacttgatt
421  gtaacaaagttggatacgagtagagtcggcacaacgaggaaggtggtcgtacaggtgtac
481  aggatgatgacgcggtcctacctggtactctgctgtctggtaatctgggcgtttctagca
         M  M  T  R  S  Y  L  V  L  C  C  L  V  I  W  A  F  L  A   19
541  acgggcacgttaccaagcaacgcagacagcgcctgctgcgctcgaaccttcaggtgtaat
      T  G  T  L  P  S  N  A  D  S  A  C  C  A  R  T  F  R  C  N   39
601  ttacggagcgattgcacttgtatggtcagagagatcctctgcagggatccctccgagggg
      L  R  S  D  C  T  C  M  V  R  E  I  L  C  R  D  P  S  E  G   59
661  atgttaaacagcggaaaacgatcacccccgtcggataggcagcaacaacaactaaacgta
      M  L  N  S  G  K  R  S  P  P  S  D  R  Q  Q  Q  Q  L  N  V   79
721  ttggagagtagtggttaccatgccaacccaaggatgatgccctcatcgtcaaagtaccgg
      L  E  S  S  G  Y  H  A  N  P  R  M  M  P  S  S  S  K  Y  R   99
781  agagcacttccactggcaacaatgaatgaggacttcttgtgatgacgattatgaaacatt
      R  A  L  P  L  A  T  M  N  E  D  F  L  *                    112
841  accaacgaaaaagaaaaaaa
```

Fig. S14. ***A. rubens* orexin (OX)-type precursor 1 (ArOXP1).** The nucleotide sequence (lowercase, 860 bases) encoding the precursor protein (uppercase, 112 amino acid residues) is shown. The predicted signal peptide is represented in blue, a putative orexin (OX)-type peptide (with cysteine (C) residues underlined) is represented in red, a C-terminal glycine (G) residue that is a putative substrate for amidation is represented in orange and a putative dibasic cleavage site is represented in green. The asterisk shows the position of the stop codon. This sequence was determined from contig 1086098 and has been deposited in GenBank under accession number KT601717.

```
1                                                                     gg
3       agctacaggatgcggacgaccactcttttcgtaatacagacagtagttgttatcgggtac
                 M  R  T  T  T  L  F  V  I  Q  T  V  V  V  I  G  Y    17
63      ttcacctgttcgtcgacggcagcggccaatgcctgctgcaggggaacgtgtcatgacatc
         F  T  C  S  S  T  A  A  A  N  A  C  C  R  G  T  C  H  D  I    37
123     ccaccaggttgcaactgcccgtataagtcttacttgtgcggggagttgaacgcacttaca
         P  P  G  C  N  C  P  Y  K  S  Y  L  C  G  E  L  N  A  L  T    57
183     atgggaaaacgaaaagcagacgacacttcttatctcctgacgcaagaacaggagacgcaa
         M  G  K  R  K  A  D  D  T  S  Y  L  L  T  Q  E  Q  E  T  Q    77
243     caacaaaaccagcagcgaagaacgcaacagacccaaccctgggttgaccggcaaccagat
         Q  Q  N  Q  Q  R  R  T  Q  Q  T  Q  P  W  V  D  R  Q  P  D    97
303     gacgaccggatcgtagatgttttgaataatcttttgaaactcttcaaggaaactcatcaa
         D  D  R  I  V  D  V  L  N  N  L  L  K  L  F  K  E  T  H  Q   117
363     ggggatcaagatggtttcgatctccaagaccagagtgatgactgggaaccagtgacgtca
         G  D  Q  D  G  F  D  L  Q  D  Q  S  D  D  W  E  P  V  T  S   137
423     agtcgaaagcaacaatccgagaatgcgcacaacgtgtaccgccaccaaccgcttttctca
         S  R  K  Q  Q  S  E  N  A  H  N  V  Y  R  H  Q  P  L  F  S   157
483     gcggacatattgtagaatagacgagcgaaatttaaattttatttctttaatcctgaaa
         A  D  I  L  *                                                161
543     acaaaactactttaaaaaaataatgccgtacgtatttttgcagtcttttaaggcttactt
603     tttaaagtttatttcattgtcagacattccttctgtcgacttctcctcaatctgaaatgc
663     ttttcaaaacaattgcataatttcatccatgctttttgcaccacttcaaatttcctgtga
723     acgtgacggttttcataatgtttaaatggcaacaataaactcaaaaatgattccgtggtt
783     tttgtttttttgttttttttaa
```

Fig. S15. ***A. rubens* orexin (OX)-type precursor 2 (ArOXP2).** The nucleotide sequence (lowercase, 802 bases) encoding the precursor protein (uppercase, 161 amino acid residues) is shown. The predicted signal peptide is represented in blue, a putative orexin (OX)-type peptide (with cysteine (C) residues underlined) is represented in red, a C-terminal glycine (G) residue that is a putative substrate for amidation is represented in orange and a putative dibasic cleavage site is represented in green. The asterisk shows the position of the stop codon. This sequence was determined from contig 1081859 and has been deposited in GenBank under accession number KT601718.

```
1                                                                 tt
3      ttagttgctgcccggaaggctgtcccgcgctccgcagttcggcaagagcaaagcatcgcg
63     gcacggtcagacattggaacccaacgtgtcagaaggaacggtcgtgtttttttagtcggtg
123    tgaaggctctgctataatagttagttataaacggggaaagaacagagacaccatcaattc
183    agcagcaaccaacttcgtttttgtaataccttgaacgaaagaacacgagctgaggaacgc
243    tttgaacagttttatttttttctgaaaagttatttaatcttggagagaggaccacagtga
303    gagttgcttgctattatgacgaacagaacggctcaggagcaatgcacaagggcaggcagc
                       M  T  N  R  T  A  Q  E  Q  C  T  R  A  G  S    15
363    atttgtccaagcatcatccgcttcagcgcttggctcttattgacgatactggtagcacaa
        I  C  P  S  I  I  R  F  S  A  W  L  L  L  T  I  L  V  A  Q    35
423    gttctcctgggtaccacagccaaggcagaagagaaaactaggttccccaaattcatgaga
        V  L  L  G  T  T  A  K  A  E  E  K  T  R  F  P  K  F  M  R    55
483    tggggtaaaagatacagcccagattacgtggttatggacgacaatgaactaaaagacgag
        W  G  K  R  Y  S  P  D  Y  V  V  M  D  D  N  E  L  K  D  E    75
543    atgaaattgcccgtatttggtaacggtgaagttttgtgcaaaaacgtggcgtccggcggc
        M  K  L  P  V  F  G  N  G  E  V  L  C  K  N  V  A  S  G  G    95
603    ctttaccgttgtggcaaagtaccagcgaccgcatgagaaccgttgaagatactaaaccga
        L  Y  R  C  G  K  V  P  A  T  A  *                           106
663    aacgacgagacatctaacaacagcaactttgcaccctgacggcatgaaatcaatttgaac
723    accgaccctttgaccatgcgacgatcttaaaaaaaaatgcttatatcaatcattatgcac
783    tcctttaactttaagcgaaatgcagtttgtagaattacaccagaccttttaaggtctggt
843    gttatttacaaactattattgtaatcttctgcggtgtttatttacacatatctggtgagg
903    tttgaatgccgagtgcaacacataatttgacgcaaactgatttgaa
```

Fig. S16. ***A. rubens* luqin (LQ)-type precursor (ArLQP).** The nucleotide sequence (lowercase, 948 bases) encoding the precursor protein (uppercase, 149 amino acid residues) is shown. The predicted signal peptide is represented in blue, a putative luqin (LQ)-type peptide is represented in red, a C-terminal glycine (G) residue that is a putative substrate for amidation is represented in orange and putative monobasic and dibasic cleavage sites are represented in green. Two cysteine (C) residues characteristic of the C-terminal region of LQ-type precursors are underlined. The asterisk shows the position of the stop codon. This sequence was determined from contig 1091466 and has been deposited in GenBank under accession number KT601719.

```
1     attaagtgcagatgatgcccaagcctgcgtagacttcccccatcccacacacatcatttg
61    aatacatttactcaaagcactgtatacagtgcgtgttttaaaggccgagtgggtgttgca
121   agtggttgctacgttattgctcgcgtaagatcgactgggccgtgtaaccattgacgcgga
181   taagtaccagacaacatctacaacgaaaaggggtttcataaaaataagagcgcggtgcgc
241   agagctcggcattctactactgacagtcaagcgacacggacacgtacttactttcttgcg
301   agttgtagaaaactctggaggtttttttcagtgaaacacaacgtcttcgataaggataa
361   cagtttaatttttcagcgagacatcgagggtggttttgttggtttctatttggttggcga
421   aggattgcgattggaatgatggcgaggtgtggaggggaaactccacgggctcttcgcacg
                                  M  A  R  C  G  G  E  T  P  R  A  L  R  T     14
481   tggtggctggtaggcctattctgtctggctctttcgctcgtctgccaagcggagattgaa
       W  W  L  V  G  L  F  C  L  A  L  S  L  V  C  Q  A  E  I  E    34
541   gccaacgatgtggaactcgttccggaacctgaagagacgaaagcaaatgagatactgctt
       A  N  D  V  E  L  V  P  E  P  E  E  T  K  A  N  E  I  L  L    54
601   gaagaattgagggatgaacttttcaacgaactccttcaggagcttgaggacgaggttgca
       E  E  L  R  D  E  L  F  N  E  L  L  Q  E  L  E  D  E  V  A    74
661   aagggccttaccccgaaggacgcgatctgtttcacaagaggttgacaagtctcaactct
       K  G  L  T  P  E  G  R  D  L  F  H  K  R  L  T  S  L  N  S    94
721   gagtggcgtgccaagagagggcgcaccaacatgtacgggtcgtctcaactatcaagacta
       E  W  R  A  K  R  G  R  T  N  M  Y  G  S  S  Q  L  S  R  L   114
781   agcagtggattcaacaagcggactctgactgacgatgagagcgcgcttgaagatttattg
       S  S  G  F  N  K  R  T  L  T  D  D  E  S  A  L  E  D  L  L   134
841   gatgacgcagaggtcaaacgcggacgttcttcatttgcaggctctagccgactaactaat
       D  D  A  E  V  K  R  G  R  S  S  F  A  G  S  S  R  L  T  N   154
901   ctgggaagtggcttcaccaagaagagtgatcctggagtatggttggacagtgaggacaaa
       L  G  S  G  F  T  K  K  S  D  P  G  V  W  L  D  S  E  D  K   174
961   agagggcgctcttcattcacgggttcgagccgacttactaatctagcaagtggttttaac
       R  G  R  S  S  F  T  G  S  S  R  L  T  N  L  A  S  G  F  N   194
1021  aagagagatgaagacgcatacctactggatgacttcttaagtaaaaggggtcgctcggca
       K  R  D  E  D  A  Y  L  L  D  D  F  L  S  K  R  G  R  S  A   214
1081  ttcagcggctcaagaggtttgaccaatttagcaagtggcttcaataagagagaagaagct
       F  S  G  S  R  G  L  T  N  L  A  S  G  F  N  K  R  E  E  A   234
1141  gtcaaaaggggcgctcttcctttgccggtgtgagcgggctgacgcaccttggaagtggc
       V  K  R  G  R  S  S  F  A  G  V  S  G  L  T  H  L  G  S  G   254
1201  ttcaataagagaggcgatttcttagaagatgtgtatgcaaatgaagataagagagggcgc
       F  N  K  R  G  D  F  L  E  D  V  Y  A  N  E  D  K  R  G  R   274
1261  tccgctttttcagggtcgaaggggctgacaaacctggccagtggcttcaataagagaagc
       S  A  F  S  G  S  K  G  L  T  N  L  A  S  G  F  N  K  R  S   294
1321  gatggagaccagagcctatgggaggagaacgatgtcaagagagggcgcacttcgctgtca
       D  G  D  Q  S  L  W  E  E  N  D  V  K  R  G  R  T  S  L  S   314
1381  gggtcgagtgggttgacccatttaagtagcgggtt
       G  S  S  G  L  T  H  L  S  S  G                              325
```

Fig. S17. ***A. rubens*** **pedal peptide (PP)/orcokinin-type precursor (ArPPLNP).** The nucleotide sequence (lowercase, 1415 bases) encoding a partial precursor protein sequence (uppercase, 325 amino acid residues) is shown. The predicted signal peptide is represented in blue, seven putative pedal-peptide (PP)-type peptides are represented in red and putative dibasic cleavage sites are represented in green. This sequence was determined from contig 1107802 and has been deposited in GenBank under accession number KT601720.

```
1                                                                 g
2    agacccgtcttgctgttgggttacacgatgcagtggtgcaaggttgtcttgacgttgatg
                                 M  Q  W  C  K  V  V  L  T  L  M    11
62   tcgctctctgtttgtctatgttctcttttgccacggcccagcaaagaccctattgggag
      S  L  S  V  C  L  C  S  L  F  A  T  A  Q  Q  R  P  Y  W  E    31
122  aggagtggatgccatctagtcggttactctaccttggttcagataccaggctgccataag
      R  S  G  C  H  L  V  G  Y  S  T  L  V  Q  I  P  G  C  H  K    51
182  actagggtggatatgaatgcatgccgaggctactgcgtgacctacacactgctgtcaaca
      T  R  V  D  M  N  A  C  R  G  Y  C  V  T  Y  T  L  L  S  T    71
242  ttcaaccagatcgttagtaacaacatacgctattcatcgcgtggtacctgctgcgctata
      F  N  Q  I  V  S  N  N  I  R  Y  S  S  R  G  T  C  C  A  I    91
302  ggagatacacacgatgtaatcgtgatcctcgcatgtgaaaacaacgaacagaagagtgta
      G  D  T  H  D  V  I  V  I  L  A  C  E  N  N  E  Q  K  S  V   111
362  acgtataagtccgctgcctcctgctcatgcacactgtgcacccaggaagatgcctcgcaa
      T  Y  K  S  A  A  S  C  S  C  T  L  C  T  Q  E  D  A  S  Q   131
422  ttgaataatgtatagcaccaacgaaacgccctctattgggatcaacaatatagtttgaat
      L  N  N  V  *                                                 135
482  catccttgagggcgctgttatctcactatgagtcgtgtataaagtaaactagaagcttaa
542  ggactccttctgagttgaagtgacgtgtataggctcagaaatgcgaacattgacgtcgtg
602  taaagccccttcatacgagagaaatttatttagcaagggtccctcgctaaagtgaagcat
662 ggttgtacaa
```

Fig. S18. ***A. rubens*** **glycoprotein hormone α-2 (GPA2)-type precursor 1 (ArGPA2-1).** The nucleotide sequence (lowercase, 671 bases) encoding the precursor protein (uppercase, 135 amino acid residues) is shown. The predicted signal peptide is represented in blue and the putative glycoprotein hormone α-2-type (GPA2)-type subunit (with cysteine (C) residues underlined) is represented in red. The asterisk shows the position of the stop codon. This sequence was determined from contig 1069119 and has been deposited in GenBank under accession number KT601721.

```
1                                                                  aa
3    gagtgtgagggttaaaaaaaaaaccaaaccgattgaatccacccttttgccgttttaact
63   tttggtgtggttatgacaatgaaagtgagcgtaacttttatatatgtcgcctgcacggct
                  M  T  M  K  V  S  V  T  F  I  Y  V  A  C  T  A     16
123  gcactgctgatccttgtgagctccccggtgaagggagcctgggaaccgacggctgggtgc
      A  L  L  I  L  V  S  S  P  V  K  G  A  W  E  P  T  A  G  C     36
183  cacttagtgggttacagaaaagaagtcagggtaccagggtgccacatagaatatgtgaag
      H  L  V  G  Y  R  K  E  V  R  V  P  G  C  H  I  E  Y  V  K     56
243  atgaacgcctgcagaggttactgtatgacgtattccttcctatccgatacagcaacactt
      M  N  A  C  R  G  Y  C  M  T  Y  S  F  L  S  D  T  A  T  L     76
303  gagagaagtggaggcacccaactatttacgtcacacggatcatgctgctccataacatcg
      E  R  S  G  G  T  Q  L  F  T  S  H  G  S  C  C  S  I  T  S     96
363  acacatgacgttcacatcacactacaatgcgaaaacaaccaagtatacaaagacaccttc
      T  H  D  V  H  I  T  L  Q  C  E  N  N  Q  V  Y  K  D  T  F    116
423  aagtctgcaaagacctgtagttgcgccctctgtagtacgcaataaaaaaccgccaaagtt
      K  S  A  K  T  C  S  C  A  L  C  S  T  Q  *                  130
483  acccctgaggtggatttcacaa
```

Fig. S19. ***A. rubens* glycoprotein hormone α-2 (GPA2)-type precursor 2 (ArGPA2-2).** The nucleotide sequence (lowercase, 504 bases) encoding the precursor protein (uppercase, 130 amino acid residues) is shown. The predicted signal peptide is represented in blue and the putative glycoprotein hormone α-2-type (GPA2)-type subunit (with cysteine (C) residues underlined) is represented in red. The asterisk shows the position of the stop codon. This sequence was determined from contig 1041026 and has been deposited in GenBank under accession number KT601722.

```
1                                                                c
2    tctaaaggatctaagccaggaaagatccacatggaccaaggagcagcatacacatccata
                                      M  D  Q  G  A  A  Y  T  S  I   10
62   gtcatggtcacgttggtgatgatgtgggcatgcgcactggccatcaaccccgtcaccaca
      V  M  V  T  L  V  M  M  W  A  C  A  L  A  I  N  P  V  T  T   30
122  acgaattgttacgtgcacaccgctatgaagcatctagtggaaaagcctggttgccggccg
      T  N  C  Y  V  H  T  A  M  K  H  L  V  E  K  P  G  C  R  P   50
182  cacgaactggtggtgtttggatgttggggacgatgtgataccaacgaggttccttcgtta
      H  E  L  V  V  F  G  C  W  G  R  C  D  T  N  E  V  P  S  L   70
242  gatcccccattcgtagaggcctaccatccagtgtgtactctcaccaactacgaggatgta
      D  P  P  F  V  E  A  Y  H  P  V  C  T  L  T  N  Y  E  D  V   90
302  aaagtcaagttaccagactgcgaccccgaggttgacccgacctacacgtaccaaagcgcc
      K  V  K  L  P  D  C  D  P  E  V  D  P  T  Y  T  Y  Q  S  A  110
362  ttatcctgcggctgcgccaacattgacgattcgagtactaagtatagctacaggccggac
      L  S  C  G  C  A  N  I  D  D  S  S  T  K  Y  S  Y  R  P  D  130
422  tatttgtgtcagagaagtagatagtttaaacatcatagatgaaggtctatagaattggg
      Y  F  V  S  E  K  *                                         136
482  ttacaataagttacctgaaagtcattaaccctcagtaaagggcagacaatgagtagcacc
542  actttgtgcggacaaacaattagaagtcaaagggccaaccccattgttaatttgttctaa
602  aatcctttgttcgaaacacttacgattgttttagaaccagtctaattctgttattgttta
662  acaatccatgacacctgaatctaatggttctttatggtttcttgtcccctttcttacatt
722  ataaaacgaacgtgactctcatacatcacactgtcaatacatgtcgaacaaaaacaataa
782  ataacgaagaacacgaattttggaaaactagttgatgatcatattgttcaaacaaaatta
842  gaaataaatagtaaataataaagaaagtgtgaggttggggttactcattcaagatgcgtc
902  attgcaatttaagtgtatctcgcaaaattccactttattattcatgaagtcgatttaaga
962  accctaccctattataaagttggcgtgacaaaccttggccaatgtaaaacaatcaactga
1022 acaaacactaatgtttaaaagaccgtcatatcaacatcattaatcatcaatgtttgctgg
1082 ataccacattcaaccttctcttaagcaacggtgatacatgttggctattcaagatatgtt
1142 ctcacgcttgtgtcaatttacattttacctctggataagacaatttcagaagttctagtt
1202 ctaaaggaaacacccaatggtacaccaattaacattgagaaatggtttggcaacattgtt
1262 tcgttaccccactaaggctatgctgcccatttaactcaagcccaaattatcatgcaaata
1322 ctctctgactcagtaggttatacaccaagtcagacttgacccggacttgacccgatctgg
1382 gtcccgttgggcctgataccatatggtcagtatgaccaggaatctgtttcaaagttaccc
1442 acaaatgtattggtcaaactgatgcagaatacaggtcaacattttgttttggtgaaaaat
1502 ttccgggtcaaccagacccaagcacgggtcaagtcccctctgacccggaatccaagtcaa
1562 ttctgacaggagttttttttt
```

Fig. S20. ***A. rubens* glycoprotein hormone β-5-type (GPB5)-type precursor 1 (ArGPB5-1).** The nucleotide sequence (lowercase, 1582 bases) encoding the precursor protein (uppercase, 136 amino acid residues) is shown. The predicted signal peptide is represented in blue and the putative glycoprotein hormone β-5-type (GPB5)-type subunit (with cysteine (C) residues underlined) is represented in red. The asterisk shows the position of the stop codon. This sequence was determined from contig 1111214 and has been deposited in GenBank under accession number KT601723.

```
1     cacaacttatagatcaccggccgtgctgctgaaataaatgaatgtatatatcacttttct
61    ccgccatcttaagagtttagtgcgaataacgtttatatatgtagtcaatttggatcgtta
121   aaagacacgtccgtcctgctccaacgagaacttctatttccatcgggtgctaaagcggaa
181   cgacatacacgtcgcacaatgttcttcaagtgtcaagcgaggcacagatggactgccacg
                         M  F  F  K  C  Q  A  R  H  R  W  T  A  T   14
241   ttcctgcctctctttgcattggctctcctcattggcaccatcgttgagggcgctcgttac
       F  L  P  L  F  A  L  A  L  L  I  G  T  I  V  E  G  A  R  Y   34
301   caaaaacagagcatgcccttttggacgtaagttgcagagttcgggagtacacaaagtat
       Q  K  Q  S  M  P  F  L  D  V  S  C  R  V  R  E  Y  T  K  Y   54
361   gaggctaagctgcccggatgtatggacgaagtagtgccggcaagagggtgctatggtcgg
       E  A  K  L  P  G  C  M  D  E  V  V  P  A  R  G  C  Y  G  R   74
421   tgccagtcgttcgaggtaccagttttgctaccaccgcacaaagcgtcaagtcacaagatg
       C  Q  S  F  E  V  P  V  L  L  P  P  H  K  A  S  S  H  K  M   94
481   tgcttggtcgaggaaatagagcttcgtagcgtggagttgtcggattgcctaccgggggtc
       C  L  V  E  E  I  E  L  R  S  V  E  L  S  D  C  L  P  G  V  114
541   aacaggacctttgtctatcagagtgccgtacgatgcagatgcaagaaatgcatcgagtct
       N  R  T  F  V  Y  Q  S  A  V  R  C  R  C  K  K  C  I  E  S  134
601   aatacattctgtgcccgaaattgattatagagaaaacaactttttgctgtatgtatttact
       N  T  F  C  A  R  N  *                                       141
661   gtgtatatatttgttaaaacaaatttcaaactggttttcgctttggttttagagggttta
721   caaaatcaaacgtatttaacaaacattttataggtatagtatatgaatcaattccaaata
781   atgctagatgcatctacccgcgctgttcttgattaca
```

Fig. S21. ***A. rubens* glycoprotein hormone β-5-type (GPB5)-type precursor 2 (ArGPB5-2).** The nucleotide sequence (lowercase, 817 bases) encoding the precursor protein (uppercase, 141 amino acid residues) is shown. The predicted signal peptide is represented in blue and the putative glycoprotein hormone β-5-type (GPB5)-type subunit (with cysteine (C) residues underlined) is represented in red. The asterisk shows the position of the stop codon. This sequence was determined from contig 1083001 and has been deposited in GenBank under accession number KT601724.

```
1                                                              ct
3    caaccgggcaaaagcacttccggggggatactgatgataataagcccacacagggacaggg
63   attatgaaaggcagattcaagccttaataaataacctgcgtttttcatacatttgataag
123  tagtacaaaaaggggcggtcagttgtatagttgctatgaacacacacgtacaagtgcgct
183  cttctggtagtaccaaaactcgtgtgataacacattattgactgcgcgtgagattagact
243  tgtcgacatctcgtccaaagctaaaacacattgtgggcaggtcgaactcaaaagacacac
303  cttctccgcgccagtatgtcatgtgttcaacccacgcgcacgcagcctgcgtactgagta
363  ggccctaaggaggtgcacttctttttaattcctgtattacaagacgataaccacagtaa
423  aacttcgtgcatgatacaacacccgcattgcgcaactgtttaatgaactttacccgtgtg
483  gcggctatttttatttgtgagaagtgcatcgggctcaaacgttatagaaagacaagcga
543  cagaaggtagcaccgtactccattgtcatatgaggcatcgttaagtcttaagatacagaa
603  caatctaaacacatgatggcaacccgaaggttaacagcggtacagaacctagcaatattt
                   M  M  A  T  R  R  L  T  A  V  Q  N  L  A  I  F  16
663  gggtttgtggtgttggtattgagcgctacattgtgcacatctcagggattgtcatgttta
      G  F  V  V  L  V  L  S  A  T  L  C  T  S  Q  G  L  S  C  L   36
723  cctcgtcagtacatcaagtatgatgcggtcaaaccaggatgcagaacgcaacggatcact
      P  R  Q  Y  I  K  Y  D  A  V  K  P  G  C  R  T  Q  R  I  T   56
783  atttatgggtgttttggacgctgtcatacgtctgagataccgaagttgttaccaccctac
      I  Y  G  C  F  G  R  C  H  T  S  E  I  P  K  L  L  P  P  Y   76
843  aaagaatccaaccacgccatgtgttcctacggacaaaccgaaagcagagtgattctcctg
      K  E  S  N  H  A  M  C  S  Y  G  Q  T  E  S  R  V  I  L  L   96
903  gatgactgtgacccaggagtcgaccccactttccaatacgaggatgccctgtcgtgtgct
      D  D  C  D  P  G  V  D  P  T  F  Q  Y  E  D  A  L  S  C  A  116
963  tgtaaaaaatgtgaaccttggaatacattctgtcagggattctaaaaaatcggaaaacca
      C  K  K  C  E  P  W  N  T  F  C  Q  G  F  *                 130
1023 aaatttacacacaaacagggaatagtttaaacgtgacgagacgaggtggcattgtctcaa
1083 ctccaactgcacacacgacgctgttttgacgaccagcaagacatacttataataaaagtc
1143 ggtctgggatgaagttattttcccctctcccccattacattagcttatttcctctacac
1203 aatctgcgcccgtccctccaggaggaaaaatgatccttcgataacgttcattgtggattg
1263 accacggggaggtgttgcctttcagctcgctcaaacctaaaaagacattaggcttatcaa
1323 cttttgcaagggatacggcaagtctgactacttctctgtattcagtttgcaaaactccgt
1383 cgacagctcttacaagagaccttgcattgcatttcttt
```

Fig. S22. ***A. rubens*** **glycoprotein hormone β-5-type (GPB5)-type precursor 3 (ArGPB5-3).** The nucleotide sequence (lowercase, 1420 bases) encoding the precursor protein (uppercase, 130 amino acid residues) is shown. The predicted signal peptide is represented in blue and the putative glycoprotein hormone β-5-type (GPB5)-type subunit (with cysteine (C) residues underlined) is represented in red. The asterisk shows the position of the stop codon. This sequence was determined from contig 1107934 and has been deposited in GenBank under accession number KT601725.

```
1                                                                 ca
3    cggctcttcatcaacgactatcaaatgtgttcacgcctgctgccaagtggtatgttttag
63   ctgacactgtataacgtgaatattgctatcggtggaacgacgtttctcttgttattggcg
123  tgggaggtttttagtgtacgcttttacttttcttagagccggcgtgcggggaaaaaggat
183  ttggaaaagttgacgaccatctcttcttcggtcgggaggacggtggggagattgtgtttt
243  ttgtgtgctgagaaggaggtttgtttttaacttgcttctgctgcaatctggttagagtt
303  ggagatacttcattcaagacgagataaatcatggattctcatcatcgaaacacgggcacc
                                      M  D  S  H  H  R  N  T  G  T    10
363  gcgtccgccacggtggtagtaaagtgcgccctcatcgccatcctcctactaggcgtgatc
      A  S  A  T  V  V  V  K  C  A  L  I  A  I  L  L  L  G  V  I    30
423  agccagtgctcggccgtgtgccgtcggcagccactaatacactccatagtccacgagggg
      S  Q  C  S  A  V  C  R  R  Q  P  L  I  H  S  I  V  H  E  G    50
483  tgccagacgaaacggctgcggacgttcggctgccggggaacatgcaactcctactctcgc
      C  Q  T  K  R  L  R  T  F  G  C  R  G  T  C  N  S  Y  S  R    70
543  gtctctccaacggactacacccaaatggagagatcgtgccagtgctgccaagaatcccaa
      V  S  P  T  D  Y  T  Q  M  E  R  S  C  Q  C  C  Q  E  S  Q    90
603  cacgtggttggttttgtggaactcaactgcccgtccctgagcccgccgactcagatcgtg
      H  V  V  G  F  V  E  L  N  C  P  S  L  S  P  P  T  Q  I  V   110
663  gaattcaggcatgttcgctcatgttcgtgtagaccatgcaactcggtgtcgggggtacca
      E  F  R  H  V  R  S  C  S  C  R  P  C  N  S  V  S  G  V  P   130
723  cgtgtaacgaggctagaggatctggaataatgataatacaaaaaa
      R  V  T  R  L  E  D  L  E  *                                 139
```

Fig. S23. ***A. rubens*** **bursicon α-type precursor (ArBAP).** The nucleotide sequence (lowercase, 767 bases) encoding the precursor protein (uppercase, 139 amino acid residues) is shown. The predicted signal peptide is represented in blue and the putative bursicon-α subunit (with cysteine (C) residues underlined) is represented in red. The asterisk shows the position of the stop codon. This sequence was determined from contig 1079029 and has been deposited in GenBank under accession number KT601726.

```
1                                                                   g
2     gtagctaccgtgtacagaatggcaccaatgcaacaccaccatcacctcgccgccattttc
                            M  A  P  M  Q  H  H  H  H  L  A  A  I  F    14
62    atattcagcgtactgtcaatgtgtcttctgcctgacctggttcaagccgtccgtagaggg
       I  F  S  V  L  S  M  C  L  L  P  D  L  V  Q  A  V  R  R  G    34
122   ccggccggcacgtgcgaggtagggatcagctttattacggtagaggaggagtttgaaagt
       P  A  G  T  C  E  V  G  I  S  F  I  T  V  E  E  E  F  E  S    54
182   agtgacgggggtcagtcatctcgtgtacgggtacgactaccgtgaatcagtgtgagggc
       S  D  G  G  S  V  I  S  C  T  G  T  T  T  V  N  Q  C  E  G    74
242   gagtgcgttaccacctcgaccccaagtgtgactgaaccgactggatactcaaagatatgc
       E  C  V  T  T  S  T  P  S  V  T  E  P  T  G  Y  S  K  I  C    94
302   aaatgctgcagggaacagagcttgagaccgaagcaggtaatgctaagtgattgctatgac
       K  C  C  R  E  Q  S  L  R  P  K  Q  V  M  L  S  D  C  Y  D   114
362   agcgcaggaaacgccatcaccggtcaacaatatccagtctacgttccggagccagcttca
       S  A  G  N  A  I  T  G  Q  Q  Y  P  V  Y  V  P  E  P  A  S   134
422   tgtagttgtcaaaaatgctctcgctaattaacataattagggcccgattaggaatctgta
       C  S  C  Q  K  C  S  R  *                                     142
482   acttttgcccgtgtacgaatttgtgcactagacaaactgtatacattataagctatgtat
542   tttgttttacatgattacaagctggttataacaagtaggttctggaccgagtaaggtttg
602   agacaaaactagaactttcaagtatttcccattgtgagttaagtgggggtttccagcctt
662   agcttcacttgggaaaagactgaccaccctcaccaaaagcaggttatatttttccagatt
722   tttgaacacgagttttgagatgaatatacagtgaaaaaaaaggggg
```

Fig. S24. ***A. rubens* bursicon β-type precursor (ArBBP).** The nucleotide sequence (lowercase, 766 bases) encoding the precursor protein (uppercase, 142 amino acid residues) is shown. The predicted signal peptide is represented in blue and the putative bursicon-β subunit (with cysteine (C) residues underlined) is represented in red. The asterisk shows the position of the stop codon. This sequence was determined from contig 1078917 and has been deposited in GenBank under accession number KT601727.

```
1     tcccgttttgattttgtggctgtttgttatcgcttgctactatcgacaactcagacgctt
61    acatcccttcaaacacggtaaaaatggcaaactaccgtctcatcctcgaggccacctgt
                                         M  A  N  Y  R  L  I  L  E  A  T  C    12
121   ctccttgtcctccttataaacaccgccctctacgccgaggctgctgaaaaatactgcgat
       L  L  V  L  L  I  N  T  A  L  Y  A  E  A  A  E  K  Y  C  D    32
181   gaggatttccacatggccgtctacagaacgtgtacagagcacaagcgcagtgggagatcc
       E  D  F  H  M  A  V  Y  R  T  C  T  E  H  K  R  S  G  R  S    52
241   gccttcagcttgaacgacttttccgaagtaactcaaaacggaccgctggatctccccga
       A  F  S  L  N  D  F  F  R  S  N  S  K  R  T  A  G  S  P  R    72
301   ccagacgacgacttttcctgactatgcagaagagacctgagacttacgttggaatggga
       P  D  D  D  F  F  L  T  M  Q  K  R  P  E  T  Y  V  G  M  G    92
361   tcctactgctgtttggtggggtgcacacgtgaccaactatcacaagtctgctaaaactta
       S  Y  C  C  L  V  G  C  T  R  D  Q  L  S  Q  V  C  *        109
421   aagaaatccccattaccacttttgaaaccccttgacaacggaacacacccaacactcc
481   agttctattaatagctttgtcatgaacaaatttaagcatagcccaaccacatggttggaa
541   cagccccaaaataagtaattttgcaatttctattgtccctgctctatgctctatgataa
601   taagcgacgtataaggatatttagttgactatttcatgggtggcttttttatcagagatgc
661   aatgaagtacttatcgacccttcccaatggcgtcacccttgcccagtgccctcactgtg
721   gccaaaaagtgacctcacgaaggctaagaaggcaattggatggacgaaagctgttcttac
781   gtgtacgtgacctctgttacgttttacatgtatacaaggggtctattgttcatcaacgaa
841   ccctatctcttgttaaaaagagggttaagaaaaagacaactgtcagaaacgggaagtaag
901   gaatttgactctcaaataaataattacgaaaaaaatataattactcgctttaaaatgctt
961   taccgaccaaaatgacctctgcataagatatgataggcctatttaaaaaaaaactaaaaa
1021  agcaacaaatagtttatggcctgtatgcatagagctatgcctgtatgacgttttgaccca
1081  agcacaatccatcctttctcggtcatttagccttcgaaaaaagactctgctatatagggt
1141  atttttgtaaatacttaagttcaactataatttaaaaacatgacgtcgctgaccaccat
1201  ctttggtgtacaatggaaaagtgcacgcgagtacgcgctgcacacaatcagccaataata
1261  gcctttcgcacgtgcacaatatcaaagatggcggtcaaggtctacacagcattttctcat
1321  tgaatgctcccccctatgtgaacagagacgttgtataccaaacagatctgagacctttgtt
1381  tgtaacctgtacgaaaacgctttgctggaatttgaggtcgagtgataagatcgtggagtg
1441  tttttgtgggggcgagtgcaatgacacgccagtgttaaatgattaggctcttccaaacca
1501  cggcttgggcttaggcttccggcttaggcttgggcttagcactttctctcacacgtcttc
1561  aaacagacagacggagcttatcaagcctgagccacaagccacctgggacagggcttcggg
1621  agccgaagcaaaagctgtggttttgaaactatcagggtctatgttgagtgaagatggagg
1681  cacactgtgacagttctccacttttaggcttttggtacaatcttaaaacttccgggtcag
1741  aattgatccacattcagagtccagtgcctggtgtctgatgcctgacccatttctgggtca
1801  tactgacccagaaaatgagtcttgatgacccagaaaagggtcaagtacacactgagttaa
1861  gaccccaattttaaaccatgtttcttggccaacctgacctagaaatgggtcagctccatc
1921  acccggaatccgggtcaatattattctgacccaggagattttagagtgcctcttgttatg
1981  taggctatgcaggtatcattaagtaaaatcatgctgaactatgtcttgactgttattcca
2041  tttttgtttcaatttcacatccctgaccgtgattgtaaggataatccgagctgtctctct
2101  tcaccacactaaaataatgaatgaaatgactgatttacagggagtgagtgtttgggaata
2161  tgtgcaaaggcttgaccaagttttgtcgtacctctcgtatgagtgtgagatgcttctgga
2221  aaagtgtgtccataggaataatttgcctagtcagagaagaaggtggtgaaaagcttccac
2281  caactgctgtggacgttgggtgtggggtagggggcaaattaactccatcgagatacagt
2341  agtatacgggatggatgtatttcgtaaatgtatatacttttcaacatctttgaattatga
2401  cgccatcattataataatgattatttatctataatatttattgatcaatgttaaaccaga
2461  tgtagaatatttcttcattatgcagagtagtaaatagtaggaatataagtttttaagttt
2521  gaggagtaaaaaatctagttatttcaaaattgcattccaaatcatggtttatgttaactt
2581  taaaaacatcttgtgttcaacagaaagtaggctttggctttgaagaaaccaataaaagga
2641  aaataaatgaacttttcatcgtaaatgcatctgttattactatagactgtgtggcaggac
2701  atgttgtgcccagtgatggcgaactgtgtacaaacctcttctattatagagcgttctttc
2761  gaagcaacctcttccccatcgatgtaaatttcccatgaggactgaaatttcgagggaaa
2821  gattttcctgtgatactaggagtgcgttttggcgacaccaaccagaaacatggttgagcc
2881  ttttcgcgcttctcggttgaggcgcggtgacgatg
```

Fig. S25. ***A. rubens*** **relaxin-like gonad-stimulating peptide (RGP) precursor (ArRGPP).** The nucleotide sequence (lowercase, 2915 bases) encoding the precursor protein (uppercase, 109 amino acid residues) is shown. The predicted signal peptide is represented in blue, two putative relaxin-like gonad stimulating peptides (RGP) (with cysteine (C) residues underlined) are represented in red, a putative C-peptide is represented in black and putative dibasic cleavage sites are represented in green. The asterisk shows the position of the stop codon. This sequence was determined from contig 1122961 and has been deposited in GenBank under accession number KT601728.

```
1                                                                g
2    gattcagcaggctatataaagagatcaacttttctcacaacttcttcacatttgttggcg
62   gtttgctactcactgtttacacagggatctctggtgttatactcaaaagtacttaagtcg
122  aaacccggagatcatcaacacacggagaggagcctctaacccgcagtattccaacgagat
182  tcggaaatgacatcgtgcagccaccaaatgctggccttgctgtcagccgtttatatttg
             M  T  S  C  S  H  Q  M  L  A  L  L  S  A  V  Y  I  L    18
242  atcttcttccttgggggtctaccagccgtccacgccagaagtgatcatgcgagtgtcaag
      I  F  F  L  G  G  L  P  A  V  H  A  R  S  D  H  A  S  V  K    38
302  cacttctgtggattggaattctcctacgctgtggtgacggcttgtggggaggctaaacga
      H  F  C  G  L  E  F  S  Y  A  V  V  T  A  C  G  E  A  K  R    58
362  tcaatacgctcggcgccctttttgacatgtttccagttttcaaatctccagaacggata
      S  I  R  S  A  P  F  F  D  M  F  P  V  F  K  S  P  E  R  I    78
422  ccagccgacttcgacgattcaagcatgatccatgtgcgaaagaggcaggattaccaggga
      P  A  D  F  D  D  S  S  M  I  H  V  R  K  R  Q  D  Y  Q  G    98
482  atggctacctattgctgtaccaacggatgcaccattagtcaactaaccaactcaggaatt
      M  A  T  Y  C  C  T  N  G  C  T  I  S  Q  L  T  N  S  G  I   118
542  tgctgagagacccccgccccacccaaccctcaccccc
      C  *                                                          119
```

Fig. S26. ***A. rubens* relaxin-like peptide (RLP) precursor 2 (ArRLPP2).** The nucleotide sequence (lowercase, 578 bases) encoding the precursor protein (uppercase, 119 amino acid residues) is shown. The predicted signal peptide is represented in blue, two putative relaxin-like peptides (RLP) (with cysteine (C) residues underlined) are represented in red, a putative C-peptide is represented in black and putative dibasic cleavage sites are represented in green. The asterisk shows the position of the stop codon. This sequence was determined from contig 1055767 and has been deposited in GenBank under accession number KT601729.

```
1                                                                  g
2    aattttacgcgtatataaaagaaagtgtgtgactcctttctcgatccaatcagtctctgg
62   tggtcattgtgagctcgtgatcttagatgtctcaataggtttcttaaaaaacggcaaggag
122  aaacattcttcatcagatgctgagaactcagtttatttaagacttggacattacggagct
182  agttatagacaaagaagcttcatcagtactactacaagaagcttgtgtatagccaagatc
242  catcttcatcaaggatccatttgacgagtcttgttttgaggaacaaagagagaacacttt
302  caagacgtttttcaaggtgtggacatgttcagaaacacatcgacaatgagggcgcttctc
                                 M  F  R  N  T  S  T  M  R  A  L  L    12
362  ctacttgacgtcattttcgttgccttggttctaccaatcacggcatggccgaagatctgc
      L  L  D  V  I  F  V  A  L  V  L  P  I  T  A  W  P  K  I  C    32
422  ggagagcaactggtggagacagtgtctctagtttgcagtacacgaggtttctacagccat
      G  E  Q  L  V  E  T  V  S  L  V  C  S  T  R  G  F  Y  S  H    52
482  agagattcaaaacgagatgttgaggttttccagaatgaacgagcggctaagagcttcctt
      R  D  S  K  R  D  V  E  V  F  Q  N  E  R  A  A  K  S  F  L    72
542  ggttcccgcatcggctcgaggcagcgacggcggacgggtagaatcgccacggagtgctgc
      G  S  R  I  G  S  R  Q  R  R  R  T  G  R  I  A  T  E  C  C    92
602  gacagaatatgtagcttcgacatcgtggagagttactgtaacccttggcccgtggcgata
      D  R  I  C  S  F  D  I  V  E  S  Y  C  N  P  W  P  V  A  I   112
662  gagtcgcgagacccgcctctatctcccgtcgcaccgggcagggtacgggaagataaatct
      E  S  R  D  P  P  L  S  P  V  A  P  G  R  V  R  E  D  K  S   132
722  gcggacgtggactacatgtacaacccggatgttgtggacgtggaagaagccaactcggtc
      A  D  V  D  Y  M  Y  N  P  D  V  V  D  V  E  E  A  N  S  V   152
782  atacagcgcgaggaagatcttatcgatgacatcgagacgcaagaacaggaaatcgaacaa
      I  Q  R  E  E  D  L  I  D  D  I  E  T  Q  E  Q  E  I  E  Q   172
842  gatgaggaacagaacatgcaaactctacccgaagaagacgctgaagacacagatatacga
      D  E  E  Q  N  M  Q  T  L  P  E  E  D  A  E  D  T  D  I  R   192
902  gaaccagaggacgttgaagaatctttcccagttccagtcccgacgaaaaagaggagaaag
      E  P  E  D  V  E  E  S  F  P  V  P  V  P  T  K  K  R  R  K   212
962  gtcgagggaaggagatcgaaagagagcaagaacaaaggtggaaaatcggaagggaaaaac
      V  E  G  R  R  S  K  E  S  K  N  K  G  G  K  S  E  G  K  N   232
1022 aagaagagaagcgggagcagggaaggggggacggtcttctcgtcggagtcgtggaaaaagc
      K  K  R  S  G  S  R  E  G  G  R  S  S  R  R  S  R  G  K  S   252
1082 tcccgatcgaagaagcagagggatggccgggagagaagcaagagatgggagggggcttgat
      S  R  S  K  K  Q  R  D  G  R  E  R  S  K  R  W  E  G  L  D   272
1142 acatcgcacccagtaaaggaacctactgcaaggagtgttttaggtcgagtcgataccaga
      T  S  H  P  V  K  E  P  T  A  R  S  V  L  G  R  V  D  T  R   292
1202 ccattccgtaactttctgtacaaccgctacactgtagacgagaagcgagacactgaaaga
      P  F  R  N  F  L  Y  N  R  Y  T  V  D  E  K  R  D  T  E  R   312
1262 gaaagctatcgagcggtggcgccccttactgggtacaattcacaccgaggggggatcccaa
      E  S  Y  R  A  V  A  P  L  T  G  Y  N  S  H  R  G  G  S  Q   332
1322 cctgacaaccacccaacactagcggcgctatacaaccttgcagttaaactcgccaaggga
      P  D  N  H  P  T  L  A  A  L  Y  N  L  A  V  K  L  A  K  G   352
1382 cttcaacattgacgaataatagtacaagaggaatttatatgaaagtacagcgccctctaa
      L  Q  H  *                                                   355
1442 cgtggttataattataacctaaaaggggtcatgatagttcaatttgtccagttgtgata
1502 agagtgaacaacaatgttcaaacgaatcagctgataaataatacttgtttgtgtttattc
1562 gtttataattcaattttgtttcaacttaattaaatccactgccgatctttaaaagaatca
1622 ccaacattgatattataactttttgaatctcattaattgacttcatgtatcaccattgga
1682 aacgcaatgcttactgtctaaaacagtttgtttctaaagttatgtgttcaacaaatcttc
1742 aattggcgacattcgccgacggtggcagcagacttatcagacatatctggtttgtttgct
1802 gccacctagcgtt
```

Fig. S27. ***A. rubens*** **insulin-like growth factor (IGF)-type precursor 1 (ArIGFP1).** The nucleotide sequence (lowercase, 1814 bases) encoding the precursor protein (uppercase, 355 amino acid residues) is shown. The predicted signal peptide is represented in blue, putative B-domain and A-domains (with cysteine (C) residues underlined) are represented in red, a putative C-peptide is represented in black, a putative D-domain is represented in olive, a putative E-domain is represented in maroon and dibasic cleavage sites are represented in green. The asterisk shows the position of the stop codon. This sequence was determined from contig 1114876 and has been deposited in GenBank under accession number KT601730.

```
1     agattattcaagatgaaccaatatcagctgatagtactattcgaagtgttggctcacgcc
                M   N   Q   Y   Q   L   I   V   L   F   E   V   L   A   H   A    16
61    tctatgctaaactatgcctctccagtccagctgtgtggaagggagttaacggagacgctt
       S   M   L   N   Y   A   S   P   V   Q   L   C   G   R   E   L   T   E   T   L    36
121   aggtcaatctgtggtgatagaggttactattcacctggacaaagtttttcacgtcgagcg
       R   S   I   C   G   D   R   G   Y   Y   S   P   G   Q   S   F   S   R   R   A    56
181   ccgacacacgacggcattgcgacaagatgttgccagtccctttgcgagtcttcaatcctc
       P   T   H   D   G   I   A   T   R   C   C   Q   S   L   C   E   S   S   I   L    76
241   gagacctactgcaatctaccagcaccgccatcgcaaacacaaccatccacagcagcccca
       E   T   Y   C   N   L   P   A   P   P   S   Q   T   Q   P   S   T   A   A   P    96
301   acaacaacaacaaaaatggcgccattaacagaagaccgacggacgaaagatgtggtcgtc
       T   T   T   T   K   M   A   P   L   T   E   D   R   R   T   K   D   V   V   V   116
361   gactactcagaccagctagccaccgaaggctcccaaatgtcgcgtgtggacggcgtactc
       D   Y   S   D   Q   L   A   T   E   G   S   Q   M   S   R   V   D   G   V   L   136
421   acgcatgacaccgtgacgaacagaagtaaaaccacgacagagtcgaacgagggcagttat
       T   H   D   T   V   T   N   R   S   K   T   T   T   E   S   N   E   G   S   Y   156
481   gacaacgaagagggcgcaccttatgataaaccagacgatagcagtccatcagaacgagga
       D   N   E   E   G   A   P   Y   D   K   P   D   D   S   S   P   S   E   R   G   176
541   gaaagcatacaggatgaagataacgaagtaaataaaccggaaccaaacaacattagggac
       E   S   I   Q   D   E   D   N   E   V   N   K   P   E   P   N   N   I   R   D   196
601   aattcaaaagagcgaggtagaaataggacccacaaggggggttagctcggagagaagagca
       N   S   K   E   R   G   R   N   R   T   H   K   G   V   S   S   E   R   R   A   216
661   aataacagcaggaggaggggtctaagcagtgaaaggagaggatcaagcagcagtagaagg
       N   N   S   R   R   R   G   L   S   S   E   R   R   G   S   S   S   S   R   R   236
721   gaagagaagcttagaaggagacgacagagacatcgggaaagggagctgagagaacaacgg
       E   E   K   L   R   R   R   R   Q   R   H   R   E   R   E   L   R   E   Q   R   256
781   aagcagtccaactccaagaggaagtcgaagggagataagaaagaccattctgttgcagcc
       K   Q   S   N   S   K   R   K   S   K   G   D   K   K   D   H   S   V   A   A   276
841   actactccactcgcagtacaagagcgccctctaaagaatggtggtcggaacagcacatcc
       T   T   P   L   A   V   Q   E   R   P   L   K   N   G   G   R   N   S   T   S   296
901   ggggaacattcgtcagtaaacggcaccgaaacagacacggcgggggcggggtcaccagag
       G   E   H   S   S   V   N   G   T   E   T   D   T   A   G   A   G   S   P   E   316
961   gtcaagaaagatgatctgatcaccacgattacggccgtgctcagtgatatgataggcttc
       V   K   K   D   D   L   I   T   T   I   T   A   V   L   S   D   M   I   G   F   336
1021  caaccagacaatggtaatcgctgatcaccccatcaacgagtcgagaactagacgacttta
       Q   P   D   N   G   N   R   *                                                343
1081  acccaatgatttaaagttcagtttgttagtattaactttcactgctcatcactgatcgat
1141  ttcaaactgacttttataaaaactgggagcttggatttggaaagcaacggaaatatttat
1201  tgatatttattatcccagaccccttggactatggataggtaaagacaatgagagatagag
1261  agaggggaaggtgtaattagacatgatgagaaagctatatagcgatgtgacgatttgtgt
1321  ctactttgcactcgggagttgagttttgtcggacgaggagggcggtatgcttaactgtga
1381  accaaatgtacaatttacgaaataatttaaatggttgttaactcttagggcggatgaagc
1441  gctctttcattgatcagatggaagttctcttcattttatacactttagaaggaaattttc
1501  ttcccaagatcaaaaggacgtgttcttcccacgatcatgctcatagcgtccaaaaaactt
1561  acagatacgaaatagcgctcttcctacaaggaattgctcacagtaaagtttataagggca
1621  cccagtctgaaaaacctttttttccacaggcagctgacg
```

Fig. S28. ***A. rubens*** **insulin-like growth factor (IGF)-type precursor 2 (ArIGFP2).** The nucleotide sequence (lowercase, 1659 bases) encoding the precursor protein (uppercase, 343 amino acid residues) is shown. The predicted signal peptide is represented in blue, putative B-domain and A-domains (with cysteine residues underlined) are represented in red, a putative C-peptide is represented in black, a putative E-domain is represented in maroon and dibasic cleavage sites are represented in green The asterisk shows the position of the stop codon. This sequence was determined from contig 1112524 and has been deposited in GenBank under accession number KT601731.

```
1    ctcgagagtagctacttgacacatacacactgcgtaacactcgggtcgcaagctacttct
61   ttgctcgctggccgtagacaactctttgatatctctcgaccaccatcaaaaacgcagaca
121  caacacaatttcgcgtggtttatttaaacgcacacgaggggcctacctgccttgggtttt
181  ttccgggtgtttaattttgtgtactcaccgtcccaaatgaagggccaacaccttctagcc
                                                M  K  G  Q  H  L  L  A    8
241  gtagcagtggttgtcgtcgccggttcgtttggaatcatcgaggcgtactctccatttggg
      V  A  V  V  V  V  A  G  S  F  G  I  I  E  A  Y  S  P  F  G   28
301  ggctataaccgagcaccttttgacaatgtttgggtgcgagcggacagcatggctcgtgga
      G  Y  N  R  A  P  F  D  N  V  W  V  R  A  D  S  M  A  R  G   48
361  ggctcgacggggaggacgaagccaacgaacagcgaatgacgggagccaagcgaccggct
      G  S  T  G  E  D  E  A  N  E  Q  R  M  T  G  A  K  R  P  A   68
421  ggggcctcagcgttccactccgctctgtcgtacggcaaacgaggcgacgatgacagcgcc
      G  A  S  A  F  H  S  A  L  S  Y  G  K  R  G  D  D  D  S  A   88
481  gaagtggagcgccgagcctaccactcggccctgcccttcggcaagagaacacccatcgag
      E  V  E  R  R  A  Y  H  S  A  L  P  F  G  K  R  T  P  I  E  108
541  aaacgcgcctaccacacaggtctccccttcggaaagagggacgacgaagccgccgaacaa
      K  R  A  Y  H  T  G  L  P  F  G  K  R  D  D  E  A  A  E  Q  128
601  gacgccatgatggagaggcgtggcttcaactcggccctgatgttcggtaaacgactacac
      D  A  M  M  E  R  R  G  F  N  S  A  L  M  F  G  K  R  L  H  148
661  agtgctctaccgttcggtaagcgcggctaccacagtgctctgccgttcgggaagagattg
      S  A  L  P  F  G  K  R  G  Y  H  S  A  L  P  F  G  K  R  L  168
721  gataccactgatgaaggagatatcatcgagagaagaggttaccatagcgggctaccgttc
      D  T  T  D  E  G  D  I  I  E  R  R  G  Y  H  S  G  L  P  F  188
781  ggcaagcgcgctactgacgatgaagccgttaatgacatactagaccaattaagaagcgaa
      G  K  R  A  T  D  D  E  A  V  N  D  I  L  D  Q  L  R  S  E  208
841  gagaattgacttttataaagaactgtaatccttaaaaatcagcttatatactaatctaac
      E  N  *                                                      210
901  agtgcttaggatacgacaatatcggttggtgatcagtttagttaccttacaaaccctct
961  ctcaactagtcatatcaaccttagcgaggacaacgcgggttacacaatgtttcgcatact
1021 taaaaaaa
```

Fig. S29. ***A. rubens* L-type SALMFamide precursor.** The nucleotide sequence (lowercase, 1028 bases) encoding the precursor protein (uppercase, 210 amino acid residues) is shown. The predicted signal peptide is represented in blue, seven putative L-type SALMFamide peptides are represented in red, C-terminal glycine (G) residues that are putative substrates for amidation are represented in orange and putative dibasic cleavage sites are represented in green. The asterisk shows the position of the stop codon. This sequence was determined from contigs 1073975 [1-46], 192719 [47-61], 104468 [62-74] and 1057915 [75-210] and has been deposited in GenBank under accession number KT601732.

```
1     agataacgacgggaaaaagtcaagataatttcacgtacacaactcttcagaagaagaacg
61    gaagcaagaaaggataagatgatggtgcgattcgtagccttactcggggcagtcagccta
                         M  M  V  R  F  V  A  L  L  G  A  V  S  L   14
121   ctggtatgtcaatctgcaggacttgatgccgcagacgtcgaggaacaagacgagttcaac
       L  V  C  Q  S  A  G  L  D  A  A  D  V  E  E  Q  D  E  F  N   34
181   aaaccctatgctcctgacagttcgtatgcggatttaaatgcactttgggcaacaatgtg
       K  P  Y  A  P  D  S  S  Y  A  D  L  N  A  L  L  G  N  N  V   54
241   ccaagtctacacagcgcctccaagcgtcaacaaagtgacagggagcgtgaggttgaagca
       P  S  L  H  S  A  S  K  R  Q  Q  S  D  R  E  R  E  V  E  A   74
301   gcccagacgcaatttacccgtatggaagaagaactgatcccaggaaagcgtctggtgga
       A  Q  T  Q  F  Y  P  Y  G  R  R  T  D  P  R  K  A  S  G  G   94
361   ttcacctttggcaagagagggcagtattttatccccattccgtacgagaaacgagagatg
       F  T  F  G  K  R  G  Q  Y  F  I  P  I  P  Y  E  K  R  E  M  114
421   gatgaggtgaacccgtacagcgtagctaagcgcgacgacgagctgaccggactagaggag
       D  E  V  N  P  Y  S  V  A  K  R  D  D  E  L  T  G  L  E  E  134
481   taccaagctagcaagaggtcaggtccttattcctttaacagcgggctgacctttggcaag
       Y  Q  A  S  K  R  S  G  P  Y  S  F  N  S  G  L  T  F  G  K  154
541   agggaacccgagaagaggaacatattcggatcttatgacttcgggaagcgggcttacggc
       R  E  P  E  K  R  N  I  F  G  S  Y  D  F  G  K  R  A  Y  G  174
601   aacaatttcagcttcggcaagcgaggcatgggagtgtccagttttagctttggcaaacga
       N  N  F  S  F  G  K  R  G  M  G  V  S  S  F  S  F  G  K  R  194
661   tccggacttgagggtgaacaaatgatgccggaagacaaacgggcgttcggagacttttcc
       S  G  L  E  G  E  Q  M  M  P  E  D  K  R  A  F  G  D  F  S  214
721   ttcggcaagcgcaataatggtctgtccagcttcacattcggcaagcgagagggtgaacga
       F  G  K  R  N  N  G  L  S  S  F  T  F  G  K  R  E  G  E  R  234
781   tagaacacgagagggcgccatactgtctacaatgtgataattatagtatcttaattattt
       *
841   caaaaccatacttgataagaaataactgcttgcgttttgagttaaaacatcagttccaag
901   tatacaaacaaattttaaataacattgtttaaagcacggattttgaattaaaatgaaacg
961   ataacgcaaaagtgtgtaaatataagcatagtaagctatcaacatctagaattatggaag
1021  atatttcaagatactaaaacttggaagctttagttaattttagatctggttgagattcaa
1081  gtcttcttttctagattttgtttccagccagggcttttactatccttaacgtttggggaa
1141  attgccagggttgtggtcaccttgttaaggtcctaaacaacacatttttttgttagtaaaa
1201  aattacccaattaagacaattcatgagtaagcgacaaaaacttaataatttagcatattt
1261  gcttacgttagttactacaacaataattgaaactttcttaaacacggtttttctagtatg
1321  ctttcctggtcagcaattaacttgtagatgtttttcaaatactatgaattcatatctacg
1381  tagaaatcttaaagatgttttctgtgattaagtttagaacaaccttagccaacaatctcc
```

Fig. S30. ***A. rubens*** **F-type SALMFamide precursor.** The nucleotide sequence (lowercase, 1440 bases) encoding the precursor protein (uppercase, 233 amino acid residues) is shown. The predicted signal peptide is represented in blue, seven putative F-type SALMFamide peptides and one putative L-type SALMFamide peptide are represented in red, C-terminal glycine (G) residues that are putative substrates for amidation are represented in orange and putative dibasic cleavage sites are represented in green. The asterisk shows the position of the stop codon. This sequence was determined from contig 1108394 and has been deposited in GenBank under accession number KP330476.

```
1    acacttccatctcggcatcaacccggagcagacgaaaggaattgtacatcatctttttgc
61   tgcgttacttttcccgccagtattcacgaccctgactgaaacattggactacttgttgtt
121  aaagcaaacgacagacggggagacctactctcgtggaacttcgatttatctatctgataa
181  atcttgcaaccagagcttgaaatggcaagattggacttgattctacttcttctatcggtg
                             M  A  R  L  D  L  I  L  L  L  L  S  V    13
241  gtagtggctgcagtggtgctccagtgccgggctgacgatgaaaacaccgatgggcaaggc
      V  V  A  A  V  V  L  Q  C  R  A  D  D  E  N  T  D  G  Q  G    33
301  gccgccgatgtagacgaagttctaaaacaactatatgcagaagatgagaatgatgacgat
      A  A  D  V  D  E  V  L  K  Q  L  Y  A  E  D  E  N  D  D  D    53
361  aaacgcgccaaccactacgctagtggacgccaacgtacaagggggaaggccggctaccgt
      K  R  A  N  H  Y  A  S  G  R  Q  R  T  R  G  K  A  G  Y  R    73
421  cgggtgactaaatccgacatggacgcagtagccggactagaagacatggatgaggagaaa
      R  V  T  K  S  D  M  D  A  V  A  G  L  E  D  M  D  E  E  K    93
481  cgggctaactaccgcgcctcagtcagtcgtggtggcaagaagggattccgtagatacacc
      R  A  N  Y  R  A  S  V  S  R  G  G  K  K  G  F  R  R  Y  T   113
541  aaatccgatggagacgaagaggagatccccgaactggaagagatggatgaggaaaagaga
      K  S  D  G  D  E  E  E  I  P  E  L  E  E  M  D  E  E  K  R   133
601  gccaactacagagccactatgaacggcggacgtaaaccccaccgcttcaaccgattcacc
      A  N  Y  R  A  T  M  N  G  G  R  K  P  H  R  F  N  R  F  T   153
661  aaggccgacggagacggagaggaggaggaaggaccggaggatctggagggggaggatata
      K  A  D  G  D  G  E  E  E  E  G  P  E  D  L  E  G  E  D  I   173
721  gacgaagataaacgagccaactaccacgcccgaggtggcaagcctagaggtggattccgc
      D  E  D  K  R  A  N  Y  H  A  R  G  G  K  P  R  G  G  F  R   193
781  aggtacacgaagtcggacgaggatcaagatgctatggagcaggcccctgccgaggaaatg
      R  Y  T  K  S  D  E  D  Q  D  A  M  E  Q  A  P  A  E  E  M   213
841  gacgaagataagagagccaactaccacgcacgaggcggcaagcctaggggtgggttccgc
      D  E  D  K  R  A  N  Y  H  A  R  G  G  K  P  R  G  G  F  R   233
901  aggtacacaaagtcagacgaagatctcgccatgggggaagcagcagccgatgaactggac
      R  Y  T  K  S  D  E  D  L  A  M  G  E  A  A  A  D  E  L  D   253
961  gaggaaaagcgcgccaattaccacgcttcacgcgggagacagaggggcaacgggcgtcga
      E  E  K  R  A  N  Y  H  A  S  R  G  R  Q  R  G  N  G  R  R   273
1021 aattagattgtgacaaaaaagtaaaatatcagttacacaacagtttaaactctttttcat
      N  *                                                          274
1081 tatctggtctggcggaaatcatgcagaggggggaagggggggacacacatcaaaacaaaatc
1141 taagcatcttaactttcagtaagttttcattttgacctagggatttattgccgcaatgtc
1201 tttgacttaaattatcgctttagtgtattcaatcacgaaagctcgtttaaaccactcccc
1261 aatttttagtggaaaacattcactcatggccagagaaattaatattgtcagatattatgt
1321 ctttatctaaagacgactgacaaggtggcagcaacatggctaaaacaaattatgttttca
1381 aaaaattggctttacacagacacccattgagctttgaagcaagtgttggcgaaaacagaa
1441 acaaaacaaaccaataaaagatgaaaagttaaagtgaccttggacaaagacaagattttt
1501 cggcaattataccagctcgacgtgatttgtggtatagagtatctgcgccacaatattgat
1561 ggaggctcttgtgattagattgtatttttcttaagttcataaacaaatgtgcattatgta
1621 taacttcacatgcatttggttaccgattactctgtaacattaatcattgtattttataag
1681 tgtaaatatcctcatcattctatatttgtaagtgttgctggtgttgctcttcaaaaacaa
1741 aacacaaaggcgaagaatgaaagaaaaagagtaacaaaacgcccataaactgagattcaa
1801 attggttgcttggtgcattctggtgcatttagctgcaggtataaaagaaaaactgagtg
1861 tacataaaagcagaaaatagatgattgcccggagttggtttttctttaagtggaaaacc
1921 ttctatttctttcgagggaaattttggacacttgatctggaggaaggctagattcagaag
1981 ttctaacaaacaacaaagacctttcaagcttttaacacacaaactataaaagcgcgcaaa
2041 aacaagctcggcataggcttacttattattatgaagtgcacttcaattagcaaccatgtc
2101 accatacatacttattcaaatttatacatgcttattcaaaatttaatcttggaaaaatct
2161 ggtaaaaataaatgtattagtttgccaatgcttaaaggaagaaagggtactgtgcgacaa
2221 ctttgactggtaactaatggttaattagtggttgaattcgaaaagtctatggtttcattg
2281 tgtggtaactaatttaatcaactgatcaaccatgttgacaaagaaggcgattgtcaaac
2341 tataatcaaccggagttaccagtccagcttgctcgtaatgctatttttttcgtccgttagt
2401 gtaaaccacattctctcgatttaacagtagtttaaacatgaaacgcacctgagaattaag
2461 tgatgagtagccgtaacagctgcaaattatgtggcttttcttgtcatcgaaagcaaaata
2521 aagaagtagtcaaagttaaaccaaaaaaaaaaa
```

Fig. S31. ***A. rubens* AN peptide-type precursor (ArANPP).** The nucleotide sequence (lowercase, 2553 bases) encoding the precursor protein (uppercase, 274 amino acid residues) is shown. The predicted signal peptide is represented in blue, six putative AN-type peptides are represented in red, C-terminal glycine (G) residues that are putative substrates for amidation are represented in orange and putative dibasic cleavage sites are represented in green. The asterisk shows the position of the stop codon. This sequence was determined from contig 1121231 and has been deposited in GenBank under accession number KT601733.

```
1     cttgggtaggtggacgtcagagagatgaacagcacggtgggttgggtaggcggactcctg
61    gtgagtataaatggcccgaatgtcacagctcaaactggcaatagacacaactcagatacc
121   tgtgagcttgatcagttagagaattctactctggaattgagaagtgcgaaacacagcaac
181   gaattgaacaggtatacacccagcatcctccgtaaactacagttatctgcatcacctgcg
241   aaacacagccaccacatattcacaaagatgaggacagcactggtaacgttttagccatg
                                                M  R  T  A  L  V  T  F  L  A  M    11
301   cttctggttggcgacctgatagtctcagcattacccatcgacaatgaacaagacagtgac
       L  L  V  G  D  L  I  V  S  A  L  P  I  D  N  E  Q  D  S  D    31
361   cccatcttcgatcacctctacacccgaaacatagtcgaacgcaggagccgaaaagacctc
       P  I  F  D  H  L  Y  T  R  N  I  V  E  R  R  S  R  K  D  L    51
421   accaaatgcatctctgagtgtgtatcctgcgctaagtatgccggactctacgctgataaa
       T  K  C  I  S  E  C  V  S  C  A  K  Y  A  G  L  Y  A  D  K    71
481   tgcgtacgaggctgcagtagtaaaacaagcgggaaagggatcatcaataagacggagttc
       C  V  R  G  C  S  S  K  T  S  G  K  G  I  I  N  K  T  E  F    91
541   gacgcgtggagtgcatgtgagcagttcttacaccgttgagtcttaagttactattacgtt
       D  A  W  S  A  C  E  Q  F  L  H  R  *                         103
601   tctttgtttttcgttctctcctttgttatgaactctttcgaccataaacattgaccttttt
661   tgcttttacttgaactggaagacacaattaaaaccccgatcctcatcagttaaagtctt
721   tgagatgtctgaagtaagcagagagtaggtaccaagttataggaatacatgggtttgaga
781   tggtaccgaccccttggttgttcaccca
```

Fig. S32. ***A. rubens*** **Arnp11 precursor.** The nucleotide sequence (lowercase, 808 bases) encoding the precursor protein (uppercase, 103 amino acid residues) is shown. The predicted signal peptide is represented in blue, a putative Arnp11 peptide (with cysteine (C) residues underlined) is represented in red and a putative dibasic cleavage site is represented in green. The asterisk shows the position of the stop codon. This sequence was determined from contig 1082355 and has been deposited in GenBank under accession number KT601734.

```
1                                                                 ca
3    gagctttaggtcagttacaaagtcagtgtggatttcttagtgctgtaaagccatgccgta
63   caatgtataaaggagcaggctcaaagtgtcctaggctaccttgtctaaaactcaggcgat
123  cctatctactgcaacatacagtgataacacaccaacgtagaacaaccaaatgctcatatc
183  atttcactgagactgagattttgcgttgggatcaagtccctacggtgattttgtattgcg
243  tttgctcatctccaaagacagccaaggatttctacgtctcagaaagagttggagttaacg
303  accatcgcaagactcgtctttcctggaagagataacaagccaaataaaattttcaaaatg
                                                               M    1
363  aaatctattttgtgcatatgcgccatcgcgctgctggttgaggtcgtcataggagcacct
      K  S  I  L  C  I  C  A  I  A  L  L  V  E  V  V  I  G  A  P   21
423  tttgcagaaatggccggcgacatgtccgatgaagactcgcaactcttcagtaagagagta
      F  A  E  M  A  G  D  M  S  D  E  D  S  Q  L  F  S  K  R  V   41
483  cgagacatcatctcaccacttagtcacgatctggacaagaggaaggcaaacctttgcgcc
      R  D  I  I  S  P  L  S  H  D  L  D  K  R  K  A  N  L  C  A   61
543  atggactgtttcagctgcttcaaaatgatccgaaacgtctccccagaccagtgtgtaaca
      M  D  C  F  S  C  F  K  M  I  R  N  V  S  P  D  Q  C  V  T   81
603  ggctgccagaagaagtctatcagtgatggatcatacagctacgacaggatgtggaaccgc
      G  C  Q  K  K  S  I  S  D  G  S  Y  S  Y  D  R  M  W  N  R  101
663  tgttcaagttacctcaccggacgacgaagataaagccttgaccgaagactagatgaaagg
      C  S  S  Y  L  T  G  R  R  R  *                              111
723  gccaccagttttgaacgaagactagtaaaaaaaagggccaccagttttaaaatgataacg
783  gaatgtttcaacaaataataattgtaaccgagtatataaccacaaacagcagcaagtaca
843  ttcccatttgcagaagagtgaattgacttccaaagtcccacaggtctatgcgggatttct
903  ttctttccttttccgctcaaaccattgtatacacacaatctataaatggtgtgggttgag
963  taaaacacgagaaggaaaccttcgttaagttatctcaatgtaaatacagctcttgatttg
1023 gggccaaattgccttggagtttcggttggttaaatgtcaacagcttctgacacattttag
1083 cccagtcgttaaactgtaagccaaaaatcaacaatcagcaacaaaaaataaaactatcat
1143 aagcacgtgatcggtcatgtgattattccaggagatttggcacttaaaaacaaaacaagt
1203 gcaatcattttgtgctaacccaagtaagagaatagtattcaatattgcacaaaaaa
```

Fig. S33. ***A. rubens*** **Arnp15a precursor**. The nucleotide sequence (lowercase, 1258 bases) encoding the precursor protein (uppercase, 111 amino acid residues) is shown. The predicted signal peptide is represented in blue, a putative Arnp15 peptide (with cysteine (C) residues underlined) is represented in red, a C-terminal glycine (G) residue that is a putative substrate for amidation is represented in orange and putative dibasic/tribasic cleavage sites are represented in green. The asterisk shows the position of the stop codon. This sequence was determined from contig 1103866 and has been deposited in GenBank under accession number KT601735.

```
1    gacgtttcgatcagtatgcctgttcgtcatcagaacctaccaataaattagtcactttgt
61   gattaaaaagccaggcaggctgatcgtgctattattggattcgccccctctggctccagt
121  actgagacagaaaccctaattgattaagccagttttcacacttcatattaacgactgcgt
181  cacaatgctgtactctcaaaatatatagttccagtgatgtattatacggtgacaaccgga
241  aactgtgggtgtttttaattacttgtgtatgtatcaagtgatatcaatccgccgacatgg
301  tttatgcgcgtgcgcacatatgcaattatgtcgtaagcatgacgtcataaccatgtacat
361  attcaaatcacttcgggtgtaacctttttctttataaagtggtgcaagctttccaagcaac
421  aacactgtcgatattgttcacacacgaaacgccacaacgaagttgcacaacgcaagtagc
481  ccgatccacgtagttcatcaacgcatattgctatcaaatgtaccatcgcagttggtacca
541  cagcctggtcgtgtatacatacaacttgagataacaacagtcgcgtctgacttggatgag
601  agtgtgaaaaaaacacatttggagagtcttaaaactgtatttatttgctgcttcgcacca
661  actgctggcttacaccgaccaaaagacaaaatgtccgcatctacaatcacaacccttctg
                                             M  S  A  S  T  I  T  T  L  L   10
721  ctctcactcgccgcactttcagtctttctgccaaacgcctccgcagaatggtcacccgac
      L  S  L  A  A  L  S  V  F  L  P  N  A  S  A  E  W  S  P  D   30
781  aacgacaacaacaacgaccacaagagggcgcaaatgtcagcccatgattggctgaacagc
      N  D  N  N  N  D  H  K  R  A  Q  M  S  A  H  D  W  L  N  S   50
841  cttttggcgggaaatgacgtcggacacgcaaagaccaaaaggattgccaaagcatgtgca
      L  L  A  G  N  D  V  G  H  A  K  T  K  R  I  A  K  A  C  A   70
901  attgactgtttgaactgcggtatgatgttccaggtccaagcctactcacaaggagattgc
      I  D  C  L  N  C  G  M  M  F  Q  V  Q  A  Y  S  Q  G  D  C   90
961  ctcacagcttgccagaatgatgacaaccacagggaccctagttgccatgaacacatcaca
      L  T  A  C  Q  N  D  D  N  H  R  D  P  S  C  H  E  H  I  T  110
1021 atgtaaaagaaactattggacaatatccatgatggggaacttcttaaaaaaacaacagca
      M  *                                                         111
1081 gggaccaacattatgtcatcgagaaatttgttttattgaggggttgagcactaatagtgg
1141 gtttgctgatacagctatgagcccgtcccgcagtacgttttgttaaaagcactatggaca
1201 ctattggtaattactc
```

Fig. S34. ***A. rubens* Arnp15b precursor.** The nucleotide sequence (lowercase, 1216 bases) encoding the precursor protein (uppercase, 111 amino acid residues) is shown. The predicted signal peptide is represented in blue, a putative Arnp15 peptide (with cysteine (C) residues underlined) is represented in red and a putative dibasic cleavage site is represented in green. The asterisk shows the position of the stop codon. This sequence was determined from contig 1102592 and has been deposited in GenBank under accession number KT601736.

```
1                                                                  a
2    cagtgtcactacaacgggagccccctctctctcttctgtcactggtacgttattgtcac
62   caatcacgcgtgtgacaggatataaaagcaatagcattgtatgggtagtcaaagcatttc
122  aaagtcgccccatcacagtgtcactacaacgggaaccggtgatttttactctacttctt
182  cttctgtttttagtgcatctttcgtcgaagtccgtacctcatcaaagttaatcgcccac
242  atagtgtaaaacaccaccacacacacaatccacacgatgcagtcgtcaacagtagttacc
                                             M  Q  S  S  T  V  V  T     8
302  ttggcaacgtgtctgatcatcgccacaattgtaacagagtcagccagcgcattagccttc
      L  A  T  C  L  I  I  A  T  I  V  T  E  S  A  S  A  L  A  F    28
362  caaggttctcaagacagagcgaaaagattgttctgggtggacaaaaagagtgtgaatgaa
      Q  G  S  Q  D  R  A  K  R  L  F  W  V  D  K  K  S  V  N  E    48
422  gatacaacagtttgcgtcagagcttcttcagcggacgatatagctgagtgtttcatcacg
      D  T  T  V  C  V  R  A  S  S  A  D  D  I  A  E  C  F  I  T    68
482  gagtgcattaaacatcagatcaactgcgagatgatctgcggaagtgacagcgaaccgtgc
      E  C  I  K  H  Q  I  N  C  E  M  I  C  G  S  D  S  E  P  C    88
542  cattctctttgcaaagccaagaagtccgattgcgccatcaactgcctagaaagggaacaa
      H  S  L  C  K  A  K  K  S  D  C  A  I  N  C  L  E  R  E  Q   108
602  caatccatgagccaatgaggacagccgacctatcagcagatgactgcaaagtgaacaggc
      Q  S  M  S  Q  *                                             113
662  tgtacaattctgaccaataaaaatcctcgacccactaaaacctcaatgtcttgacctgac
722  ctttgaccggaaaatgattttccttccattgtttttccgggtgggttggaaacaaatct
782  tcttgtttttttgttgttgaatccagactgataattaatttatcaatccttgaacgaact
842  tgatttctttctttttgaaaatgttgttgtttaatcaaatggtttataaaacaacacat
902  caagaaggagataacacaataattaaatatctacgaataagcaacatagttttacttact
962 ttaaaatactagtttctttgatttaacaacgcgttttaaagggaatgctaactatctgac
1022 cgaatcagaatcaattacaaaatgttcagaagtttatattacagacttgaatataaaaaa
1082 atatgtattttttttatccacgtaaaatcctcgtggatttatgtttgaagaagactcgtc
1142 taattttaaaacaatattgatataaacatttattgtaaatataattgttgaataatgcac
1202 aggaaaagtcaacattgtgaaagtaaggcagttttaagtgatcgttatgcaaataagac
1262 aggagtagtacacaccggaattaaacaacttggttccgagattcacattttagaagcttg
1322 tgttgaaaatagcgccgccaacagacagagagagtagaaacaaaaaa
```

Fig. S35. ***A. rubens* Arnp18 precursor.** The nucleotide sequence (lowercase, 1368 bases) encoding the precursor protein (uppercase, 113 amino acid residues) is shown. The predicted signal peptide is represented in blue, a putative Arnp18 peptide (with cysteine (C) residues underlined) is represented in red and a putative dibasic cleavage site is represented in green. The asterisk shows the position of the stop codon. This sequence was determined from contig 1106730 and has been deposited in GenBank under accession number KT601737.

```
1    atgcagagaaaaaaatgaaaaaacaaacacacaaacagagaaaaaatgaataaaatgggt
61   gcagtaaacaaatgggtttattggagaagaggtctgaatgggctcactgtgtccgcaagt
121  tcatctctctagttctctcccttccactctgggtcattctgctcagtcactgcactaaat
181  ccctgtaagttattggattcaggtgtatccacaagatcgctgagtttgacattgtccagt
241  ctctggatgatggcttcaagttcgtcttcatcttcctcaaagtggcatcccgtgtaggca
301  ttattctccatacagtaacgtaggaaattgttccagttgttgtccttgcatagcatgtat
361  gggttacccactaaaccaacaacgccttcgctcgtgtgactgcaacattcaatcttttg
421  gggttcttgagaaagccgagttggtagtcaatgtccatctggatgtgttctgctttagtg
481  ctgcggactgtggacacgatgatgaccaacctctcctgaccttgaaactcttcaacggac
541  cccaccttgatgttcttatggtgacgcttctgtaggactttctgtagcttttgaacctgt
601  cgacgatatggtgaaatgatcccaatgtcagactccttaatttgtctccccccctcgctta
661  ttcagcaaatcctccacatattccacaacgatcgtcacctcggctaggttgaagaaggac
721  ggactatgtccctcacgctcatccttcccttctaccccatgaaacaaaatgggaaagttc
781  ttctgaggtagcctgtcccaactgcacagcgacgtcctcacagcttcatctgcatgaacc
841  tccagctcatttgcatagaacatctgattgggcagtttgaggatagcagggtgagagcga
901  tagttacgcagtagcttggtcaggactctgctgtcgtaatgcggctcagcctcctgacgt
961  tgatagatatcgcattcagtcatcagtcgctcaagtaaagataaacctagtccatgttca
1021 atagcaagaggtgacctcagaatgggaccaagttgcttggggtcaccggccagaaccact
1081 tgacctcctttggtgttctttggattcaggagattagacagggctataatggcttccggt
1141 tctactgcatgaccagcctcatcaatgaatacatgagtgaaatgattcgagggaaagtca
1201 gctgtagacagtctgcctgcagtacacagtgtggtcaccaggattctttttccatcagc
1261 tcttctttagaagggtagaaggtttccctggttccccgcttatcaaagttacacacatcc
1321 ttctccttcaggatttcatcaacagtcttccaatctcgactctgtgcgttgattctcatg
1381 aggctagtcttggcaatgggcgtgcccccggtcagaaggcgtgtcgcgatgaggtcagct
1441 gcactattagatggcgcgcacgcaaggatataacttccacgaagttgatggtatgtctgc
1501 ttgatggcctctacaatggtgactgtcttgcctgtgcctggtgggccaaagactaggtaa
1561 ggactagggcgagatgacccacaaacaatgtgcttaacggctttgatctgttccacgttg
                                                M  C  L  T  A  L  I  C  S  T  L    11
1621 gtggcgagtttcttgtcaaagaacagtctggcaaaatcactgtcatcacctggcacggtg
      V  A  S  F  L  S  K  N  S  L  A  K  S  L  S  S  P  G  T  V    31
1681 cagagtttctttgtcgttccgatgttggatcggtcgggaaagaggagtttacccagatca
      Q  S  F  F  V  V  P  M  L  D  R  S  G  K  R  S  L  P  R  S    51
1741 ttcttgagggcgtcctctatagcattgtgctgtagtcgcaacggcaaacggttgaacgta
      F  L  R  A  S  S  I  A  L  C  C  S  R  N  G  K  R  L  N  V    71
1801 aactcaatgtcaaacttgagtccttgggtaaacctattcaccaaatctttggagaatccc
      N  S  M  S  N  L  S  P  W  V  N  L  F  T  K  S  L  E  N  P    91
1861 aagaccacttcttccatttccacccggtggacataacccttgtaggtcttgccatcttca
      K  T  T  S  S  I  S  T  R  W  T  *                         102
1921 gagtcagtactgaagcgggcaaacagatgatcacctttgaggaccgagggacggttctca
1981 gcaagacctggaattctaagtcgtaggaatttgccgtcctgctgcatgggcactcccttc
2041 atatcgtacctcttgatgtcaacttccatctggcactcctctacgtagagaaggttggat
2101 aacttactgacgtagttcacccaagccagatcaggcttaatggtttttctgacttcctct
2161 attttgtgtttattgttcagcatccagcggagatggtctggaattgggtaatacttggct
2221 tgtctcatcttcagtttgctcttggaggactgtggtaggggcactccttcaacagtctct
2281 cctttccgttgccttcttgccacccttggtgggcgttggtaaggaacagacgggggtaga
2341 tccttcgctacctcgctctccacctccacagtcacaaagcgcagaatatgaaatacctcg
2401 ttggatggtactttcttgaaggcaagtatgagtgggctgctgagaacacccacggagtct
2461 cccactacagccacctgaatacagtactcactccctggtttgaggtggaccactttctta
2521 gactgggtcacatcatactcatccgtcagcacaaaggcaggctggactttcaacagcaca
2581 caagataggaatgcggtagttaagtcatcacttacattcttgatggtaatcctgaaggtt
2641 tgactggcactccgcgccagtttcattgcaatggtcccatcctgaccatcatgctcactg
2701 gcaacctctacgccattcttattttcaatgaactttgacctgctcttcttgacacatttg
2761 aagatcatttgctgtcgatgtctactcccttggagatgtgcattaaaagactgatgagaa
2821 ttcccctggacgccacactcctcgcagtaaaacgggtacggctccggcccgttggacttg
2881 cctcctttacctgggctctgcttcattgcaccggccctcgttgcaccggccctcgttgta
2941 ctggccctcgttgtgtctttctggtcaagggcatgcttctttagggtggtgaactgatcc
3001 acagaaacaaaactgttctgaacttggactcggccatctttgatgttgatcttgccattg
3061 gtcttcaaagcatagagtatgcttgaaaagttgaaacctattcctcctttctctaatctt
```

```
3121 ggcttgtattctttcaggaaagctgtcttcatttgctccttgctgtccacatctcctcga
3181 tcactctcttgaagaaacttgagaaacccgagtccgacacgtcgattctgagccaccgag
3241 ggcctgcctgttctctgattggtcatgacgtcaacacttttatcataacttcgcagcaat
3301 aattgctctccctcagctggtcagtttctgcaggcctggtaaaaacgtttatttagtcca
3361 cttggcttgctcgctctacgtcgtataaagaaaccccacatctgacacacgtgtgaaat
3421 aatagcagaaaccttgactcggccgccgggcagtggttttgttggtttttttctcacgt
3481 gtgtcaga
```

Fig. S36. ***A. rubens* Arnp21 precursor**. The nucleotide sequence (lowercase, 3488 bases) encoding the precursor protein (uppercase, 102 amino acid residues) is shown. The predicted signal peptide is represented in blue, putative Arnp21 peptides (with cysteine (C) residues underlined) is represented in red, C-terminal glycine (G) residues that are putative substrates for amidation are represented in orange and putative dibasic cleavage sites are represented in green. The asterisk shows the position of the stop codon. This sequence was determined from contig 1124521 and has been deposited in GenBank under accession number KT601738.

```
1                                                              tt
3    ggtatagcatacaagtagagtcacgagtctgccggaacttatgggtttctgcgtcaaact
63   gttttggcgggtaattcatgacttaaccgtgtacgtgtcggcgtcatagttgcatttgga
123  gatgccgcccgcctcatactccctcccacctacgccatcgtaccacctcttaaatctaca
183  agctcgtagatgaccgctccatagatcgtaaacgttgcactcataacacgctgagtgggt
243  ctagctaactgtcactactcgacgttgaaacccttcactgtgagagtctggtgtacggta
303  ctatcttacaggtgttcagtgggcataatatgcggacatgattacaacgtgtacggtcga
363  gtacaacaagtgatgagcattttcccgcggttttaacgtctccatgtagtagaacttaac
423  aaaagtgattgaattaactcatcagacaggcactaccaaagtttacgcgaacttgtgggt
483  tcaactaaagaaacaataacaatttacataatgcatattcaggcaatgacgtctttcgtt
                                M  H  I  Q  A  M  T  S  F  V    10
543  gtcgctttcatattggttagtttgacttcggggagtttcggctggcaaacaacagatgag
      V  A  F  I  L  V  S  L  T  S  G  S  F  G  W  Q  T  T  D  E    30
603  gaaaactatgacgtcaagatggttggttacccgaacctattccaccaccattctgcactc
      E  N  Y  D  V  K  M  V  G  Y  P  N  L  F  H  H  H  S  A  L    50
663  gacgtgcccataacgtcagaagaagtcgcggaactagacaatcgcataccgttacagcga
      D  V  P  I  T  S  E  E  V  A  E  L  D  N  R  I  P  L  Q  R    70
723  ttggctttacgtctactcgaggctaacaaagagggcgctgcatttcgacgagataccagg
      L  A  L  R  L  L  E  A  N  K  E  G  A  A  F  R  R  D  T  R    90
783  aatccccactactttccaagtaaaccaacatgtcacggtaactcatgtaaccaacgagga
      N  P  H  Y  F  P  S  K  P  T  C  H  G  N  S  C  N  Q  R  G   110
843  tggaaacgtagtctggcccccactggcggcaatggtgaggagtttcgaagggatcttcgg
      W  K  R  S  L  A  P  T  G  G  N  G  E  E  F  R  R  D  L  R   130
903  caacgctattccccgcgtaaaccaacatgccagggtaacacgtgtaaccaaagaggatgg
      Q  R  Y  S  P  R  K  P  T  C  Q  G  N  T  C  N  Q  R  G  W   150
963  aagcgcagctgggccactaagtaaacaaactcttggttttctagttaaactctggaactt
      K  R  S  W  A  T  K  *                                        157
1023 tattgcgtgtgtttggacggttttcatgtttgacaattcagtgcttttaagcaacaaagc
1083 tgcacaaaaagtatacattac
```

Fig. S37. ***A. rubens*** **Arnp22 precursor.** The nucleotide sequence (lowercase, 1103 bases) encoding the precursor protein (uppercase, 157 amino acid residues) is shown. The predicted signal peptide is represented in blue, putative Arnp22 peptides (with cysteine (C) residues underlined) is represented in red and putative dibasic cleavage sites are represented in green. The asterisk shows the position of the stop codon. This sequence was determined from contig 1098506 and has been deposited in GenBank under accession number KT601739.

```
1                                                               t
2    agactcgtccacagtcgcttggcagaacacaaccacccttaacttaatcatttcgataca
62   ggacaaactatatactgcatcagatcctcatagacaatctctttaatctattgcaagaaa
122  taataatctacagaaggacttgtttaagtctattgacgtccctggtttaaaataacgatc
182  gtgagtcctggtgggtttatagtttggaggcagggtgcttcgatagtcagttgtctctag
242  tgatcaaaacaacgtgaaccttcagtctacacgaacaccttgacgaacaccaaaccaaag
302  ctcatttctcacttcagtccagactctccaaagattgagttcaacttgcaagcaaggtgg
362  gatttaatcaacgtcaaaacagagataatcatgcagaactacattctctcaatcgctctg
                                               M  Q  N  Y  I  L  S  I  A  L   10
422  gtggtcgccatgttggcactgctcgccacgcccacaacctccgatgaagccctcaatggc
      V  V  A  M  L  A  L  L  A  T  P  T  T  S  D  E  A  L  N  G   30
482  gacatggaaatggacgaggaggcaatgcagacactgaaagaccttatagagcagtcgggg
      D  M  E  M  D  E  E  A  M  Q  T  L  K  D  L  I  E  Q  S  G   50
542  aaacgagtagagcgctcatggcgtaaaccatgccaggacaactcctgcatccagtctggg
      K  R  V  E  R  S  W  R  K  P  C  Q  D  N  S  C  I  Q  S  G   70
602  aatcgaggttggaagaggactggacaagccgcccgggatcctatctcagtcaaccaacgt
      N  R  G  W  K  R  T  G  Q  A  A  R  D  P  I  S  V  N  Q  R   90
662  ggtagccccttcagtatggacaactttaagtgtgttggtgaaagctgcagcagaggctgg
      G  S  P  F  S  M  D  N  F  K  C  V  G  E  S  C  S  R  G  W  110
722  aagcgaaccccagaacagaagcgttaagcaaccatccaaatgaggcggcaaccagatcac
      K  R  T  P  E  Q  K  R  *                                   118
782  tagtctagagaatataatttcaacgtggacaataatttaagatctaataattttcagttt
842  gtagaaatgagtcaatattttgtacgtgcaatacaaatcgtcaataatagtcgggtttta
902  ttcatcattgacttttt
```

Fig. S38. ***A. rubens*** **Arnp23 precursor**. The nucleotide sequence (lowercase, 918 bases) encoding the precursor protein (uppercase, 118 amino acid residues) is shown. The predicted signal peptide is represented in blue, putative Arnp23 peptides (with cysteine (C) residues underlined) are represented in red and putative dibasic cleavage sites are represented in green. The asterisk shows the position of the stop codon. This sequence was determined from contig 1089690 and has been deposited in GenBank under accession number KT601740.

```
1                                                                  g
2    cgaagtatgctgtacattgttgcgtttttggtgttcgttactgctacgagtggctttagc
             M  L  Y  I  V  A  F  L  V  F  V  T  A  T  S  G  F  S   18
62   gtcggagcggtagacggcaacccacctcgccgggagtacaacgatcctgtcggcgaagtt
      V  G  A  V  D  G  N  P  P  R  R  E  Y  N  D  P  V  G  E  V   38
122  tttgagtctatggagctaccttccaagctcccggagtctgagcagcaacacaatgcaaag
      F  E  S  M  E  L  P  S  K  L  P  E  S  E  Q  Q  H  N  A  K   58
182  gagaggcgttgttggtcagcaatccccaatatgtgttctggtacttttggcaaacggcga
      E  R  R  C  W  S  A  I  P  N  M  C  S  G  T  F  G  K  R  R   78
242  gtacgctcaacagtctaacccaagcataaaacggaccaataaaaggctgaacctgctagc
      V  R  S  T  V  *                                              83
302  ccttgcattgacatccacaaagaacgcctaccgccctctcgtggtctccatgagccaacg
362  cctcgcattcatcccactccgattagtcacgttgtgtctatacgtcatcaatggtcgcta
422  caatcaaccaatcagacagtgatctagccgccttcgttcatggactagtatccaatcaac
482  ggatagactgtgtaactatagtgttcaat
```

Fig. S39. ***A. rubens*** **Arnp24 precursor**. The nucleotide sequence (lowercase, 510 bases) encoding the precursor protein (uppercase, 83 amino acid residues) is shown. The predicted signal peptide is represented in blue, a putative Arnp24 peptide (with cysteine (C) residues underlined) is represented in red, a C-terminal glycine (G) residue that is a putative substrate for amidation is represented in orange and putative dibasic cleavage sites are represented in green. The asterisk shows the position of the stop codon. This sequence was determined from contig 1042300 and has been deposited in GenBank under accession number KT601741.

```
1     agatcacattcgctgattcacatttcaacgttaacgcaaggacagtgcgcaacctgactg
61    aggctcgtctttgcttatatactcgagtgtgcaagttcaagacagtaagcgaggaacacc
121   ttagagaggctgattgaagtgagatcgtacccgatcatcccaaaatacagaaacatggct
                                                          M  A     2
181   gtggtgccaggaaagacaaataacgggttggcgtggctgttgcttttcctgatggtatca
       V  V  P  G  K  T  N  N  G  L  A  W  L  L  L  F  L  M  V  S  22
241   acggtgtgcctaagaccatgccacgcaagcaggagatgctcggtcaagggatgtatggtt
       T  V  C  L  R  P  C  H  A  S  R  R  C  S  V  K  G  C  M  V  42
301   cacttcggcaaaagaatagctccatttcccgaacaagatgaagagcaagcagttcaaaaa
       H  F  G  K  R  I  A  P  F  P  E  Q  D  E  E  Q  A  V  Q  K  62
361   gctacggattatcaaaacgaacggcaaaacaacctgcttcgtctagtggaccgactcatc
       A  T  D  Y  Q  N  E  R  Q  N  N  L  L  R  L  V  D  R  L  I  82
421   tccaactcgatgacgtcacggaattctccaatagatgacgacgcatgattggaaacaatt
       S  N  S  M  T  S  R  N  S  P  I  D  D  D  A  *              97
481   cttgtatgaaactcggacttgtaattaaaatgattatatcgaacatacgtggaaggcatg
541   gaccgttatactttgcgtttactttataccttttcgagtttttgtgttaaatgatcgtag
601   accttatgcacatgacgtca
```

Fig. S40. ***A. rubens* Arnp25 precursor**. The nucleotide sequence (lowercase, 620 bases) encoding the precursor protein (uppercase, 97 amino acid residues) is shown. The predicted signal peptide is represented in blue, a putative Arnp25 peptide (with cysteine (C) residues underlined) is represented in red, a C-terminal glycine (G) residue that is a putative substrate for amidation is represented in orange and a putative dibasic cleavage site is represented in green. The asterisk shows the position of the stop codon. This sequence was determined from contig 1062339 and has been deposited in GenBank under accession number KT601742.
